# Supplementary material for: Combining visible-light induction and copper catalysis for chemo-selective nitrene transfer for late-stage amination of natural products
Source: Commun Chem. 2022 Jul 6;5:79. doi: 10.1038/s42004-022-00692-6 (PMC9814389; doi:10.1038/s42004-022-00692-6)
Supplement: Supplementary file 2 — Supplementary Information [file 42004_2022_692_MOESM2_ESM.pdf]

Supplementary Information

Combining visible-light induction and copper catalysis for chemo-selective nitrene transfer for late-stage amination of natural products

Dr. Qi Xing[1]*, Ding Jiang[1], Jiayin Zhang[1], Dr. Liangyu Guan[1], Dr. Ting Li[1], Dr. Yi Zhao[1], Man Di [1], Huangcan Chen[1], Dr. Chao Che[1,2]* & Dr. Zhendong Zhu[1]*

[1] BayRay Innovation Center, Shenzhen Bay Laboratory, Shenzhen 518132, China. [2] State Key Laboratory of Chemical Oncogenomics and Key Laboratory of Chemical Genomics, Peking University Shenzhen Graduate School, Shenzhen 518055, China.

*email: zhuzd@szbl.ac.cn; chec@pku.edu.cn; xingqi@szbl.ac.cn

Table of Contents

1) Supplementary Methods..........2

2) Supplementary Results and Discussion..........4

3) Supplementary Note1..........9

4) Supplementary Note 2..........21

5) Supplementary References..........74

Supplementary Methods

General Information

The reagents were purchased from commercial sources and used as received without further purification. All reactions were performed in a nitrogen-atmosphere glove box using dry solvents. Dry CH_3CN and 1,2-dichloroethane were purchased from J&K. $[Cu(OTf)]_2$·toluene was purchased from J&K and all the oxazoline ligands were purchased from Bidepharm. A micro photochemical reactor (12 W, capacity 16 vials, 435-445 nm) purchased from Aldrich was used for all photochemical reactions. Reactions were monitored by thin layer chromatography (TLC) carried out on 0.25 mm Tsingdao silica gel plates (GF-254) and visualized under UV light at 254 nm. Staining was performed with an ethanolic solution of phosphomolybdic acid (PMA) or by oxidative staining with an aqueous basic potassium permanganate (KMnO4) solution and subsequent heating. Preparative thin layer chromatography (TLC) was performed on 0.4-0.5 mm Jiangyou silica gel plates. NMR spectra of the products were recorded Brüker Advance 300 (1H: 300 MHz, 13C: 75 MHz), Brüker Advance 400 (1H: 400 MHz, 13C: 100 MHz) or Brüker Advance 500 (^{1}H: 500 MHz, ^{13}C: 126 MHz), and were calibrated using residual undertreated deuterium solvent as an internal reference ($CDCl_3$: ^{1}H NMR = 7.26 ppm, ^{13}C NMR = 77.16 ppm). The following abbreviations were used to explain the multiplicities: s = singlet, d = doublet, t = triplet, q = quartet, m = multiplet, br = broad, dd = doublet of doublets. High resolution mass spectra (HRMS) of the products were obtained on a Thermo Q Exactive Focus-spectrometer. The crude product was purified by preparative HPLC (1260 Infinity II+DAD+RID) to give the pure product if not pure enough by preparative TLC.

Preparation of substrates

Preparation methods for 1-iodo-2-(methoxymethyl)benzene: NaH (60% oil suspension, 8.6 g, 213.6 mmol) was added to a stirred solution of 2-iodobenzyl alcohol (12.5 g, 53.4 mmol) in dry DMF (250 mL) at 0 °C and the mixture was stirred at room temperature for 1 h. Then to the mixture was added MeI (13.3 mL, 213.6 mmol) dropwise and continue to stir at room temperature for 4 h. Finally, the resultant mixture was quenched with H_2O (125 mL) and extracted with hexane (3 × 100 mL). Combine the organic phases and concentrate under reduced pressure. The crude product was purified by column chromatography on silica gel (hexane: EtOAc 20:1) to give the desired product in 90% yield.

1-iodo-2-(methoxymethyl)benzene

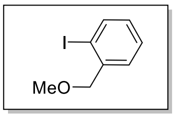

Colorless oil. ^{1}H NMR (300 MHz, $CDCl_3$) δ 7.83 (dd, J = 7.9, 0.9 Hz, 1H), 7.43 (dd, J = 7.6, 1.6 Hz, 1H), 7.35 (td, J = 7.5, 1.0 Hz, 1H), 6.99 (td, J = 7.7, 1.8 Hz, 1H), 4.45 (s, 2H), 3.48 (s, 3H). NMR spectra were consistent with literature data.[1]

Preparation methods for (2-(methoxymethyl)phenyl)-λ^3-iodanediyl diacetate: Following the reported procedure,[1] a mixture of peracetic acid (8.7% acetic acid solution, 15 mL) and 1-iodo-2-(methoxymethyl)benzene (2.4 g, 9.7 mmol) was stirred at 30 °C for 12 h, before quenching with H_2O (20 mL). The resultant mixture was extracted with CH_2Cl_2 (3 × 50 mL). The combined organic phase was dried over $MgSO_4$ and concentrated to give the title compound in 68% yield.

(2-(methoxymethyl)phenyl)-λ^3-iodanediyl diacetate

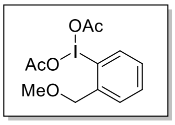

White solid. ^{1}H NMR (400 MHz, $CDCl_3$) δ 8.21 (d, *J* = 7.8 Hz, 1H), 7.70 (d, *J* = 6.7 Hz, 1H), 7.63 (t, *J* = 7.5 Hz, 1H), 7.41 – 7.35 (m, 1H), 4.72 (s, 2H), 3.47 (s, 3H), 1.97 (s, 6H). NMR spectra were consistent with literature data.[1]

Preparation methods for N-sulfonyliminoiodinanes: Powdered KOH (9.6 mmol) was added to a solution of amide (4.0 mmol) in dry MeOH (16 mL) and the mixture was stirred at room temperature for 30 min. Then the corresponding iodobenzene diacetate (4.0 mmol) was added at 0 ^{o}C and the resultant mixture was stirred at 0 ^{o}C for additional 3 h. Remove MeOH under reduced pressure and add ice-cold water (20 mL) to the residue. The generated white precipitate was collected by filtration and washed with H_2O and Et_2O to give the corresponding pure *N*-sulfonyliminoiodinanes.

N-((2-(methoxymethyl)phenyl)- λ^{3}-iodanylidene)-4-methylbenzenesulfonamide

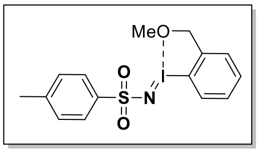

White solid. ^{1}H NMR (300 MHz, $CDCl_3$) δ 7.96 – 7.90 (m, 1H), 7.81 (d, *J* = 8.2 Hz, 2H), 7.35 (m, 2H), 7.17 (m, 3H), 4.64 (s, 2H), 3.47 (s, 3H), 2.34 (s, 3H). NMR spectra were consistent with literature data.[1]

4-methyl-N-(phenyl-λ^{3}-iodanylidene)benzenesulfonamide

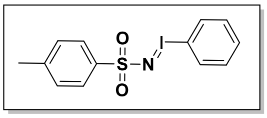

White solid. ^{1}H NMR (400 MHz, DMSO) δ 7.70 (d, *J* = 7.7 Hz, 2H), 7.51 – 7.42 (m, 3H), 7.31 (t, *J* = 7.7 Hz, 2H), 7.08 (d, *J* = 7.9 Hz, 2H), 2.29 (s, 3H). NMR spectra were consistent with literature data.[2]

Preparation methods for alkenes: KOH (17.9 mmol) was added to a solution of phenylacetalaldehydes (16.7 mmol) in 95% ethanol (8 mL) and the mixture was stirred under reflux for 12 h. The solvent was cooled to room temperature and concentrated in vacuo, the resultant residue was dissolved in H_2O (10 mL) and the mixture was extracted with ethyl acetate (3 × 20 mL). The combined organic phase was dried over $MgSO_4$ and concentrated in vacuo. The crude product was purified by column chromatography on silica gel (hexane) to give the desired product.

(*E*)-prop-1-ene-1,3-diyldibenzene

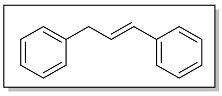

Colorless oil. ^{1}H NMR (400 MHz, $CDCl_3$) δ 7.46 – 7.25 (m, 10H), 6.45 (m, 2H), 3.62 (d, *J* = 6.6 Hz, 2H). ^{13}C NMR (100 MHz, $CDCl_3$) δ 140.2, 137.5, 131.1, 129.23, 128.7, 128.6, 127.2, 126.2 (two peaks overlapped), 39.4. NMR spectra were consistent with literature data.[3]

(*E*)-4,4'-(prop-1-ene-1,3-diyl)bis(chlorobenzene)

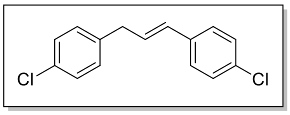

Colorless oil. ^{1}H NMR (400 MHz, $CDCl_3$) δ 7.31 – 7.25 (m, 6H), 7.17 (d, *J* = 8.5 Hz, 2H), 6.44 – 6.23 (m, 2H), 3.51 (d, *J* = 6.5 Hz, 2H). ^{13}C NMR (100 MHz, $CDCl_3$) δ 138.3, 135.8, 132.9, 132.1, 130.3, 130.1, 129.4, 128.7 (two peaks overlapped), 127.4, 38.6. NMR spectra were consistent with literature data.[3]

Supplementary Results and Discussion

Optimization of the reaction conditions for photo-induced aziridination of alkene:

Supplementary Table 1. Optimization of the reaction conditions for photo-induced aziridination of alkenes.[a,b]

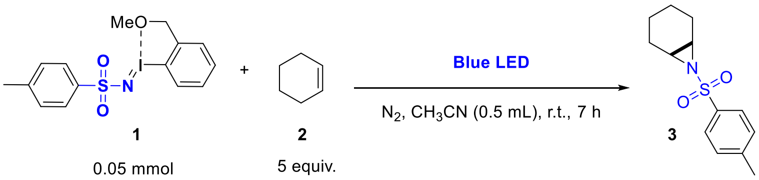

Entry	Change from the "standard conditions"	Yield [%]
1	None	79
2	In the dark	no reaction
3	white LED instead of blue LED	75
4	In air instead of N_2	72
5	PhI=NTs instead of **1**	66
6	DCE instead of CH_3CN	53
7	THF instead of CH_3CN	not detected
8	DMF instead of CH_3CN	not detected
9	1.0 equiv. of **2**	66
10	2.0 equiv. of **2**	72
11	prolong reaction time to 12 h	77

[a]Reaction conditions: **1** (0.05 mmol), **2** (0.25 mmol), in CH_3CN (0.5 mL) under 12 W blue LEDs (435-445 nm) at room temperature for 7 h. [b]Isolated yield.

Optimization of the reaction conditions for copper-catalyzed C-H amination of alkene:

Supplementary Table 2. Optimization of the reaction conditions for copper-catalyzed allylic C–H amidation-ligand screening.[a,b]

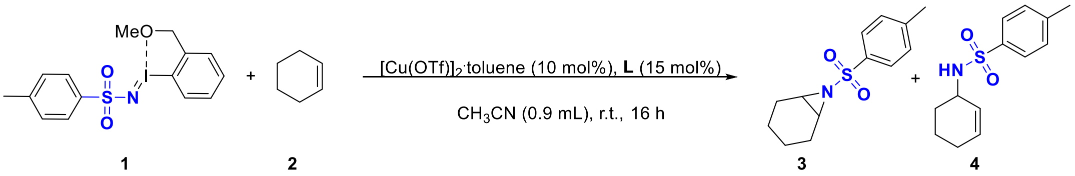

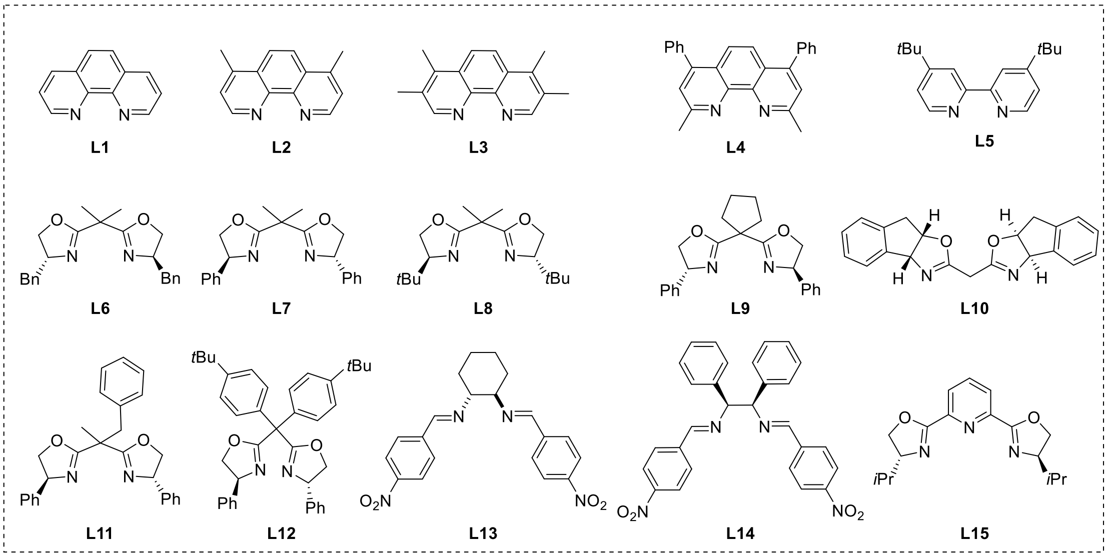

Entry	Ligand	Yield (%) 3	4	**3/4**
1	L_1	30	12	2.5/1
2	L_2	trace	trace	------
3	L_3	trace	trace	------
4	L_4	18	3	6/1
5	L_5	19	4	4.8/1
6	L_6	27	7	3.9/1
7	L_7	3	11	1/4
8	L_8	8	12	1/1.5
9	L_9	6	8	0.8/1
10	L_{10}	35	12	2.9/1
11	L_{11}	4	20	1/5

12	L_{12}	4	32	1/8
13	L_{13}	40	5	8/1
14	L_{14}	5	4	1/0.8
15	L_{15}	63	13	4.8/1

[a]Reaction conditions: **1** (0.1 mmol), **2** (0.5 mmol, 5.0 equiv.), $[Cu(OTf)]_2$·toluene (10 mol%), **L** (15 mol%), r.t., in CH_3CN (0.9 mL) under N_2 for 16 h. [b]Isolated yield.

Supplementary Table 3. Optimization of the reaction conditions for copper-catalyzed allylic C–H amidation- screening of solvents, metal/ligand ratios and alkene/iminoiodinane ratios. [a,b]

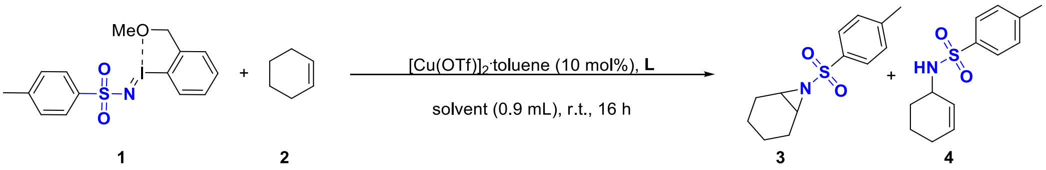

Entry	Solvent	Ligand (mol %)	Yield (%)		**3aa/4aa**
			**3**	**4**	
1	CH_3CN	L_7 (15 mol%)	3	11	1/4
2	DCE	L_7 (15 mol%)	4	30	1/7.5
3	DMSO	L_7 (15 mol%)	n.d.	n.d.	------
4	DMA	L_7 (15 mol%)	trace	trace	------
5	MeOH	L_7 (15 mol%)	n.d.	n.d.	------
6	NMP	L_7 (15 mol%)	trace	trace	------
7	DCE	L_8 (15 mol%)	4	34	1/8.5

**8**	**DCE**	**L_9** (15 mol%)	**4**	**44**	**1/11**
**9**[c]	**DCE**	**L_9** (15 mol%)	**6**	**61**	**1/10**
**10**[c]	**DCE**	**L_9** (10 mol%)	**10**	**47**	**1/5**
**11**[c]	**DCE**	**L_9** (20 mol%)	**9**	**65**	**1/7.1**
**12**[c]	**DCE**	**L_9** (25 mol%)	**9**	**70**	**1/8.3**
**13**[c,d]	**DCE**	**L_9** (15 mol%)	**5**	**54**	**1/10**
**14**[c,e]	**DCE**	**L_9** (15 mol%)	**5**	**57**	**1/11**

[a]Reaction conditions: **1** (0.1 mmol), **2** (0.5 mmol, 5 equiv.), $[Cu(OTf)]_2$·toluene (10 mol%), **L** (15 mol%), r.t., in solvent (0.9 mL) under N_2 for 16 h. [b]Isolated yield. [c]4ÅMS (100 mg) was added. [d] 1.0 equiv. of **2** was used. [e] 2.0 equiv. of **2** was used.

Supplementary Scheme 1. Copper-catalyzed amination of cyclohexene with different iminoiodinanes.

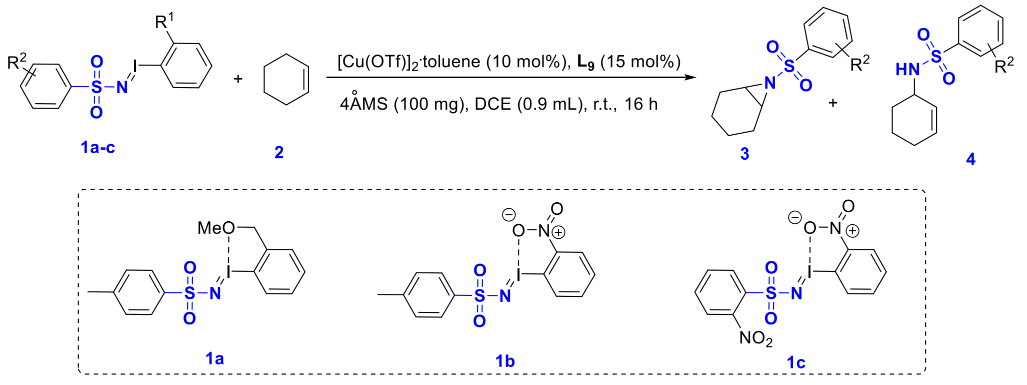

Entry	1a-c	yield of 3 (%)	yield of 4 (%)	ratio of 3 to 4 (3/4)
1	1a	6	61	1:10
2	1b	8	55	1:7.4
3	1c	Trace	Trace	—

Reaction conditions: **1a-c** (0.1 mmol), **2** (0.5 mmol, 5 equiv.), $[Cu(OTf)]_2$·toluene (10 mol%), **L_9** (15 mol%), 4ÅMS (100 mg), r.t., in DCE (0.9 mL) under N_2 for 16 h. Isolated yield.

Mechanistic Studies

Influence of TEMPO on the aziridination of cyclohexene:

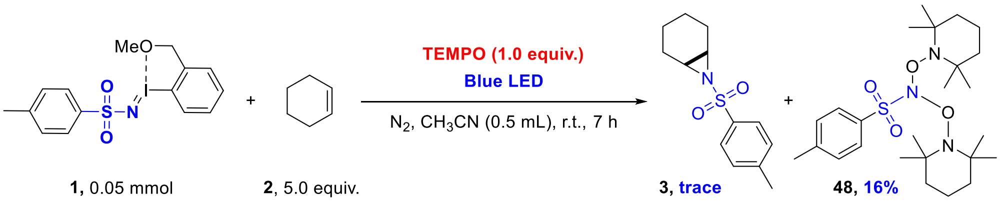

1, 0.05 mmol **2**, 5.0 equiv. **3**, **trace** **48**, **16%**

Experimental procedure: In a glovebox, a solution of **1a** (0.05 mmol, 21 mg) in CH_3CN (0.5 mL) was added into a 2.0 mL vial. Then cyclohexene (0.25 mmol, 21 mg) and TEMPO (0.05 mmol, 8.0 mg) were added and the resulting mixture was stirred under 12 W blue LED (435-445 nm) at room temperature for 7 h. The resultant reaction mixture was monitored by LC-MS and purified by preparative TLC. Only trace amount of aziridination product **3** was detected and a sulfonyl amide product **46** was isolated in 16% yield. **This result suggests the presence of radical intermediates in the aziridination process.**

Influence of TEMPO on the allylic C-H amination of cyclohexene:

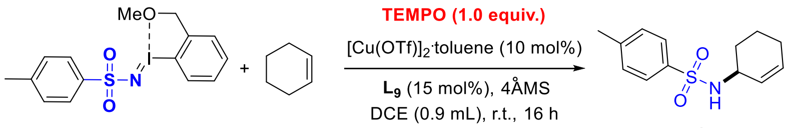

1, 0.1 mmol **2**, 5.0 equiv. **4**, 39%

Experimental procedure: In a glovebox, [Cu(OTf)]$_2$·toluene (10 mol%, 5.3 mg) and **L$_9$** (15 mol%, 5.4 mg) in CH_3CN (0.9 mL) was added into a 8.0 mL vial. The mixture was stirred for 30 min. Then 4ÅMS (100 mg), TEMPO (0.1 mmol, 15.6 mg), **2** (0.5 mmol, 41 mg) and **1** (0.1 mmol, 42 mg) were added successively. The resulting mixture was stirred at room temperature for 16 h. The resultant reaction mixture was purified by preparative TLC. The C-H amination product **4** was obtained in 39% yield. **This result suggests that radical intermediates may be involved in the reaction process.**

Aziridination of *cis*- or *trans*-1,2-diphenylethenes:

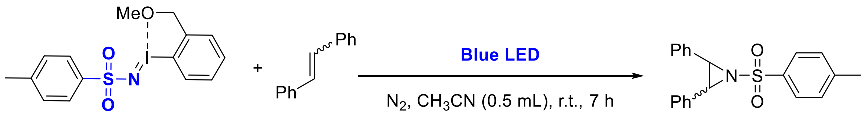

1 **49** **50**

**49**	yield of **50** (*syn* : *anti*)
cis-**49**	70% (15 : 85)
trans-**49**	77% (7 : 93)

Experimental procedure: In a glovebox, a solution of **1** (0.05 mmol, 21 mg) in CH_3CN (0.5 mL) was added into a 2.0 mL vial. Then 1,2-diphenylethene (*cis*- or *trans*-) (0.25 mmol, 45 mg) was added and the resulting mixture was stirred under 12 W blue LED (435-445 nm) at room temperature for 7 h. The resultant reaction mixture was monitored by ^1H NMR and purified by preparative TLC. The reaction of *cis*-**49** gave the aziridination product **50** in 70% yield with *syn*/*anti* ratio of 15 : 85, whereas the reaction of *trans*-**49** gave **50** in 77% yield with *syn*/*anti* ratio

of 7 : 93. **This result suggests a two-step mechanism, which allows isomerization to proceed, rather than a concerted nitrogen atom transfer.**

Study the activation of substrates by blue LED irradiation:

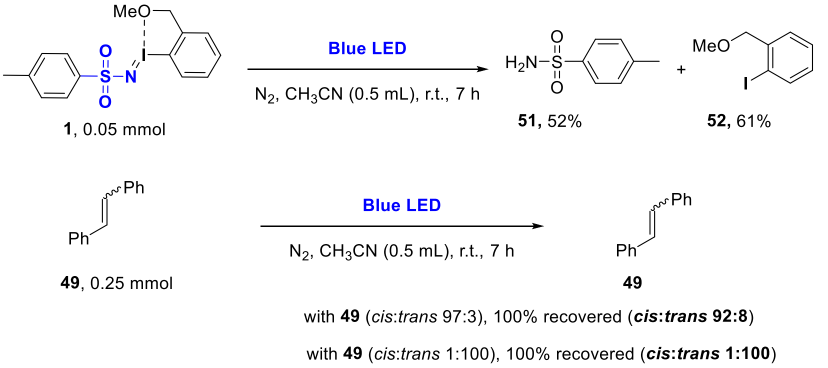

Experimental procedure: In a glovebox, a solution of **1** (0.05 mmol, 21 mg) or 1,2-diphenylethene **49** (*cis*- or *trans*) (0.25 mmol, 45 mg) in CH_3CN (0.5 mL) was added into a 2.0 mL vial. The resulting mixture was stirred under 12 W blue LED (435-445 nm) at room temperature for 7 h. The resultant reaction mixture was monitored by ^{1}H NMR and purified by preparative TLC. **1** was converted into sulfonamide in 52% yield along with 61% yield of 2-iodobenzyl ether and *cis*/*trans* ratio of **49** remains unchanged after the irradiation. **This result suggests photo induced activation of iminoiodinane, rather than the alkene substrates.**

Allylic isomerization studies:

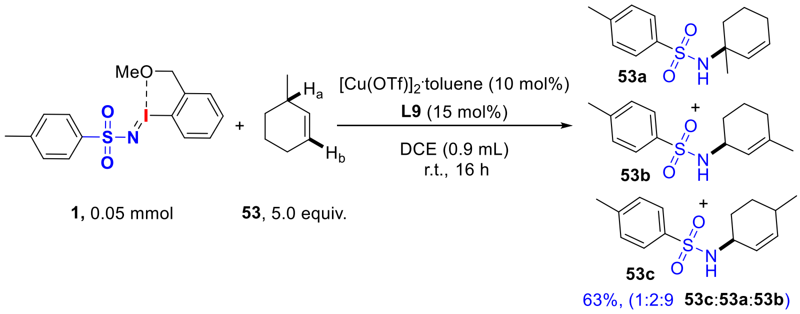

Experimental procedure: In a glovebox, $[Cu(OTf)]_2$·toluene (10 mol%, 5.3 mg) and $\mathbf{L_9}$ (15 mol%, 5.4 mg) in CH_3CN (0.9 mL) was added into a 8.0 mL vial. The mixture was stirred for 30 min. Then 4ÅMS (100 mg), **53** (0.5 mmol, 48 mg) and **1** (0.1 mmol, 42 mg) were added successively. The resulting mixture was stirred at room temperature for 16 h. The resultant reaction mixture was purified by preparative TLC. The C-H amination products **53a**, **53b** and **53c** were obtained as a mixture in 63% yield. **The generation of double bond-transposed product suggests the involvement of radical intermediates in the reaction process.**

Supplementary Note1

Experimental Procedure and Characterization Data for Products

General procedure for photo-induced aziridination of alkenes: In a glovebox, a solution of **1** (0.05 mmol, 21 mg) in CH_3CN (0.5 mL) was added into a 2.0 mL vial. Then alkene (0.25 mmol of simple alkenes or 0.1 mmol of complex natural products) was added and the resulting mixture was stirred under 12 W blue LED (435-445 nm) at room temperature for 7 h. The resultant reaction mixture was purified by preparative TLC (hexane/EA 10:1 to 5:1).

7-tosyl-7-azabicyclo[4.1.0]heptane (3)

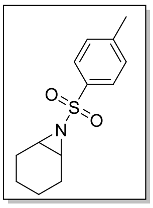

White solid. Melting point: 57.2 – 58.2 ºC. ^{1}H NMR (300 MHz, $CDCl_3$) δ 7.81 (d, *J* = 8.3 Hz, 2H), 7.32 (d, *J* = 8.0 Hz, 2H), 3.05 – 2.85 (m, 2H), 2.44 (s, 3H), 1.78 (dd, *J* = 8.4, 3.2 Hz, 4H), 1.39 (m, 2H), 1.20 (m, 2H). ^{13}C NMR (100 MHz, $CDCl_3$) δ 143.9, 135.8, 129.5, 127.5, 39.7, 22.7, 21.5, 19.3. **HRMS**(m/z) calc. for $C_{13}H_{17}NNaO_2S$: $[M+Na]^+$, 274.0872; Found: 274.0871.

6-tosyl-6-azabicyclo[3.1.0]hexane (8)

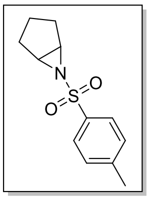

White solid. Melting point: 68.5 – 69.5 ºC. ^{1}H NMR (400 MHz, $CDCl_3$) δ 7.82 (d, *J* = 8.3 Hz, 2H), 7.33 (d, *J* = 8.0 Hz, 2H), 3.34 (s, 2H), 2.45 (s, 3H), 2.00 – 1.90 (m, 2H), 1.67 – 1.53 (m, 3H), 1.45 – 1.32 (m, 1H). ^{13}C NMR (100 MHz, $CDCl_3$) δ 144.0, 135.9, 129.5, 127.5, 46.7, 26.9, 21.6, 19.5. **HRMS**(m/z) calc. for $C_{12}H_{16}NO_2S$: $[M+H]^+$, 238.0896; Found: 238.0896.

8-tosyl-8-azabicyclo[5.1.0]octane (9)

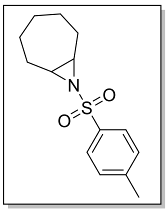

White solid. Melting point: 73.9 – 74.9 ºC. ^{1}H NMR (400 MHz, $CDCl_3$) δ 7.86 – 7.78 (m, 2H), 7.32 (d, *J* = 8.0 Hz, 2H), 3.01 – 2.90 (m, 2H), 2.45 (s, 3H), 1.92 – 1.75 (m, 4H), 1.62 – 1.39 (m, 5H), 1.23 – 1.12 (m, 1H). ^{13}C NMR (100 MHz, $CDCl_3$) δ 143.9, 135.9, 129.5, 127.5, 44.2, 31.0, 28.0, 25.1, 21.6. **HRMS**(m/z) calc. for $C_{14}H_{19}NNaO_2S$: $[M+Na]^+$, 288.1029; Found: 288.1028.

3-tosyl-3-azatricyclo[3.2.1.02,4]octane (10)

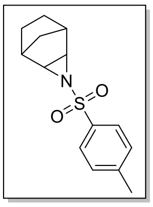

White solid. Melting point: 120.9 – 121.9 ºC. ^{1}H NMR (400 MHz, $CDCl_3$) δ 7.81 (d, *J* = 8.3 Hz, 2H), 7.32 (d, *J* = 8.0 Hz, 2H), 2.91 (s, 2H), 2.44 (s, 5H), 1.52 – 1.41 (m, 3H), 1.29 – 1.19 (m, 2H), 0.75 (d, *J* = 10.1 Hz, 1H). ^{13}C NMR (100 MHz, $CDCl_3$) δ 144.0, 135.8, 129.5, 127.6, 41.9, 35.7, 28.2, 25.5, 21.5. **HRMS**(m/z) calc. for $C_{14}H_{17}NNaO_2S$: $[M+Na]^+$, 286.0872; Found: 286.0871.

1-methyl-7-tosyl-7-azabicyclo[4.1.0]heptane (11)

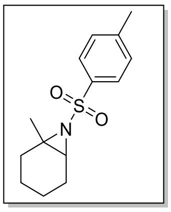

White solid. Melting point: 85.1 – 86.1 ºC. ^{1}H NMR (400 MHz, $CDCl_3$) δ 7.85 (d, *J* = 8.3 Hz, 2H), 7.33 (d, *J* = 8.1 Hz, 2H), 3.09 (d, *J* = 5.2 Hz, 1H), 2.46 (s, 3H), 2.09 (dt, *J* = 14.2, 4.8 Hz, 1H), 1.91 – 1.80 (m, 1H), 1.75 (s, 3H), 1.63 – 1.52 (m, 2H), 1.48 – 1.29 (m, 3H), 1.21 – 1.08 (m, 1H). ^{13}C NMR (100 MHz, $CDCl_3$) δ 143.4, 139.0, 129.4, 127.0, 51.3, 47.4, 32.1, 22.9, 21.6, 20.6, 19.9, 19.6. **HRMS**(m/z) calc. for $C_{14}H_{19}NNaO_2S$: $[M+Na]^+$, 288.1029; Found: 288.1028.

3-ethyl-2,2-dimethyl-1-tosylaziridine (12)

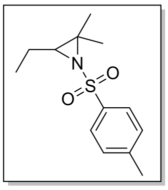

White solid. Melting point: 90.4 – 91.4 ^{o}C. ^{1}H NMR (400 MHz, $CDCl_3$) δ 7.83 (d, *J* = 8.3 Hz, 2H), 7.30 (d, *J* = 8.0 Hz, 2H), 2.81 (dd, *J* = 7.9, 5.7 Hz, 1H), 2.43 (s, 3H), 1.72 (s, 3H), 1.53 – 1.44 (m, 1H), 1.39 – 1.31 (m, 1H), 1.29 (s, 3H), 0.79 (t, *J* = 7.5 Hz, 3H). ^{13}C NMR (100 MHz, $CDCl_3$) δ 143.4, 138.3, 129.3, 127.3, 54.2, 51.9, 21.4 (two peaks overlapped), 21.1 (two peaks overlapped), 11.6. **HRMS**(m/z) calc. for $C_{13}H_{19}NNaO_2S$: $[M+Na]^+$, 276.1029; Found: 276.1029.

2-(2-bromoethyl)-1-tosylaziridine (13)

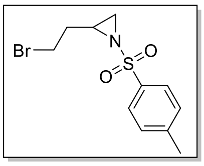

Colorless oil. ^{1}H NMR (400 MHz, $CDCl_3$) δ 7.85 (d, *J* = 8.3 Hz, 2H), 7.36 (d, *J* = 8.0 Hz, 2H), 3.32 (m, 1H), 3.15 (m, 1H), 2.92 (m, 1H), 2.73 (d, *J*= 7.0 Hz, 1H), 2.47 (s, 3H), 2.18 (d, *J* = 4.5 Hz, 1H), 2.17 – 2.09 (m, 1H), 1.84 (m, 1H). ^{13}C NMR (100 MHz, $CDCl_3$) δ 144.7, 134.5, 129.7, 128.1, 38.5, 34.5, 33.3, 29.2, 21.6. **HRMS**(m/z) calc. for $C_{11}H_{14}BrNNaO_2S$: $[M+Na]^+$, 325.9821; Found: 325.9821.

2-hexyl-1-tosylaziridine (14)

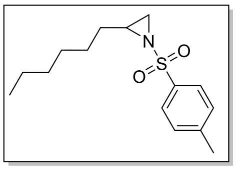

Colorless oil. ^{1}H NMR (500 MHz, $CDCl_3$) δ 7.83 (d, *J* = 8.3 Hz, 2H), 7.34 (d, *J* = 8.0 Hz, 2H), 2.72 (tt, *J* = 7.4, 4.8 Hz, 1H), 2.65 (d, *J* = 7.0 Hz, 1H), 2.45 (s, 3H), 2.07 (d, *J* = 4.6 Hz, 1H), 1.54 (m, 1H), 1.32 (m, 1H), 1.24 – 1.19 (m, 6H), 1.18 – 1.13 (m, 2H), 0.86 (t, *J* = 7.1 Hz, 3H). ^{13}C NMR (100 MHz, $CDCl_3$) δ 144.3, 135.1, 129.5, 127.9, 40.4, 33.7, 31.5, 31.2, 28.6, 26.7, 22.4, 21.6, 14.0. **HRMS**(m/z) calc. for $C_{15}H_{23}NNaO_2S$: $[M+Na]^+$, 304.1342; Found: 304.1341.

2-ethyl-3-methyl-1-tosylaziridine (15)[4]

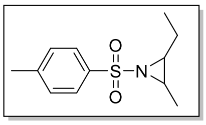

White solid. Melting point: 61.2 – 62.2 ^{o}C. The product was obtained as a mixture of *syn*- and *anti*- isomers. The *anti*/*syn* ratio is 2:1. ^{1}H NMR (400 MHz, $CDCl_3$) δ 7.86 – 7.77 (m, 2H, from *cis* and *anti*), 7.31 (t, *J* = 7.0 Hz, 2H, from *cis* and *anti*), 2.99 – 2.87 (m, 0.31H, from *cis*), 2.73 – 2.63 (m, 1.52H, from *cis* and *anti*), 2.43 (s, 0.96H, from *cis*), 2.42 (s, 2H, from *anti*), 1.76 – 1.61 (m, 1H, from *cis* and *anti*), 1.55 (d, *J* = 5.7 Hz, 2H, from *anti*), 1.53 – 1.35 (m, 1H, from *cis* and *anti*), 1.20 (d, *J* = 5.9 Hz, 1H, from *cis*), 0.84 (t, *J* = 7.4 Hz, 3H, from *cis* and *anti*). ^{13}C NMR (100 MHz, $CDCl_3$) δ 144.2, 143.8, 138.1, 135.6, 129.6, 129.5, 127.9, 127.4, 51.0, 46.6, 45.7, 40.4, 23.9, 21.7, 21.6, 19.9, 14.8, 11.9, 11.5, 11.4. **HRMS**(m/z) calc. for $C_{12}H_{18}NO_2S$: $[M+H]^+$, 240.1053; Found: 240.1053.

2-methyl-3-propyl-1-tosylaziridine (16)[4]

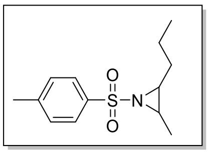

Colorless oil. The product was obtained as a mixture of *syn*- and *anti*- isomers. The *anti*/*syn* ratio is 2.2:1. ^{1}H NMR (400 MHz, $CDCl_3$) δ 7.82 (m, 2H, from *cis* and *anti*), 7.31 (m, 2H, from *cis* and *anti*), 2.96 – 2.85 (m, 0.3H, from *cis*), 2.80 – 2.72 (m, 0.3H, from *cis*), 2.72 – 2.63 (m, 1.3H, from *anti*), 2.43 (m, 3H, from *cis* and *anti*), 1.62 – 1.56 (m, 1.4H, from *anti*), 1.53 (d, *J* = 5.4 Hz, 2H, from *anti*), 1.45 – 1.37 (m, 0.7H, from *cis*), 1.33 – 1.25 (m, 2.4H, from *cis* and *anti*), 1.19 (d, *J* = 5.9 Hz, 1H, from *cis*), 0.87 (m, 3H, from *cis* and *anti*). ^{13}C NMR (100 MHz, $CDCl_3$) δ 144.2, 143.8, 138.2, 135.6, 129.6, 129.5, 127.8, 127.3, 49.6, 45.8, 45.0, 40.3, 32.4, 28.5, 21.6, 20.5, 20.4, 14.8, 13.7 (two peaks overlapped), 12.0.

HRMS(m/z) calc. for $C_{13}H_{20}NO_2S$: $[M+H]^+$, 254.1209; Found: 254.1209.

methyl 1-tosylaziridine-2-carboxylate (17)

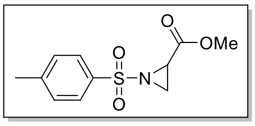

Colorless oil. ^1H NMR (400 MHz, $CDCl_3$) δ 7.86 (d, J = 8.4 Hz, 2H), 7.37 (d, J = 8.0 Hz, 2H), 3.75 (s, 3H), 3.36 (dd, J = 7.0, 4.1 Hz, 1H), 2.78 (d, J = 7.1 Hz, 1H), 2.57 (d, J = 4.1 Hz, 1H), 2.47 (s, 3H). ^{13}C NMR (100 MHz, $CDCl_3$) δ 167.2, 145.2, 133.9, 129.8, 128.2, 52.8, 35.6, 32.0, 21.6. **HRMS**(m/z) calc. for $C_{11}H_{13}NNaO_4S$: $[M+Na]^+$, 278.0457; Found: 278.0457.

7-tosyl-7-azabicyclo[4.1.0]hept-3-ene (18)

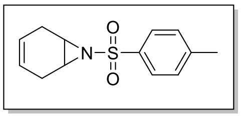

White solid. Melting point: 100.2 – 101.2 ºC. ^1H NMR (400 MHz, $CDCl_3$) δ 7.84 (d, J = 8.3 Hz, 2H), 7.33 (d, J = 8.0 Hz, 2H), 5.45 (s, 2H), 3.19 – 3.04 (m, 2H), 2.45 (s, 3H), 2.37 (m, 4H). ^{13}C NMR (100 MHz, $CDCl_3$) δ 144.1, 135.7, 129.6, 127.6, 121.6, 38.6, 23.0, 21.6. **HRMS**(m/z) calc. for $C_{13}H_{15}NNaO_2S$: $[M+Na]^+$, 272.0716; Found: 272.0715.

1-tosyl-1a,2,3,7b-tetrahydro-1H-naphtho[1,2-b]azirine (19)

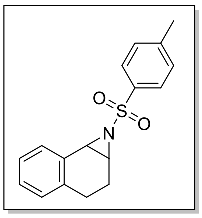

White solid. Melting point: 118.9 – 119.9 ºC. ^1H NMR (400 MHz, $CDCl_3$) δ 7.82 (d, J = 8.3 Hz, 2H), 7.30 (m, 3H), 7.25 – 7.14 (m, 2H), 7.05 (d, J = 7.3 Hz, 1H), 3.82 (d, J = 7.0 Hz, 1H), 3.56 (d, J = 7.0 Hz, 1H), 2.82 – 2.69 (m, 1H), 2.54 (dd, J = 15.6, 5.3 Hz, 1H), 2.42 (s, 3H), 2.26 (m, 1H), 1.69 (m, 1H). ^{13}C NMR (100 MHz, $CDCl_3$) δ 144.3, 136.7, 135.6, 130.0, 129.7, 129.5, 128.6, 128.5, 127.6, 126.4, 42.1, 41.8, 24.7, 21.7, 20.0. **HRMS**(m/z) calc. for $C_{17}H_{17}NNaO_2S$: $[M+Na]^+$, 322.0872; Found: 322.0873.

2-phenyl-1-tosylaziridine (20)

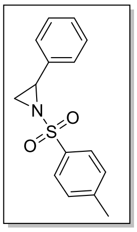

White solid. Melting point: 85.2 – 86.2 ºC. ^1H NMR (500 MHz, $CDCl_3$) δ 7.88 (d, J = 8.3 Hz, 2H), 7.34 (d, J = 8.0 Hz, 2H), 7.32 – 7.26 (m, 3H), 7.25 – 7.20 (m, 2H), 3.79 (dd, J = 7.2, 4.5 Hz, 1H), 2.99 (d, J = 7.2 Hz, 1H), 2.44 (s, 3H), 2.40 (d, J = 4.5 Hz, 1H). ^{13}C NMR (100 MHz, $CDCl_3$) δ 144.6, 134.9 (two peaks overlapped), 129.7, 128.5, 128.2, 127.9, 126.5, 41.0, 35.9, 21.6. **HRMS**(m/z) calc. for $C_{15}H_{15}NNaO_2S$: $[M+Na]^+$, 296.0716; Found: 296.0715.

2-(2-bromophenyl)-1-tosylaziridine (21)

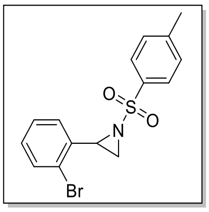

White solid. Melting point: 107.5 – 108.5 ºC. ^1H NMR (400 MHz, $CDCl_3$) δ 7.90 (d, J = 8.3 Hz, 2H), 7.51 (d, J = 7.8 Hz, 1H), 7.36 (d, J = 8.1 Hz, 2H), 7.25 – 7.10 (m, 3H), 3.99 (dd, J = 7.2, 4.4 Hz, 1H), 3.03 (d, J = 7.3 Hz, 1H), 2.45 (s, 3H), 2.27 (d, J = 4.4 Hz, 1H). ^{13}C NMR (100 MHz, $CDCl_3$) δ 144.8, 134.7, 134.6, 132.3, 129.8, 129.5, 128.1, 127.8, 127.5, 123.2, 41.2, 35.8, 21.6. **HRMS**(m/z) calc. for $C_{15}H_{14}BrNNaO_2S$: $[M+Na]^+$, 373.9821; Found: 373.9822.

2-(4-fluorophenyl)-1-tosylaziridine (22)

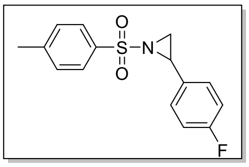

White solid. Melting point: 81.5 – 82.5 ºC. ^{1}H NMR (400 MHz, $CDCl_3$) δ 7.86 (d, *J* = 8.2 Hz, 2H), 7.34 (d, *J* = 8.1 Hz, 2H), 7.19 (m, 2H), 6.98 (m, 2H), 3.75 (dd, *J* = 7.0, 4.5 Hz, 1H), 2.97 (d, *J* = 7.2 Hz, 1H), 2.44 (s, 3H), 2.35 (d, *J* = 4.4 Hz, 1H). ^{13}C NMR (100 MHz, $CDCl_3$) δ 162.7 (d, *J* = 246 Hz, *ipso-Ph*-F), 144.8, 134.9, 130.9 (d, *J* = 2.9 Hz, *p-Ph*-F), 129.8, 128.3 (d, *J* = 8.3 Hz, *m-Ph*-F), 128.0, 115.7 (d, *J* = 22 Hz, *o-Ph*-F), 40.4, 36.1, 21.7. **HRMS**(m/z) calc. for $C_{15}H_{15}FNO_2S$: $[M+H]^+$, 292.0802; Found: 292.0802.

1-tosyl-2-(3-(trifluoromethyl)phenyl)aziridine (23)

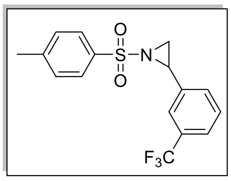

Colorless oil. ^{1}H NMR (400 MHz, $CDCl_3$) δ 7.91 – 7.85 (m, 2H), 7.53 (m, 1H), 7.43 (m, 3H), 7.35 (d, *J* = 8.0 Hz, 2H), 3.81 (dd, *J* = 7.2, 4.3 Hz, 1H), 3.01 (d, *J* = 7.2 Hz, 1H), 2.44 (s, 3H), 2.38 (d, *J* = 4.3 Hz, 1H). ^{13}C NMR (100 MHz, $CDCl_3$) δ 145.0, 136.3, 134.6, 131.0 (q, *J* = 32.0 Hz), 130.0, 129.9, 129.1, 128.0, 125.2 (q, *J* = 4.0 Hz), 123.4 (q, *J* = 4.0 Hz), 122.5, 40.1, 36.2, 21.7. **HRMS**(m/z) calc. for $C_{16}H_{15}F_3NO_2S$: $[M+H]^+$, 342.0770; Found: 342.0771.

***trans*-2-benzyl-3-phenyl-1-tosylaziridine (24)**

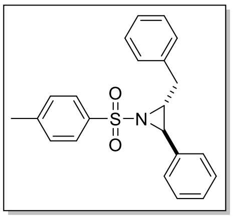

White solid. Melting point: 107.1 – 108.1 ºC. ^{1}H NMR (400 MHz, $CDCl_3$) δ 7.80 – 7.70 (m, 2H), 7.25 – 7.12 (m, 10H), 7.03 (dd, *J* = 6.7, 2.9 Hz, 2H), 3.93 (d, *J* = 4.3 Hz, 1H), 3.53 (dd, *J* = 14.5, 4.1 Hz, 1H), 3.34 (dd, *J* = 14.5, 9.5 Hz, 1H), 2.99 (dt, *J* = 9.5, 4.2 Hz, 1H), 2.33 (s, 3H). ^{13}C NMR (100 MHz, $CDCl_3$) δ 144.1, 137.8, 137.6, 135.1, 129.6, 128.8, 128.7, 128.5, 128.2, 127.4, 126.9, 126.5, 52.8, 49.1, 34.8, 21.6. **HRMS**(m/z) calc. for $C_{22}H_{22}NO_2S$: $[M+H]^+$, 364.1366; Found: 364.1364.

(1S,6S)-2,7,7-trimethyl-3-tosyl-3-azatricyclo[4.1.1.02,4]octane (25a)

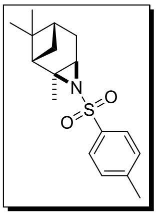

White solid. Melting point: 54.1 – 55.1 ºC. ^{1}H NMR (300 MHz, $CDCl_3$) δ 7.81 (d, *J* = 8.3 Hz, 2H), 7.32 (d, *J* = 8.0 Hz, 2H), 3.05 – 2.85 (m, 2H), 2.44 (s, 3H), 1.78 (dd, *J* = 8.4, 3.2 Hz, 4H), 1.39 (m, 2H), 1.20 (m, 2H). ^{13}C NMR (100 MHz, $CDCl_3$) δ 143.9, 135.8, 129.5, 127.5, 39.7, 22.7, 21.5, 19.3. **HRMS**(m/z) calc. for $C_{13}H_{17}NNaO_2S$: $[M+Na]^+$, 274.0872; Found: 274.0871.

2-((1S,3R,6R)-6-methyl-7-tosyl-7-azabicyclo[4.1.0]heptan-3-yl)propan-2-yl acetate (26a)

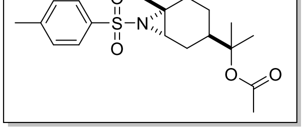

White solid. Melting point: 77.4 – 78.4 ºC. ^{1}H NMR (500 MHz, $CDCl_3$) δ 7.84 – 7.81 (m, 2H), 7.31 (d, *J* = 8.0 Hz, 2H), 3.18 – 3.16 (m, 1H), 2.43 (s, 3H), 1.99 (m, 1H), 1.90 (s, 3H), 1.88 – 1.80 (m, 3H), 1.73 (s, 3H), 1.56 – 1.46 (m, 2H), 1.36 (s, 3H), 1.32 (s, 3H), 1.03 (m, 1H). ^{13}C NMR (100 MHz, $CDCl_3$) δ 170.0, 143.3, 138.8, 129.3, 126.8, 83.7, 50.7, 48.7, 38.6, 31.9, 24.8, 23.1, 22.5, 22.2, 21.5, 20.8. **HRMS**(m/z) calc. for $C_{19}H_{27}NNaO_4S$: $[M+Na]^+$, 388.1553; Found: 388.1553.

2-((1R,3R,6S)-6-methyl-7-tosyl-7-azabicyclo[4.1.0]heptan-3-yl)propan-2-yl acetate (26b)

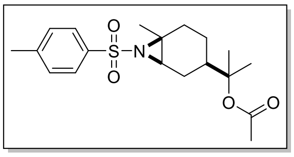

White solid. Melting point: 79.2 – 80.2 ºC. ^{1}H NMR (500 MHz, $CDCl_3$) δ^{1}H NMR (500 MHz, $CDCl_3$) δ 7.81 (d, *J* = 8.0 Hz, 2H), 7.30 (d, *J* = 7.8 Hz, 2H), 2.43 (s, 3H), 2.31 (d, *J* = 12.5 Hz, 2H), 2.06 (m, 2H), 2.01 (s, 3H), 1.86 (m, 2H), 1.51 (m, 8H), 1.43 (m, 2H), 1.34 (m, 1H). ^{13}C NMR (126 MHz, $CDCl_3$) δ 170.5, 143.3, 139.6, 129.4, 127.0, 79.9, 56.8, 52.9, 35.8, 25.8, 24.7, 22.4, 21.6, 19.8. **HRMS**(m/z) calc. for $C_{19}H_{27}NNaO_4S$: $[M+Na]^+$, 388.1553; Found: 388.1552.

(1R,4R,6S)-1-methyl-4-(prop-1-en-2-yl)-7-tosyl-7-azabicyclo[4.1.0]heptane (27a)[5]

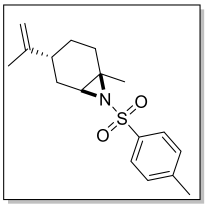

Colorless oil. ^{1}H NMR (400 MHz, $CDCl_3$) δ 7.83 (d, *J* = 8.3 Hz, 2H), 7.31 (d, *J* = 8.0 Hz, 2H), 4.74 (d, *J* = 1.3 Hz, 1H), 4.61 (s, 1H), 3.17 (dd, *J* = 3.9, 1.6 Hz, 1H), 2.44 (s, 3H), 1.97 (m, 2H), 1.85 – 1.63 (m, 9H), 1.57 – 1.50 (m, 1H), 1.26 – 1.17 (m, 1H). ^{13}C NMR (100 MHz, $CDCl_3$) δ 148.2, 143.4, 138.8, 129.4, 126.9, 109.4, 50.6, 48.1, 36.1, 30.4, 28.4, 25.1, 21.5, 21.1, 20.6. **HRMS**(m/z) calc. for $C_{17}H_{23}NNaO_2S$: $[M+Na]^+$, 328.1342; Found: 328.1342.

2-methyl-2-(4-methylcyclohex-3-en-1-yl)-1-tosylaziridine2-methyl-2-(4-methylcyclohex-3-en-1-yl)-1-tosylaziridine (27b)[5]

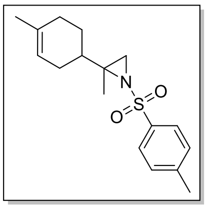

Colorless oil. ^{1}H NMR (400 MHz, $CDCl_3$) δ 7.83 (dd, *J* = 8.3, 3.9 Hz, 2H), 7.36 – 7.28 (m, 2H), 5.35 (s, 1H), 2.61 (d, *J* = 1.3 Hz, 1H), 2.44 (s, 3H), 2.26 (d, *J* = 11.8 Hz, 1H), 2.05 – 1.75 (m, 5H), 1.68 – 1.61 (m, 6H), 1.48 – 1.33 (m, 2H). ^{13}C NMR (100 MHz, $CDCl_3$) δ 143.6, 138.1, 134.1, 133.6, 129.3, 127.3, 119.9, 119.5, 53.7, 53.4, 41.5, 40.6, 40.4, 30.3, 30.1, 27.3, 27.2, 25.1, 25.0, 23.3, 21.5, 15.1, 14.9. **HRMS**(m/z) calc. for $C_{17}H_{23}NNaO_2S$: $[M+Na]^+$, 328.1342; Found: 328.1342.

(E)-4-(2,2,6-trimethyl-7-tosyl-7-azabicyclo[4.1.0]heptan-1-yl)but-3-en-2-one (42a)

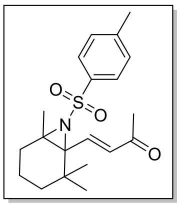

White solid. Melting point: 115.2 – 116.2 ºC. ^{1}H NMR (400 MHz, $CDCl_3$) δ 7.85 (d, *J* = 8.2 Hz, 2H), 7.33 (d, *J* = 8.1 Hz, 2H), 7.10 (d, *J* = 16.8 Hz, 1H), 6.23 (d, *J* = 16.8 Hz, 1H), 2.45 (s, 3H), 2.37 (s, 3H), 1.85 – 1.73 (m, 1H), 1.63 (dd, *J* = 9.4, 5.1 Hz, 1H), 1.59 (s, 3H), 1.57 – 1.51 (m, 1H), 1.39 (tt, *J* = 9.1, 4.7 Hz, 2H), 1.01 (dt, *J* = 13.0, 4.2 Hz, 1H), 0.92 (s, 3H), 0.87 (s, 3H). ^{13}C NMR (100 MHz, $CDCl_3$) δ 197.9, 143.6, 139.0, 138.8, 135.2, 129.5, 127.2, 62.1, 53.7, 34.6, 34.5, 30.1, 27.4, 26.9, 24.7, 21.5, 20.0, 16.5. **HRMS**(m/z) calc. for $C_{20}H_{27}NNaO_3S$: $[M+Na]^+$, 384.1604; Found: 384.1603.

6-methyl-2-(6-methyl-7-tosyl-7-azabicyclo[4.1.0]heptan-3-yl)hept-5-en-2-yl acetate (43a)

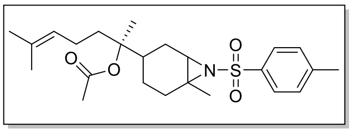

Colorless oil. ^{1}H NMR (400 MHz, $CDCl_3$) δ 7.83 (d, *J* = 8.3 Hz, 2H), 7.29 (d, *J* = 8.0 Hz, 2H), 4.95 (m, 1H), 3.15 (s, 1H), 2.41 (s, 3H), 2.07 – 1.94 (m, 2H), 1.92 (s, 3H), 1.88 – 1.83 (m, 1H), 1.75 (s, 3H), 1.70 (m, 1H), 1.65 (s, 3H), 1.60 (m, 2H), 1.57 – 1.52 (m, 1H), 1.47 (s, 3H), 1.43 – 1.35 (m, 1H), 1.31 (s, 3H), 1.26 (m, 2H), 1.02 (m, 1H). **HRMS**(m/z) calc. for $C_{24}H_{35}NNaO_4S$: $[M+Na]^+$, 456.2179; Found: 456.2182.

4-(3,3-dimethyl-1-tosylaziridin-2-yl)-2-(4-methylcyclohex-3-en-1-yl)butan-2-yl acetate (43b)

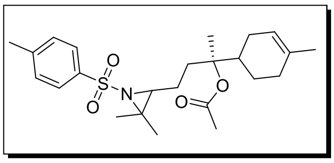

Colorless oil. ^{1}H NMR (400 MHz, $CDCl_3$) δ 7.86 – 7.81 (m, 2H), 7.30 (d, *J* = 8.0 Hz, 2H), 5.30 (t, *J* = 18.4 Hz, 1H), 2.81 (dd, *J* = 8.2, 5.2 Hz, 1H), 2.42 (s, 3H), 2.27 – 2.08 (m, 1H), 1.99 – 1.85 (m, 6H), 1.74 (d, *J* = 2.7 Hz, 3H), 1.72 – 1.65 (m, 2H), 1.64 (s, 3H), 1.61 (s, 1H), 1.55 – 1.46 (m, 1H), 1.45 – 1.36 (m, 1H), 1.34 – 1.24 (m, 6H), 1.19 (s, 1H), 1.15 (m, 1H). ^{13}C NMR (126 MHz, $CDCl_3$) δ 170.1, 143.7, 138.3, 134.1, 133.8, 129.4 (two peaks overlapped), 127.6 (three peaks overlapped), 127.0, 120.4 (d, *J* = 14.8 Hz), 120.0, 86.4, 86.1, 52.80 (dd, *J* = 14.1, 11.8 Hz), 52.2 (three peaks overlapped), 52.1, 40.4 (d, *J* = 15.4 Hz), 40.1, 33.6, 33.4, 33.3, 33.2, 30.9, 26.3, 26.2, 26.1, 25.9, 25.7, 23.8, 23.6, 23.3, 22.3, 22.2 (two peaks overlapped), 22.1, 21.9, 21.6, 21.4, 21.3, 20.8, 20.6, 20.4 (two peaks overlapped). **HRMS**(m/z) calc. for $C_{24}H_{35}NNaO_4S$: $[M+Na]^+$, 456.2179; Found: 456.2180.

(3S,4aS,5aR,6aS,6bS,9R,9aR,11aS,11bR)-9a,11b-dimethyl-9-((R)-6-methylheptan-2-yl)-5-tosylhexadecahydro-2H-cyclopenta[1,2]phenanthro[8a,9-b]azirin-3-yl acetate (44a)[5]

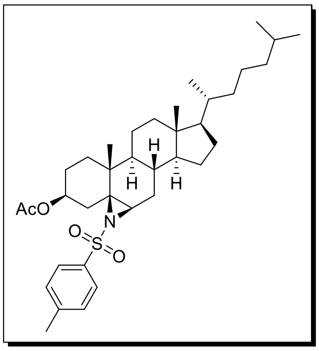

White solid. Melting point: 139.5 – 140.5 ºC. ^{1}H NMR (400 MHz, $CDCl_3$) δ 7.79 (d, *J* = 7.6 Hz, 2H), 7.29 (d, *J* = 7.9 Hz, 2H), 4.92 (m, 1H), 3.15 (s, 1H), 2.54 (m, 1H), 2.43 (s, 3H), 2.29 (dd, *J* = 13.4, 5.8 Hz, 1H), 2.04 (s, 3H), 1.93 (d, *J* = 12.6 Hz, 1H), 1.81 (m, 2H), 1.73 (m, 2H), 1.57 (s, 3H), 1.51 (m, 2H), 1.42 (m, 2H), 1.37 – 1.27 (m, 6H), 1.07 (m, 9H), 0.86 (m, 9H), 0.80 (m, 1H), 0.69 – 0.62 (m, 1H), 0.57 (s, 3H). ^{13}C NMR (100 MHz, $CDCl_3$) δ 170.6, 143.4, 139.1, 129.4, 126.9, 70.5, 57.1, 56.1, 56.0, 50.1, 48.3, 42.2, 39.7, 39.5, 36.2, 36.1, 35.7, 34.2, 32.6, 30.5, 29.9, 28.1, 28.0, 26.6, 24.1, 23.8, 22.8, 22.6, 22.1, 21.6, 21.3, 20.7, 18.6, 11.7. **HRMS**(m/z) calc. for $C_{36}H_{56}NO_4S$: $[M+H]^+$, 598.3925; Found: 598.3920.

(3S,6aS,6bS,9S,9aS,11aS,11bR)-9-acetyl-9a,11b-dimethyl-5-tosylhexadecahydro-2H-cyclopenta[1,2]phenanthro[8a,9-b]azirin-3-yl acetate (45a)

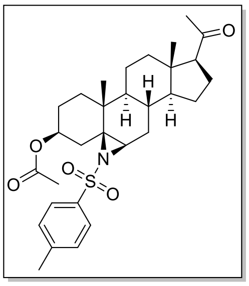

White solid. Melting point: 131.9 – 132.9 ºC. ^{1}H NMR (400 MHz, $CDCl_3$) δ 7.80 (d, *J* = 8.2 Hz, 2H), 7.31 (d, *J* = 8.1 Hz, 2H), 4.95 (m, 1H), 3.19 (s, 1H), 2.61 – 2.53 (m, 1H), 2.49 (m, 1H), 2.46 (s, 3H), 2.32 (m, 1H), 2.15 (m, 1H), 2.11 (s, 3H), 2.07 (s, 3H), 2.03 – 1.96 (m, 1H), 1.91 – 1.82 (m, 2H), 1.77 (m, 1H), 1.60 (m, 3H), 1.45 (m, 3H), 1.38 – 1.29 (m, 2H), 1.18 – 1.11 (m, 1H), 1.09 (s, 3H), 1.02 – 0.89 (m, 2H), 0.74 (m, 1H), 0.55 (s, 3H). ^{13}C NMR (100 MHz, $CDCl_3$) δ 209.2, 170.6, 143.6, 138.9, 129.4, 126.9, 70.4, 63.5, 56.9, 56.1, 49.9, 48.4, 43.8, 38.7, 36.3, 34.3, 32.6, 31.5, 30.4, 29.9, 26.6, 24.3, 22.7, 22.1, 21.6, 21.3, 20.7, 13.1. **HRMS**(m/z) calc. for $C_{30}H_{41}NNaO_5S$: $[M+Na]^+$, 550.2598; Found: 550.2595.

4-((1R,2R,4aS,8aS)-2-acetoxy-2,5,5,8a-tetramethyldecahydronaphthalen-1-yl)-2-(1-tosylaziridin-2-yl)butan-2-yl acetate (46a)

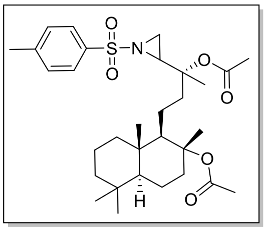

White solid. Melting point: 127.2 – 128.2 °C. ^{1}H NMR (400 MHz, $CDCl_3$) δ 7.84 (dd, *J* = 8.1, 4.1 Hz, 2H), 7.36 (dd, *J* = 8.0, 2.6 Hz, 2H), 3.25 (dd, *J* = 7.1, 4.6 Hz, 0.5H), 3.04 (dd, *J* = 7.0, 4.6 Hz, 0.5H), 2.68 – 2.57 (m, 2H), 2.46 (d, *J* = 6.5 Hz, 3H), 2.44 – 2.40 (m, 0.5H), 2.31 (d, *J* = 4.4 Hz, 0.5H), 1.97 (s, 1H), 1.96 – 1.92 (m, 3H), 1.89 (s, 1H), 1.81 (s, 1H), 1.55 (m, 6H), 1.45 (d, *J* = 5.2 Hz, 3H), 1.37 (m, 3H), 1.27 (d, *J* = 4.0 Hz, 3H), 1.21 (m, 3H), 1.12 (d, *J* = 13.0 Hz, 1H), 0.98 – 0.89 (m, 2H), 0.87 (d, *J* = 6.3 Hz, 3H), 0.81 (d, *J* = 2.6 Hz, 3H), 0.78 (s, 3H).^{13}C NMR (100 MHz, $CDCl_3$) δ 170.2 (d, *J* = 12.6 Hz), 169.7 (d, *J* = 4.3 Hz), 144.6, 134.4 (d, *J* = 17.6 Hz), 129.6 (d, *J* = 9.7 Hz), 128.3 (d, *J* = 8.5 Hz), 87.6 (d, *J* = 5.3 Hz), 81.4, 80.7, 58.7 (d, *J* = 22.9 Hz), 55.59 (d, *J* = 11.5 Hz), 44.5, 44.1, 41.8 (d, *J* = 7.4 Hz), 40.4, 39.3 (d, *J* = 10.0 Hz), 38.7 (d, *J* = 11.0 Hz), 37.4, 33.2 (d, *J* = 5.0 Hz), 33.0, 30.9, 30.5, 22.8 (d, *J* = 6.1 Hz), 21.8, 21.6 (t, *J* = 10.0 Hz), 21.3, 20.4, 20.3, 20.2, 19.8, 19.6, 19.1, 18.9, 18.2, 15.6. **HRMS**(m/z) calc. for $C_{31}H_{47}NNaO_6S$: $[M+Na]^+$, 584.3016; Found: 584.3019.

((1'S,2S,5'R,6'R,8a'R)-6'-acetoxy-1'-((E)-2-((S)-4-acetoxy-2-oxodihydrofuran-3(2H)-ylidene)ethyl)-5',8a'-dimethyl-1-tosyloctahydro-1'H-spiro[aziridine-2,2'-naphthalen]-5'-yl)methyl acetate (47a)

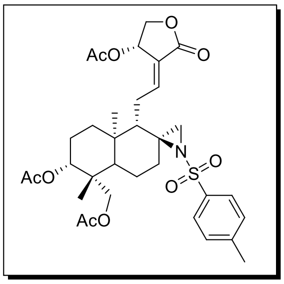

White solid. Melting point: 137.5 – 138.5 °C. ^{1}H NMR (400 MHz, $CDCl_3$) δ 7.79 (d, *J* = 8.3 Hz, 2H), 7.33 – 7.29 (m, 2H), 7.11 – 7.03 (m, 1H), 5.76 (dt, *J* = 5.8, 1.6 Hz, 1H), 4.55 (dd, *J* = 11.7, 4.4 Hz, 1H), 4.44 (dd, *J* = 11.1, 5.8 Hz, 1H), 4.33 (d, *J* = 11.8 Hz, 1H), 4.17 – 4.09 (m, 2H), 2.63 (s, 2H), 2.41 (s, 3H), 2.39 – 2.32 (m, 1H), 2.24 (m, 1H), 2.16 (m, 1H), 2.06 (s, 3H), 2.04 (s, 3H), 2.02 (m, 1H), 1.91 (m, 1H), 1.85 (s, 3H), 1.75 – 1.64 (m, 4H), 1.33 – 1.23 (m, 3H), 1.04 (s, 3H), 0.85 (s, 3H). ^{13}C NMR (100 MHz, $CDCl_3$) δ 171.1, 170. 6, 170.4, 169.3, 151.2, 144.2, 137.3, 129.7, 127.7, 122.3, 79.3, 71.4, 67.7, 64.8, 54.5, 54.4, 51.4, 41.0, 39.8, 37.1, 35.8, 32.8, 23.6, 23.6, 22.8, 22.7, 21.7, 21.2 (two peaks overlapped), 20.7, 14.5. **HRMS**(m/z) calc. for $C_{33}H_{43}NNaO_{10}S$:$[M+Na]^+$, 668.2500; found: 668.2501.

4-methyl-N,N-bis((2,2,6,6-tetramethylpiperidin-1-yl)oxy)benzenesulfonamide (48)

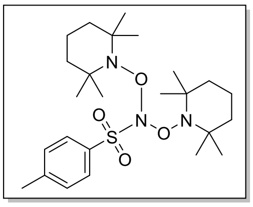

White solid. Melting point: 97.5 – 98.1 °C. ^{1}H NMR (500 MHz, $CDCl_3$) δ 7.94 (d, *J* = 8.3 Hz, 2H), 7.33 (d, *J* = 8.0 Hz, 2H), 2.44 (s, 3H), 1.82 – 1.77 (m, 2H), 1.55 (s, 6H), 1.47 – 1.41 (m, 5H), 1.29 (m, 3H), 1.21 – 1.15 (m, 2H), 1.13 (s, 6H), 1.06 (m, 12H). ^{13}C NMR (100 MHz, $CDCl_3$) δ 145.3, 139.1, 129.8, 129.3, 85.7, 78.2, 59.1, 43.1, 42.0, 40.7, 34.7, 29.6, 29.2, 26.7, 26.0, 21.7, 20.6, 18.4, 17.0. **HRMS**(m/z) calc. for $C_{25}H_{44}N_3O_4S$: $[M+H]^+$, 482.3047; Found: 482.3049.

trans-2,3-diphenyl-1-tosylaziridine (50)

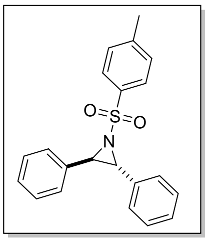

White solid. Melting point: 126.9 – 127.9 ºC. ^{1}H NMR (500 MHz, $CDCl_3$) δ 7.64 (d, *J* = 8.3 Hz, 2H), 7.45 – 7.40 (m, 4H), 7.38 – 7.34 (m, 6H), 7.21 (d, *J* = 8.0 Hz, 2H), 4.27 (s, 2H), 2.40 (s, 3H). ^{13}C NMR (126 MHz, $CDCl_3$) δ 144.0, 137.1, 133.1, 129.5, 128.7, 128.5, 128.3, 127.6, 50.4, 21.6. **HRMS**(m/z) calc. for $C_{21}H_{19}NNaO_2S$: $[M+Na]^+$, 372.1029; Found: 372.1029.

General procedure for copper-catalyzed allylic C-H amidation of alkenes: In a glovebox, a solution of $[Cu(OTf)]_2$·toluene (10 mol%) and **L_9** (15 mol%) in DCE (0.9 mL) was added into a 2.0 mL vial. The mixture was stirred for 30 min. Then 4ÅMS (100 mg), alkene (0.5 mmol of simple alkenes or 0.2 mmol of complex natural products) and **1a** (0.1 mmol) were added successively. The resulting mixture was stirred at room temperature for 16 h. The resultant reaction mixture was purified by preparative TLC (hexane/EA 5:1).

N-(cyclohex-2-en-1-yl)-4-methylbenzenesulfonamide (4)

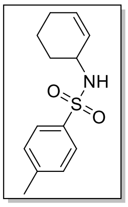

White solid. Melting point: 58.5 – 59.5 ºC. ^{1}H NMR (300 MHz, $CDCl_3$) δ 7.77 (d, *J* = 8.3 Hz, 2H), 7.30 (d, *J* = 8.1 Hz, 2H), 5.76 (m, 1H), 5.41 – 5.26 (m, 1H), 4.52 (d, *J* = 8.5 Hz, 1H), 3.81 (dd, *J* = 5.1, 3.1 Hz, 1H), 2.43 (s, 3H), 2.01 – 1.84 (m, 2H), 1.82 – 1.65 (m, 2H), 1.63 – 1.57 (m, 1H), 1.55 – 1.49 (m, 1H). ^{13}C NMR (75 MHz, $CDCl_3$) δ 143.3, 138.3, 131.6, 129.7, 127.0 (two peaks overlapped), 49.0, 30.3, 24.5, 21.6, 19.3. **HRMS**(m/z) calc. for $C_{13}H_{17}NNaO_2S$: $[M+Na]^+$, 274.0872; Found: 274.0871.

N-(cyclopent-2-en-1-yl)-4-methylbenzenesulfonamide (28)

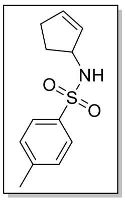

Colorless oil. ^{1}H NMR (300 MHz, $CDCl_3$) δ 7.77 (d, *J* = 8.3 Hz, 2H), 7.31 (d, *J* = 8.0 Hz, 2H), 5.92 – 5.81 (m, 1H), 5.48 – 5.38 (m, 1H), 4.42 (m, 2H), 2.44 (s, 3H), 2.39 – 2.28 (m, 1H), 2.26 – 2.08 (m, 2H), 1.54 – 1.44 (m, 1H). ^{13}C NMR (75 MHz, $CDCl_3$) δ 143.4, 138.1, 135.1, 130.5, 129.7, 127.1, 59.8, 31.6, 30.8, 21.6. **HRMS**(m/z) calc. for $C_{12}H_{15}NNaO_2S$: $[M+Na]^+$, 260.0716; Found: 260.0715.

(_E_)-N-(1,3-diphenylallyl)-4-methylbenzenesulfonamide (30)

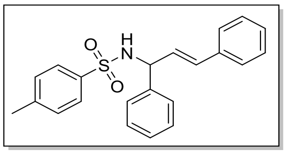

White solid. Melting point: 130.2 – 131.2 ºC. ^{1}H NMR (400 MHz, $CDCl_3$) δ 7.70 (d, *J* = 8.0 Hz, 2H), 7.24 (m, 13H), 6.39 (d, *J* = 15.8 Hz, 1H), 6.18 – 6.05 (m, 1H), 5.15 (m, 2H), 2.36 (s, 3H). ^{13}C NMR (100 MHz, $CDCl_3$) δ 143.3, 139.7, 137.7, 136.1, 132.2, 129.5, 128.8, 128.5, 128.2, 127.9 (two peaks overlapped), 127.4, 127.1, 126.6, 59.8, 21.5. **HRMS**(m/z) calc. for $C_{22}H_{21}NNaO_2S$: $[M+Na]^+$, 386.1185; Found: 386.1180.

(_E_)-N-(1,3-bis(4-chlorophenyl)allyl)-4-methylbenzenesulfonamide (31)

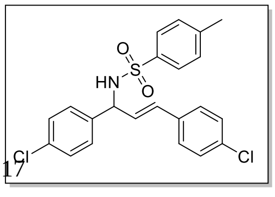

White solid. Melting point: 138.3 – 139.3 ºC. ^{1}H NMR (400 MHz, $CDCl_3$) δ 7.62 (d, *J* = 8.2 Hz, 2H), 7.24 – 7.16 (m, 4H), 7.16 – 7.07 (m, 6H), 6.27 (d, *J* = 15.8 Hz, 1H), 6.02 (dd, *J* = 15.8, 6.7 Hz, 1H), 5.39 (d, *J* = 7.4 Hz,

1H), 5.06 (t, J = 7.0 Hz, 1H), 2.34 (s, 3H). ^{13}C NMR (100 MHz, $CDCl_3$) δ 143.6, 137.9, 137.5, 134.4, 133.8 (two peaks overlapped), 131.3, 129.5, 128.9, 128.7, 128.5, 128.3, 127.8, 127.3, 59.1, 21.49 (s). **HRMS**(m/z) calc. for $C_{22}H_{19}Cl_2NNaO_2S$: $[M+Na]^+$, 454.0406; Found: 454.0407.

(*E*)-4-methyl-N-(pent-3-en-2-yl)benzenesulfonamide (32)[6]

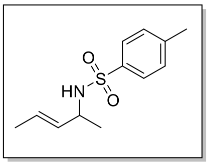

Colorless oil. ^{1}H NMR (400 MHz, $CDCl_3$) δ 7.73 (d, J = 8.3 Hz, 2H), 7.28 (d, J = 8.1 Hz, 2H), 5.43 (dqd, J = 12.9, 6.4, 1.0 Hz, 1H), 5.17 (ddd, J = 15.3, 6.6, 1.6 Hz, 1H), 4.45 (d, J = 7.3 Hz, 1H), 3.84 (h, J = 6.6 Hz, 1H), 2.42 (s, 3H), 1.52 (d, J = 6.5 Hz, 3H), 1.15 (d, J = 6.7 Hz, 3H). ^{13}C NMR (100 MHz, $CDCl_3$) δ 143.2, 138.2, 131.9, 129.5, 127.3, 126.7, 51.5, 22.0, 21.6, 17.5. **HRMS**(m/z) calc. for $C_{12}H_{17}NNaO_2S$: $[M+Na]^+$, 262.0872; Found: 262.0873.

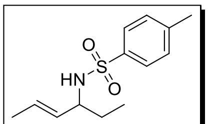

33a

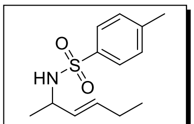

33b

Colorless oil. The product was obtained as a mixture of isomers (**33a**/**33b** 2:1). The HNMR and CNMR spectra of each isomer was consistent with the literature data[7]. ^{1}H NMR (400 MHz, $CDCl_3$) δ 7.76 (m, 2H, from I and II), 7.32 – 7.26 (m, 2H, from I and II), 5.59 – 5.47 (m, 0.26H, from II), 5.46 – 5.29 (m, 0.69H, from I), 5.14 (m, 0.29H, from II), 5.00 (m, 1H), 4.55 – 4.37 (m, 0.83H, from I and II), 3.86 (m, 0.29H, from II), 3.64 – 3.48 (m, 0.60H, from I), 2.43 (s, 1H, from II), 2.41 (s, 2H, from I), 1.92 – 1.84 (m, 0.94H, from II), 1.53 – 1.39 (m, 1.98H, from I), 1.34 – 1.26 (m, 1.09H, from I), 1.16 (d, J = 6.7 Hz, 1.05H, from II), 0.82 (m, 3H, from I and II). ^{13}C NMR (100 MHz, $CDCl_3$) δ 143.1 (m), 138.2 (m), 135.0 – 130.3 (m), 129.6 (m), 127.7 (s), 127.5 – 126.5 (m,), 124.5 (s), 57.7 (s), 51.5 (s), 45.4 (s), 34.2 (s), 29.7 (s), 28.9 (s), 25.0 (s), 22.1 (two peaks overlapped), 21.5 (s), 17.6 (s), 13.6 (s), 13.1 (s), 9.9 (s). **HRMS**(m/z) calc. for $C_{13}H_{19}NNaO_2S$: $[M+Na]^+$, 276.1029; Found: 276.1028.

4-methyl-N-(3-methylcyclohex-2-en-1-yl)benzenesulfonamide (34a)

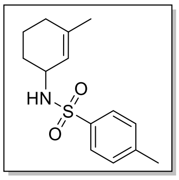

White solid. Melting point: 59.1 – 59.2 ºC. ^{1}H NMR (500 MHz, $CDCl_3$) δ 7.81 – 7.74 (m, 2H), 7.31 (d, J = 8.0 Hz, 2H), 5.13 – 5.04 (m, 1H), 4.48 (d, J = 8.2 Hz, 1H), 3.78 (d, J = 1.7 Hz, 1H), 2.44 (s, 3H), 1.90 – 1.77 (m, 2H), 1.72 – 1.64 (m, 1H), 1.63 – 1.60 (m, 1H), 1.58 (d, J = 6.5 Hz, 3H), 1.56 – 1.46 (m, 2H). ^{13}C NMR (101 MHz, $CDCl_3$) δ 143.1, 139.5, 138.3, 129.6, 126.9, 121.2, 49.4, 29.9, 29.4, 23.5, 21.4, 19.3. **HRMS**(m/z) calc. for $C_{14}H_{19}NNaO_2S$: $[M+Na]^+$, 288.1029; Found: 288.1028.

4-methyl-N-(2-methylcyclohex-2-en-1-yl)benzenesulfonamide (34b)

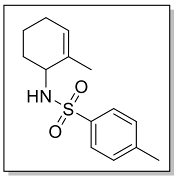

White solid. Melting point: 66.5 – 67.5 ºC. ^{1}H NMR (500 MHz, $CDCl_3$) δ 7.78 (d, J = 8.1 Hz, 2H), 7.31 (d, J = 8.0 Hz, 2H), 5.53 (s, 1H), 4.40 (d, J = 8.4 Hz, 1H), 3.61 (m, 1H), 2.44 (s, 3H), 1.99 – 1.85 (m, 2H), 1.70 – 1.65 (m, 1H), 1.60 – 1.48 (m, 3H), 1.45 (s, 3H). ^{13}C NMR (126 MHz, $CDCl_3$) δ 143.2, 132.2, 129.7, 127.3, 127.1, 108.5, 52.1, 30.3, 24.9, 21.6, 20.7, 17.7. **HRMS**(m/z) calc. for $C_{14}H_{19}NNaO_2S$: $[M+Na]^+$, 288.1029; Found: 288.1029.

4-methyl-N-(3,4,5,6-tetrahydro-[1,1'-biphenyl]-3-yl)benzenesulfonamide (35a)

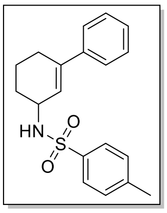

White solid. Melting point: 119.5 – 120.5 ºC. ^{1}H NMR (500 MHz, $CDCl_3$) δ 7.82 (d, *J* = 8.2 Hz, 2H), 7.33 (d, *J* = 8.1 Hz, 2H), 7.26 (tt, *J* = 6.4, 5.0 Hz, 5H), 5.77 – 5.64 (m, 1H), 4.70 (d, *J* = 8.5 Hz, 1H), 4.02 (dd, *J* = 6.4, 2.1 Hz, 1H), 2.44 (d, *J* = 8.4 Hz, 3H), 2.41 – 2.29 (m, 2H), 1.81 (m, 2H), 1.73 – 1.67 (m, 1H), 1.63 – 1.53 (m, 1H). ^{13}C NMR (100 MHz, $CDCl_3$) δ 143.3, 140.9, 140.8, 138.3, 129.7, 128.2, 127.5, 127.0, 125.2, 123.7, 49.8, 29.8, 26.9, 21.5, 19.8. **HRMS**(m/z) calc. for $C_{19}H_{21}NNaO_2S$: $[M+Na]^+$, 350.1185; Found: 350.1186.

4-methyl-N-(2,3,4,5-tetrahydro-[1,1'-biphenyl]-2-yl)benzenesulfonamide (35b)

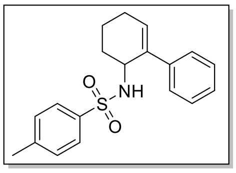

White solid. Melting point: 141.1 – 142.1 ºC. ^{1}H NMR (500 MHz, $CDCl_3$) δ 7.53 (d, *J* = 8.3 Hz, 2H), 7.16 (t, *J* = 8.6 Hz, 3H), 7.06 (t, *J* = 7.7 Hz, 2H), 6.93 (dd, *J* = 8.2, 1.1 Hz, 2H), 6.09 (dd, *J* = 4.6, 3.2 Hz, 1H), 4.33 (d, *J* = 5.8 Hz, 1H), 4.24 (d, *J* = 3.5 Hz, 1H), 2.44 (s, 3H), 2.25 – 2.08 (m, 3H), 1.77 – 1.65 (m, 3H). ^{13}C NMR (100 MHz, $CDCl_3$) δ 142.9, 139.1, 137.0, 136.3, 131.2, 129.4, 128.2, 127.0, 126.9, 126.0, 49.4, 29.8, 25.6, 21.5, 16.5. **HRMS**(m/z) calc. for $C_{19}H_{21}NNaO_2S$: $[M+Na]^+$, 350.1185; Found: 350.1186.

2-(4-methyl-5-((4-methylphenyl)sulfonamido)cyclohex-3-en-1-yl)propan-2-yl acetate (36c)

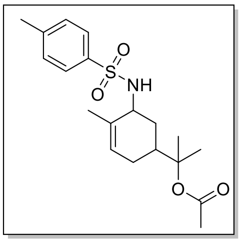

White solid. Melting point: 97.2 – 98.2 ºC. ^{1}H NMR (400 MHz, $CDCl_3$) δ 7.79 (d, *J* = 8.3 Hz, 2H), 7.31 (d, *J* = 8.0 Hz, 2H), 5.53 (d, *J* = 4.9 Hz, 1H), 4.49 (d, *J* = 8.3 Hz, 1H), 3.71 (m, 1H), 2.43 (s, 3H), 2.04 (m, 2H), 1.93 (s, 3H), 1.83 – 1.73 (m, 2H), 1.51 (s, 3H), 1.31 (m, 6H), 1.30 – 1.25 (m, 1H). ^{13}C NMR (100 MHz, $CDCl_3$) δ 170.4, 143.2, 138.3, 131.7, 129.6, 127.0, 126.3, 83.8, 52.5, 36.9, 30.5, 26.1, 23.2, 22.3, 21.4, 20.2. **HRMS**(m/z) calc. for $C_{19}H_{27}NNaO_4S$: $[M+Na]^+$, 388.1553; Found: 388.1553.

4-methyl-N-(3-methyl-6-(prop-1-en-2-yl)cyclohex-2-en-1-yl)benzenesulfonamide (37c)[8]

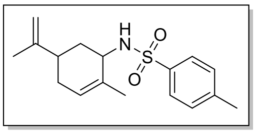

White solid. Melting point: 95.9 – 96.9 ºC. ^{1}H NMR (400 MHz, $CDCl_3$) δ 7.79 (d, *J* = 8.3 Hz, 2H), 7.30 (d, *J* = 8.0 Hz, 2H), 5.54 (s, 1H), 4.70 (s, 1H), 4.63 (s, 1H), 4.58 (d, *J* = 8.0 Hz, 1H), 3.72 – 3.61 (m, 1H), 2.43 (s, 3H), 2.15 – 2.04 (m, 2H), 1.89 – 1.75 (m, 2H), 1.63 (s, 3H), 1.53 (m, 1H), 1.44 (s, 3H). ^{13}C NMR (100 MHz, $CDCl_3$) δ 148.2, 143.2, 138.1, 131.3, 129.6, 129.5, 127.0, 126.9, 109.2, 52.5, 35.1, 34.6, 30.3, 21.4, 20.7, 20.3. **HRMS**(m/z) calc. for $C_{17}H_{23}NNaO_2S$: $[M+Na]^+$, 328.1342; Found: 328.1342.

rac-4-methyl-N-((1R,2S,5S)-4,6,6-trimethylbicyclo[3.1.1]hept-3-en-2-yl)benzenesulfonamide (38b)

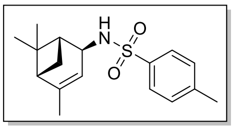

White solid. Melting point: 63.2 – 64.2 ºC. ^{1}H NMR (500 MHz, $CDCl_3$) δ 7.77 (d, *J* = 8.3 Hz, 2H), 7.31 (d, *J* = 8.0 Hz, 2H), 4.91 (d, *J* = 1.5 Hz, 1H), 4.62 (d, *J* = 8.9 Hz, 1H), 3.90 (dd, *J* = 8.9, 1.7 Hz, 1H), 2.44 (s, 3H), 2.23 (dt, *J* = 9.4, 5.5 Hz, 1H), 2.13 – 2.07 (m, 1H), 1.99 – 1.94 (m, 1H), 1.63 (s, 3H), 1.25 (s, 3H), 1.13 (d, *J* = 9.4 Hz, 1H), 0.82 (s, 3H). ^{13}C NMR (100 MHz, $CDCl_3$) δ 149.4, 143.0, 138.5, 129.6, 126.8, 115.9, 54.0, 46.9, 46.1, 44.2, 28.5, 26.2, 22.6, 21.5, 20.3. **HRMS**(m/z) calc. for $C_{17}H_{23}NNaO_2S$: $[M+Na]^+$, 328.1342; Found: 328.1342.

(E)-4-methyl-N-(2,4,4-trimethyl-3-(3-oxobut-1-en-1-yl)cyclohex-2-en-1-yl)benzenesulfonamide (42c)

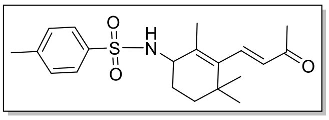

White solid. Melting point: 138.9 – 139.9 °C. ^{1}H NMR (400 MHz, $CDCl_3$) δ 7.79 (d, *J* = 8.3 Hz, 2H), 7.32 (d, *J* = 8.0 Hz, 2H), 7.09 (d, *J* = 16.4 Hz, 1H), 6.03 (d, *J* = 16.4 Hz, 1H), 4.59 (d, *J* = 8.7 Hz, 1H), 3.66 (m, 1H), 2.44 (s, 3H), 2.28 (s, 3H), 1.75 – 1.66 (m, 1H), 1.58 (m, 1H), 1.54 (s, 3H), 1.50 (m, 1H), 1.40 (m, 1H), 1.00 (m, 6H). ^{13}C NMR (100 MHz, $CDCl_3$) δ 198.1, 143.4, 142.1, 141.1, 138.0, 133.3, 130.3, 129.6, 127.0, 126.4, 53.7, 34.2, 34.1, 29.1, 27.4, 27.0, 26.1, 21.5, 18.7. **HRMS**(m/z) calc. for $C_{20}H_{27}NNaO_3S$: $[M+Na]^+$, 384.1604; Found: 384.1604.

6-methyl-2-(4-methyl-2-((4-methylphenyl)sulfonamido)cyclohex-3-en-1-yl)hept-5-en-2-yl acetate (43c)

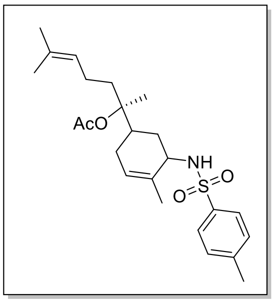

White solid. Melting point: 115.8 – 116.8 °C. ^{1}H NMR (400 MHz, $CDCl_3$) δ 7.79 (dd, *J* = 8.3, 3.5 Hz, 2H), 7.30 (dd, *J* = 8.0, 3.3 Hz, 2H), 5.52 (m, 1H), 5.06 (m, 1H), 4.54 (dd, *J* = 8.4, 5.5 Hz, 1H), 3.69 (m, 1H), 2.43 (s, 3H), 2.38 – 2.27 (m, 1H), 1.94 (t, *J* = 7.7 Hz, 6H), 1.71 (m, 1H), 1.68 (m, 1H), 1.61 (m, 8H), 1.49 (s, 3H), 1.27 (m, 3H), 1.24 (m, 1H). ^{13}C NMR (100 MHz, $CDCl_3$) δ 170.3, 143.1, 138.3, 131.9, 131.8, 131.7, 129.6, 129.5, 127.0 (two peaks overlapped), 126.5, 126.1, 123.8, 123.7, 86.1, 85.9, 52.5, 52.4, 35.5, 35.4, 35.0, 34.9, 30.8, 30.5, 26.2, 25.9, 25.7, 25.6, 22.1 (two peaks overlapped), 21.9, 21.4, 20.6, 20.5, 20.2, 17.5. **HRMS**(m/z) calc. for $C_{24}H_{35}NNaO_4S$: $[M+Na]^+$, 456.2179; Found: 456.2179.

N-((8S,9S,10R,13R,14S,17R)-10,13-dimethyl-17-((R)-6-methylheptan-2-yl)-2,7,8,9,10,11,12,13,14,15,16,17-dodecahydro-1H-cyclopenta[a]phenanthren-7-yl)-4-methylbenzenesulfonamide (44c)

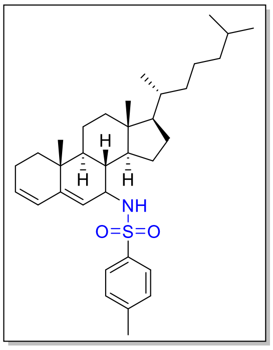

White solid. Melting point: 60.1 – 61.1 °C. ^{1}H NMR (400 MHz, $CDCl_3$) δ 7.76 (d, *J* = 8.1 Hz, 2H), 7.30 (d, *J* = 8.0 Hz, 2H), 5.77 (dd, *J* = 9.9, 2.6 Hz, 1H), 5.64 (dd, *J* = 9.9, 1.9 Hz, 1H), 5.01 (d, *J* = 4.9 Hz, 1H), 4.49 (d, *J* = 7.9 Hz, 1H), 3.82 (dt, *J* = 8.8, 5.0 Hz, 1H), 2.43 (s, 3H), 2.04 – 1.98 (m, 2H), 1.87 – 1.78 (m, 2H), 1.76 – 1.69 (m, 1H), 1.50 (m, 3H), 1.38 – 1.27 (m, 6H), 1.12 (m, 8H), 0.98 (m, 1H), 0.91 (m, 3H), 0.88 – 0.84 (m, 10H), 0.71 (s, 3H). ^{13}C NMR (100 MHz, $CDCl_3$) δ 147.2, 143.2, 138.3, 132.8, 129.7, 127.7, 127.0, 120.2, 56.0, 54.1, 51.2, 47.9, 43.2, 39.8, 39.5, 37.2, 36.2, 35.8, 34.8, 29.5, 28.2, 28.0, 25.9, 23.9, 23.7, 22.8, 22.6, 21.6, 20.9, 18.7, 17.0, 12.0. **HRMS**(m/z) calc. for $C_{34}H_{52}NO_2S$: $[M+H]^+$, 538.3713; Found: 538.3714.

N-((8S,9S,10R,13S,14S,17S)-17-acetyl-10,13-dimethyl-2,7,8,9,10,11,12,13,14,15,16,17-dodecahydro-1H-cyclopenta[a]phenanthren-7-yl)-4-methylbenzenesulfonamide (45c)[9]

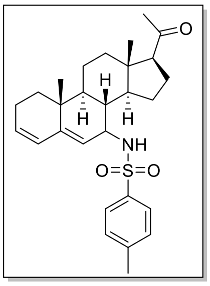

White solid. Melting point: 77.2 – 78.2 °C. ^{1}H NMR (400 MHz, $CDCl_3$) δ 7.77 (d, *J* = 8.3 Hz, 2H), 7.32 (d, *J* = 8.1 Hz, 2H), 5.80 (dd, *J* = 9.9, 2.6 Hz, 1H), 5.62 (d, *J* = 11.0 Hz, 1H), 5.05 (d, *J* = 4.9 Hz, 1H), 4.51 (d, *J* = 7.9 Hz, 1H), 3.87 – 3.78 (m, 1H), 2.55 (t, *J* = 9.2 Hz, 1H), 2.44 (s, 3H), 2.26 – 2.18 (m, 1H), 2.13 (s, 3H), 2.09 – 2.02 (m, 2H), 1.89 – 1.79 (m, 3H), 1.75 – 1.57 (m, 4H), 1.53 (m, 1H), 1.49 – 1.19 (m, 7H), 1.07 – 0.98 (m, 1H), 0.86 (s, 3H), 0.67 (s, 3H). ^{13}C NMR (125 MHz, $CDCl_3$) δ 209.3, 146.9, 143.3, 138.2, 131.9, 129.7, 128.2, 127.1,

120.8, 63.4, 54.4, 51.2, 47.8, 44.7, 38.9, 37.2, 34.8, 31.5, 29.6, 25.9, 23.9, 22.9, 21.6, 20.9, 17.0, 13.4. **HRMS**(m/z) calc. for $C_{28}H_{37}NNaO_3S$: $[M+Na]^+$, 490.2388; Found: 490.2386.

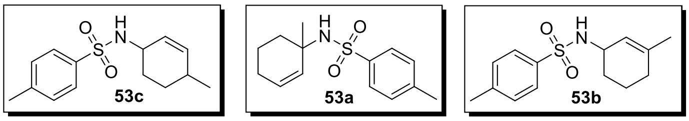

White solid. The product was obtained as a mixture of isomers (**53c**/**53a**/**53b** 1:2:9). ^1H NMR (400 MHz, $CDCl_3$) δ 7.84 – 7.73 (m, 2H, from **53c**, **53a** and **53b**), 7.32 – 7.24 (m, 2H, from **53c**, **53a** and **53b**), 5.65 (m, 0.22H, from **53a**), 5.56 (m, 0.07H, from **53c**), 5.49 (m, 0.14H, from **53a**), 5.08 (m, 0.53H, from **53b**), 4.64 (s, 0.14H, from **53a**), 4.54 – 4.44 (m, 0.61H, from **53c** and **53b**), 3.77 (m, 0.61H, from **53c** and **53b**), 2.42 (m, 3H, from **53c**, **53a** and **53b**), 2.00 – 1.92 (m, 0.47H), 1.92 – 1.73 (m, 2.17H), 1.72 – 1.43 (m, 5.09H, from **53c**, **53a** and **53b**), 1.42 – 1.29 (m, 0.27H), 1.25 (m, 0.50H), 1.14 (m, 0.10H), 0.94 – 0.87 (m, 0.59H). **HRMS**(m/z) calc. for $C_{14}H_{19}NNaO_2S$: $[M+Na]^+$, 288.1029; Found: 288.1028.

Supplementary Note2

^1H NMR and ^{13}C NMR Copies of Products:

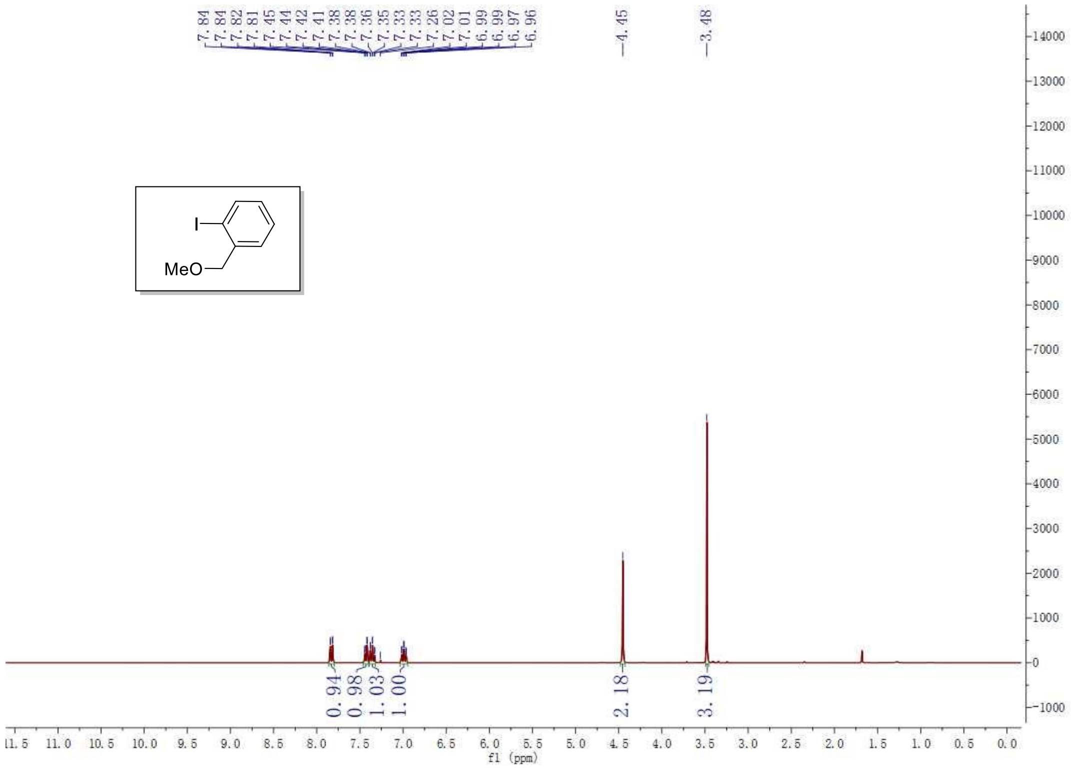

Supplementary Figure 1. ^1H NMR spectra for compound 1-iodo-2-(methoxymethyl)benzene

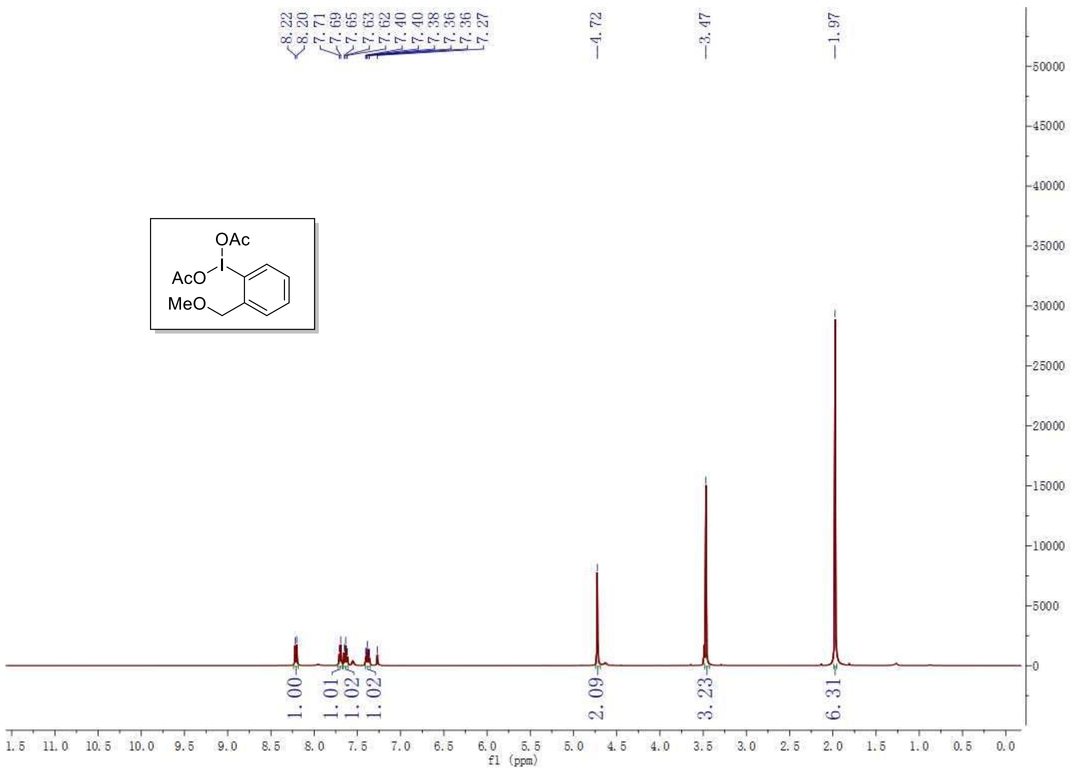

Supplementary Figure 2. ^{1}H NMR spectra for compound (2-(methoxymethyl)phenyl)-λ^{3}-iodanediyl diacetate

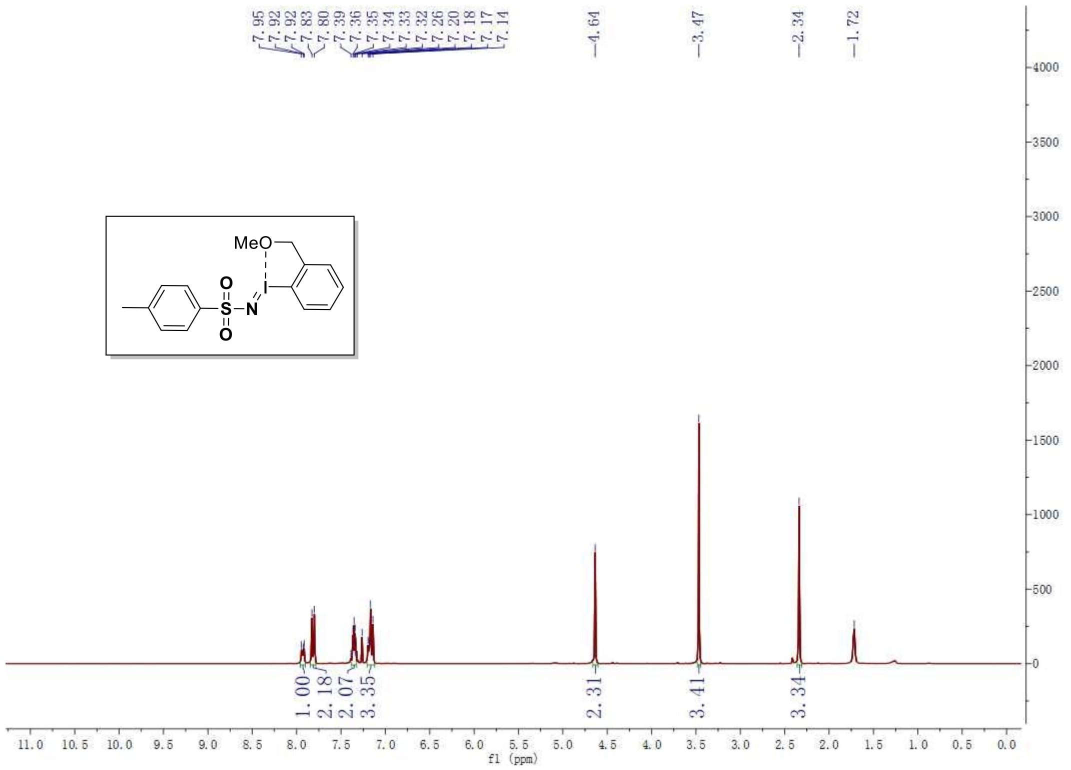

Supplementary Figure 3. ^{1}H NMR spectra for N-((2-(methoxymethyl)phenyl)- λ^{3}-iodanylidene)-4-methylbenzenesulfonamide

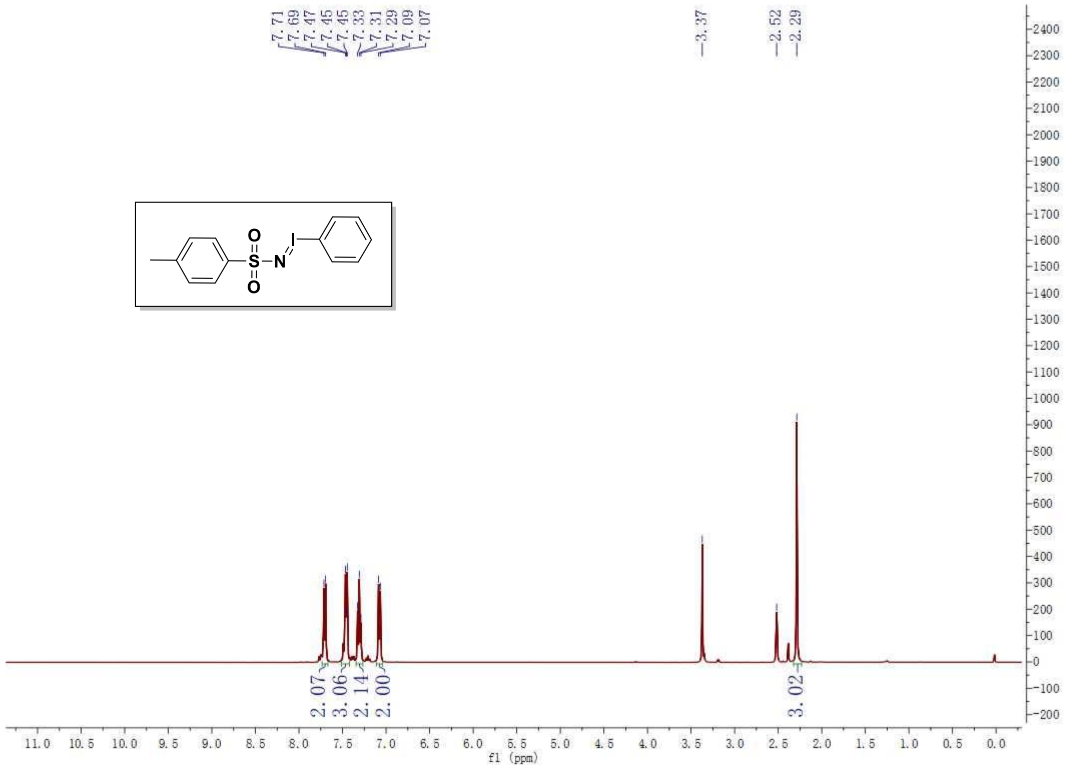

Supplementary Figure 4. ¹H NMR spectra for 4-methyl-N-(phenyl-λ³-iodanylidene)benzenesulfonamide

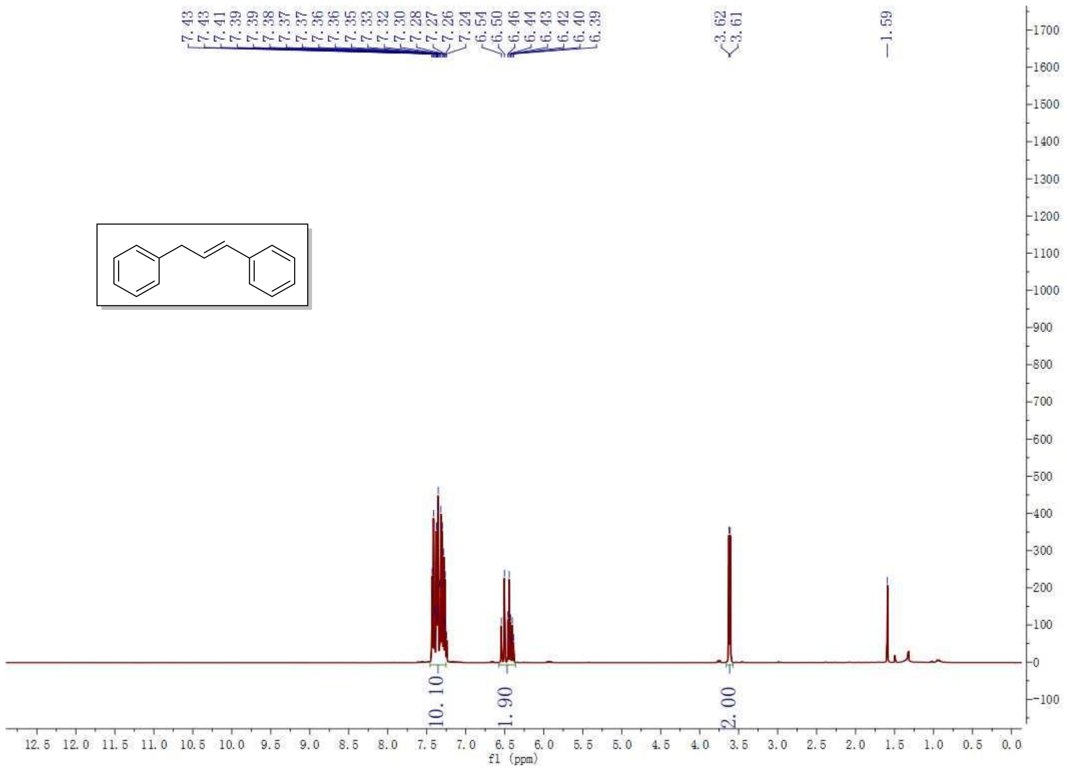

Supplementary Figure 5. ¹H NMR spectra for (*E*)-prop-1-ene-1,3-diyldibenzene

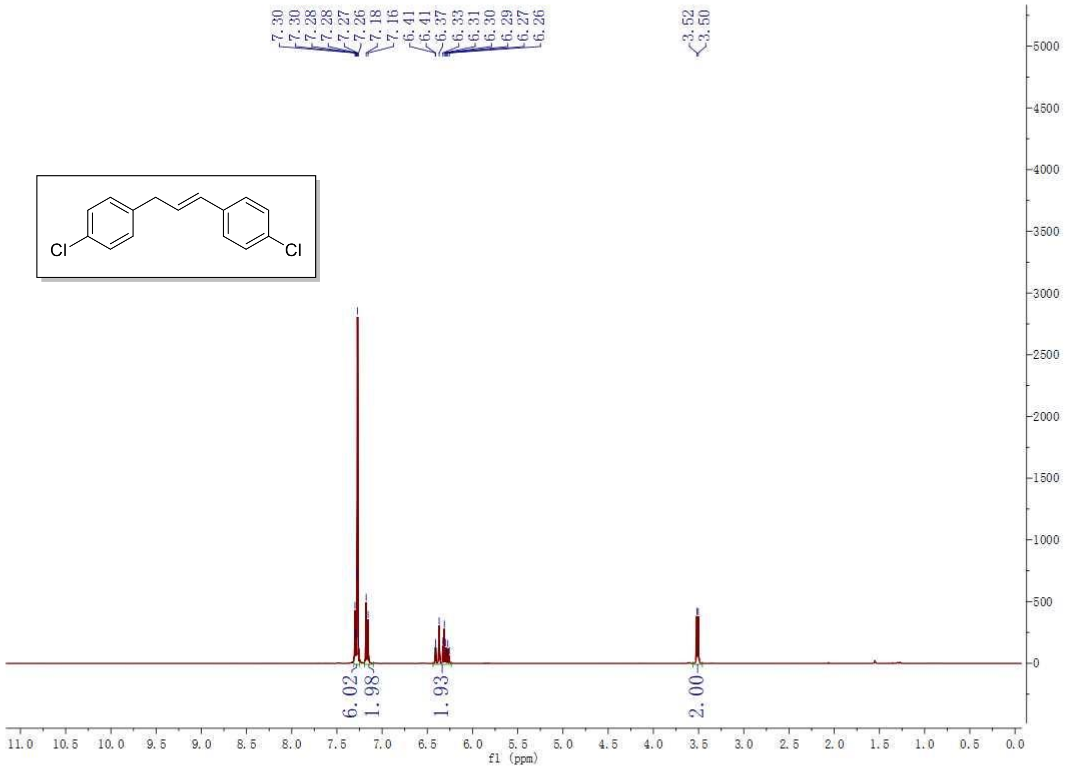

Supplementary Figure 6. **^{1}H NMR spectra for (*E*)-4,4'-(prop-1-ene-1,3-diyl)bis(chlorobenzene)**

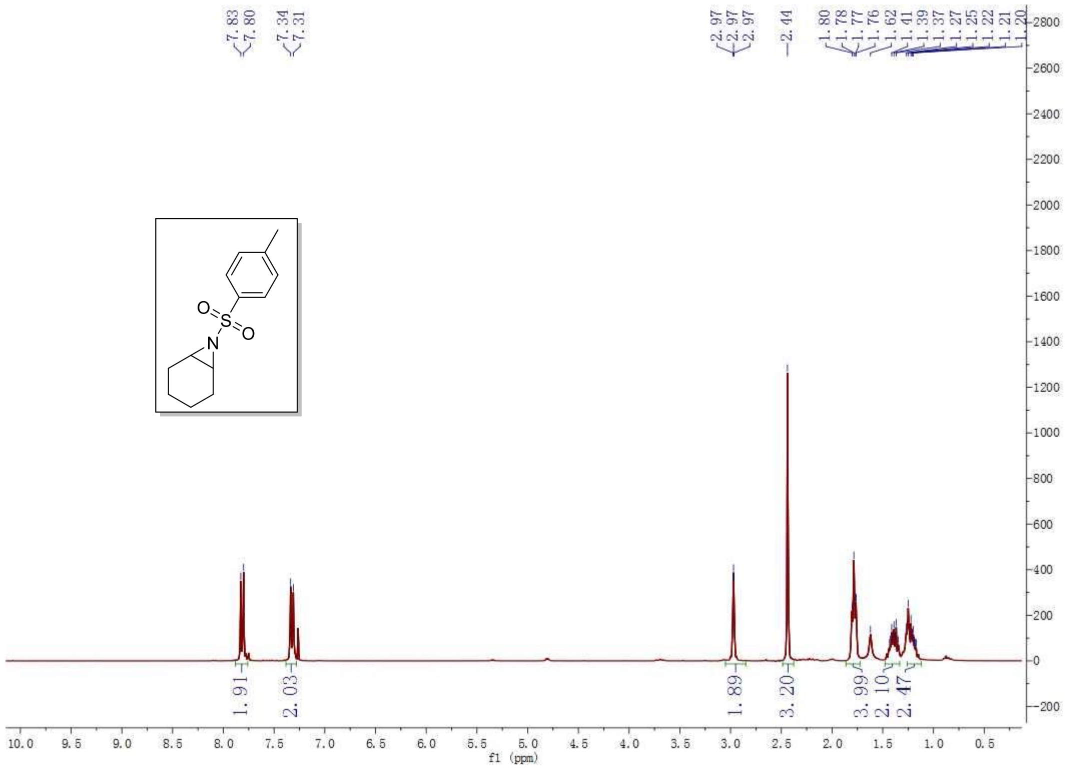

Supplementary Figure 7. ^{1}**H NMR spectra for compound 3**

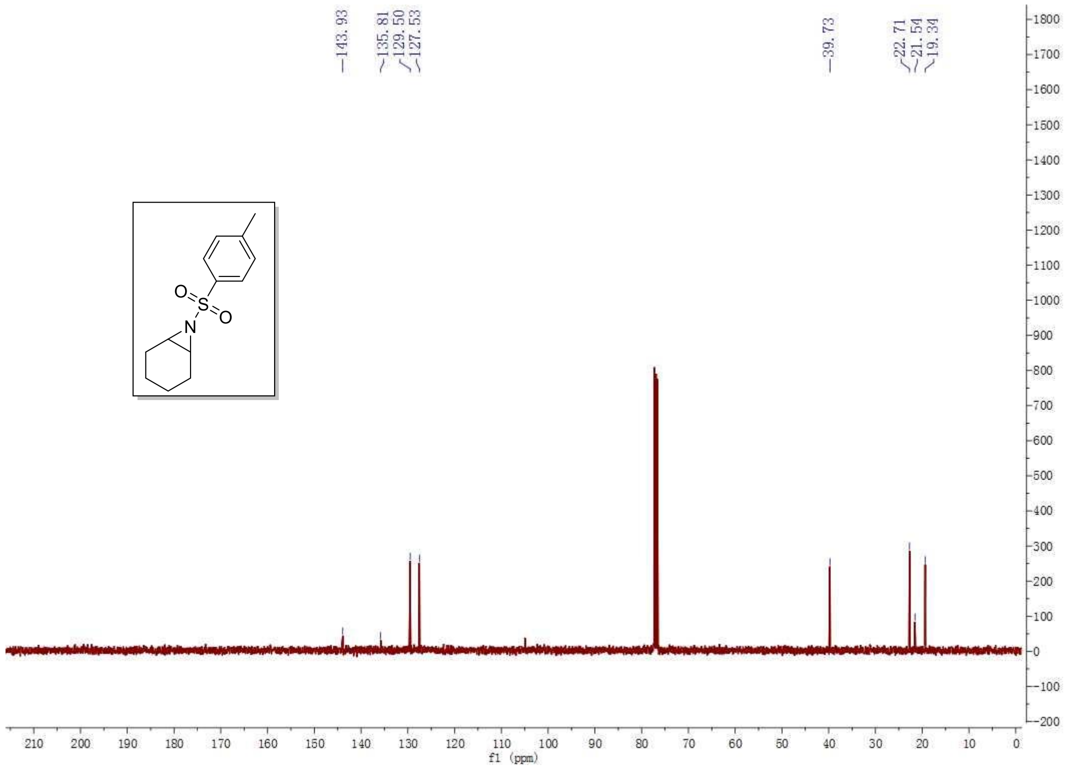

Supplementary Figure 8. ^{13}**C NMR spectra for compound 3**

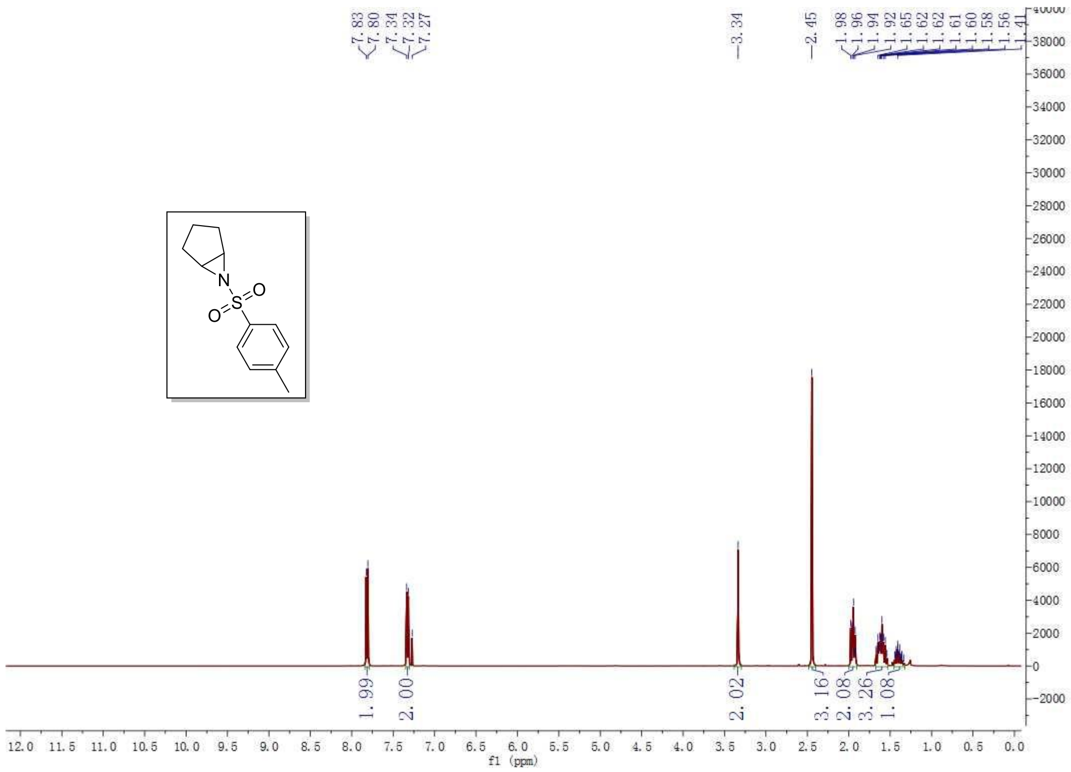

Supplementary Figure 9. ^{1}H NMR spectra for compound 8

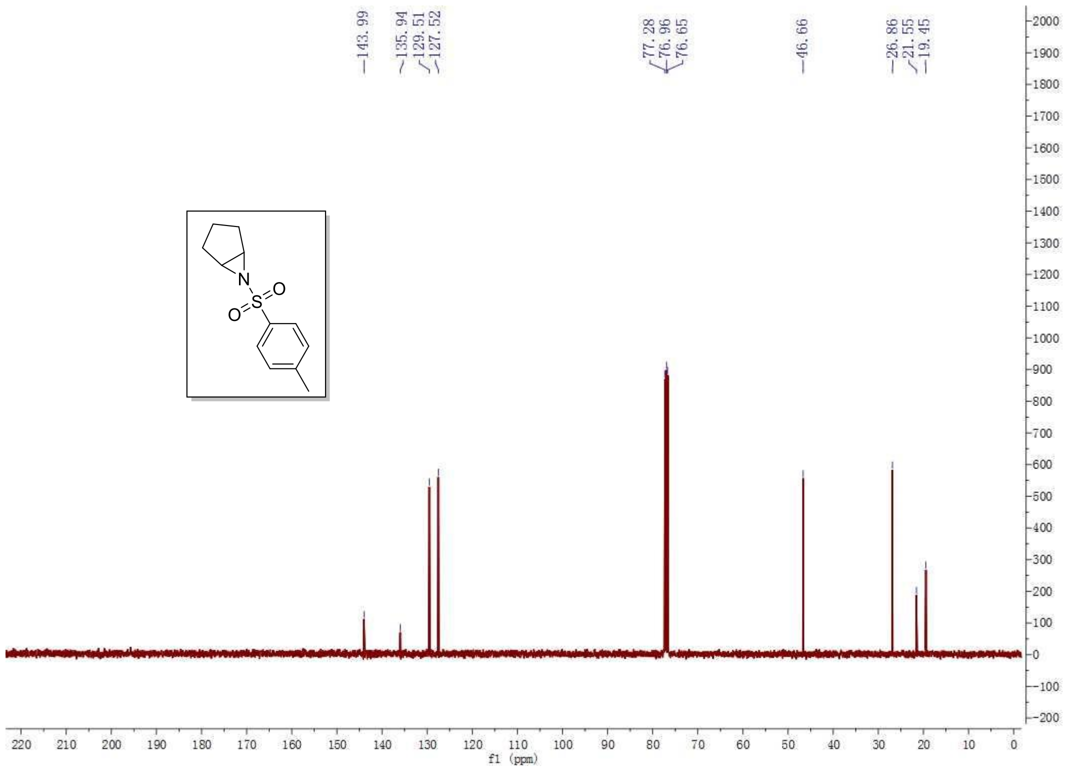

Supplementary Figure 10. ^{13}C NMR spectra for compound 8

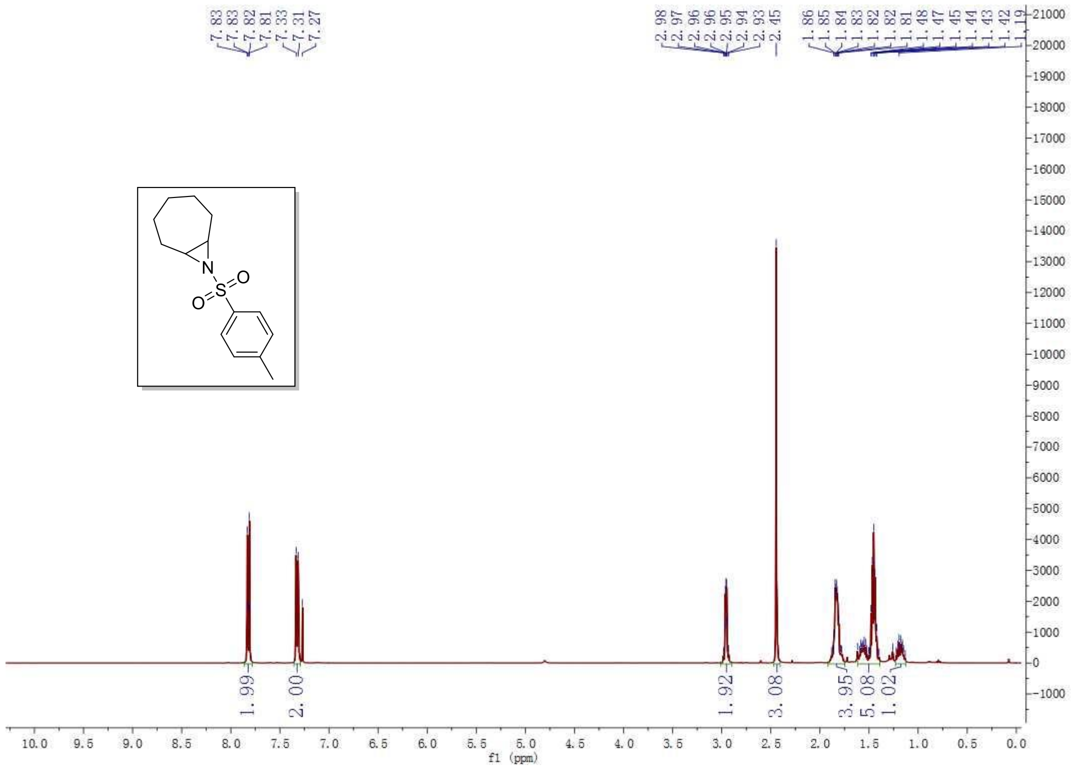

Supplementary Figure 11. ^{1}H NMR spectra for compound 9

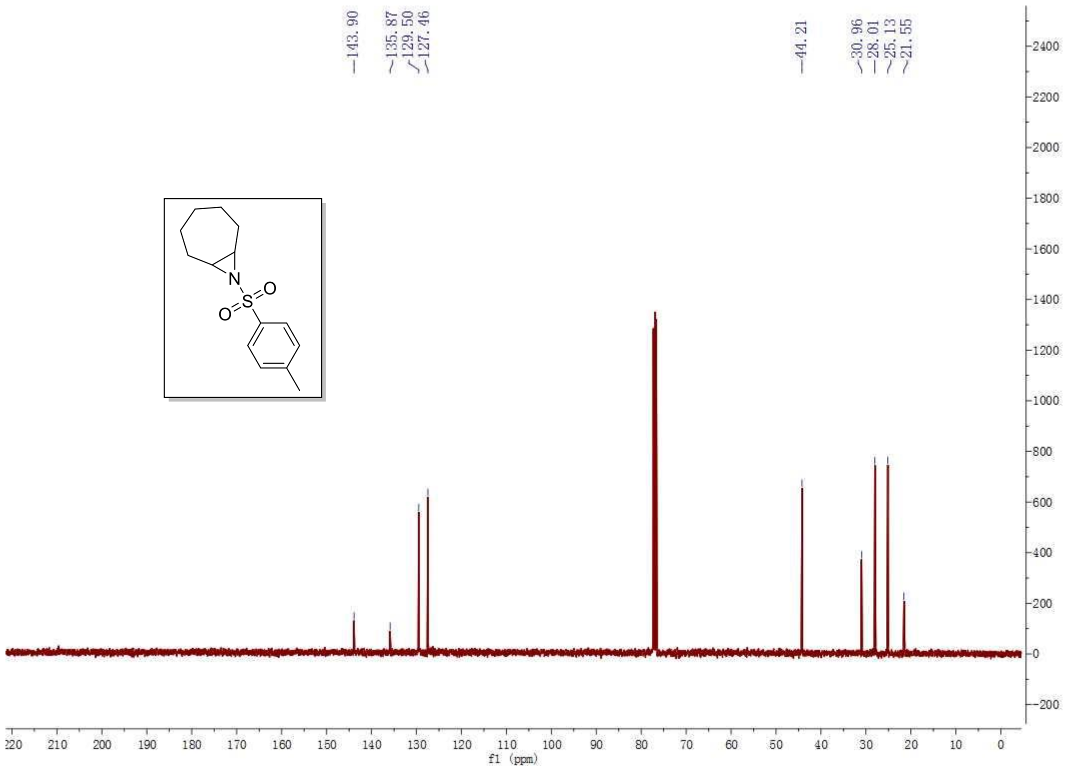

Supplementary Figure 12. ^{13}C NMR spectra for compound 9

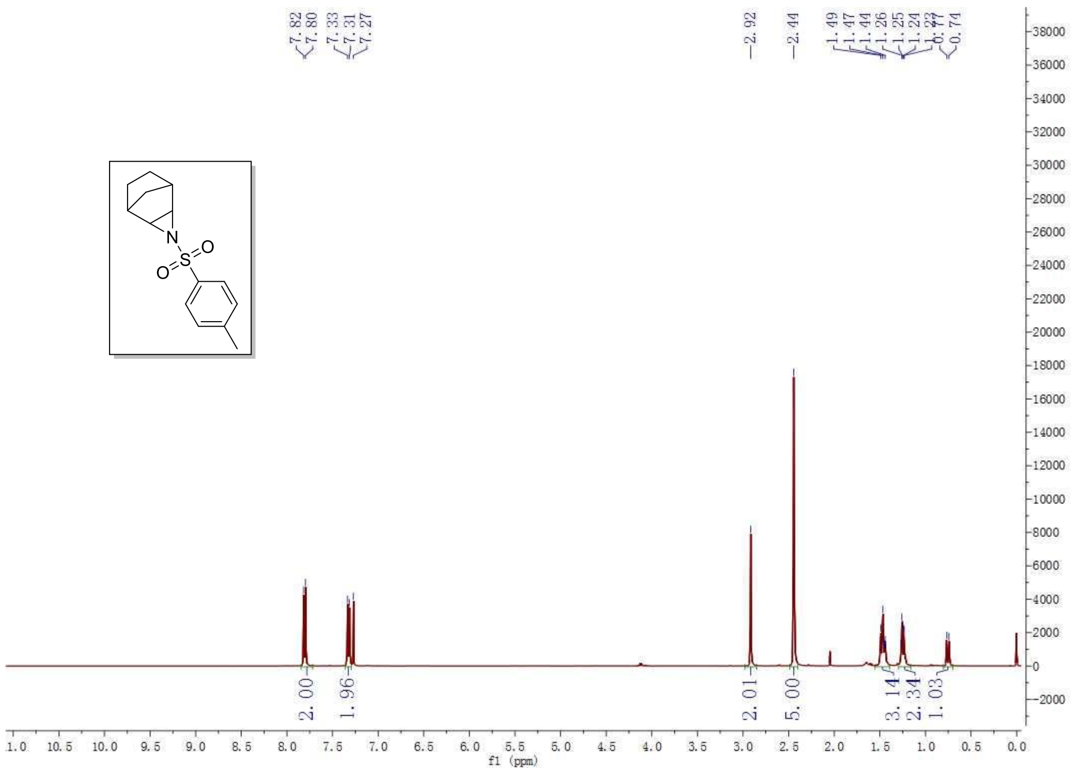

Supplementary Figure 13. ^{1}H NMR spectra for compound 10

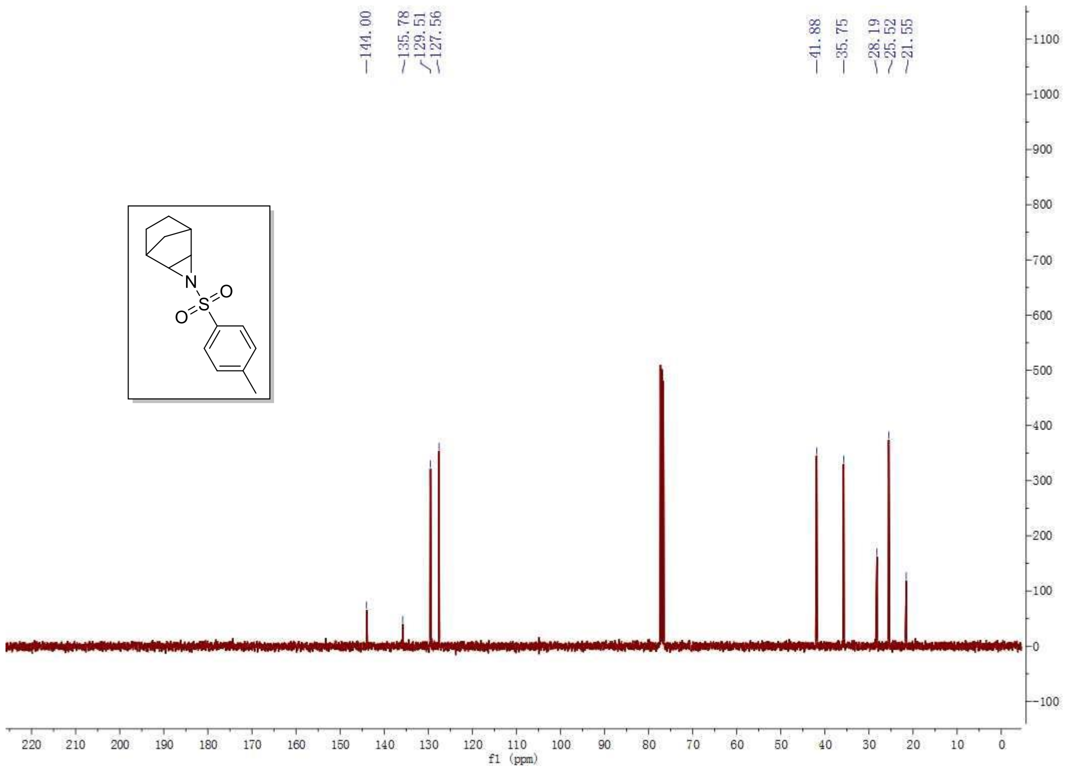

Supplementary Figure 14. ^{13}C NMR spectra for compound 10

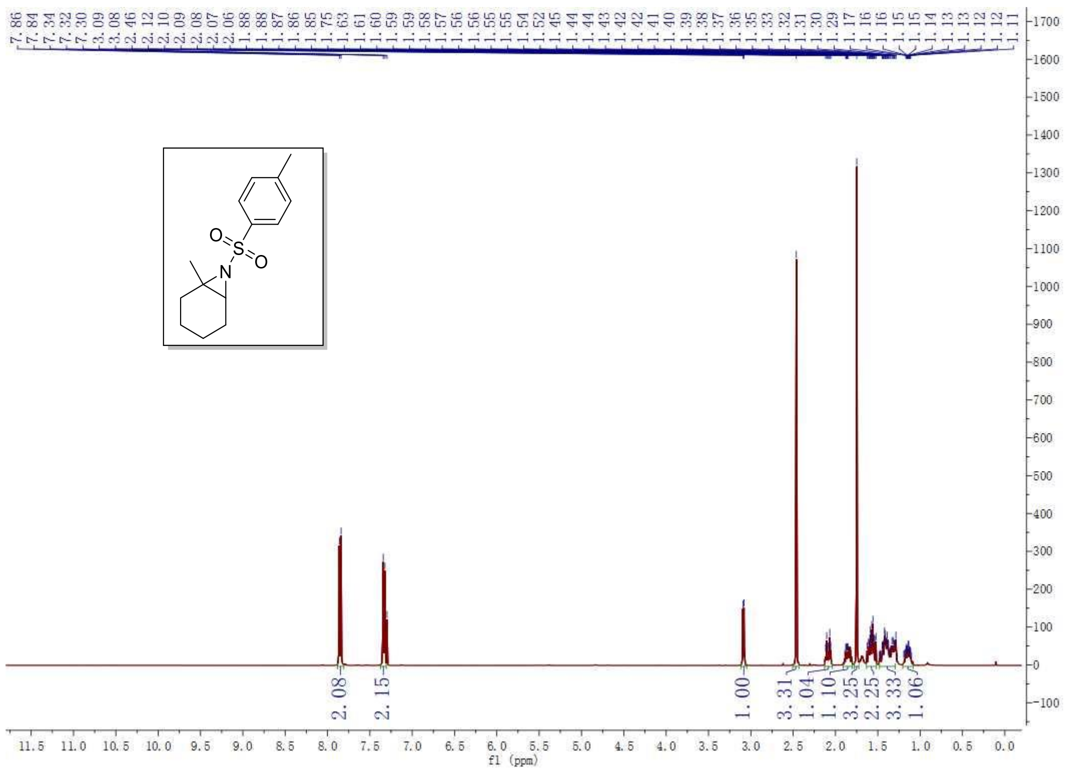

Supplementary Figure 15. ^{1}H NMR spectra for compound 11

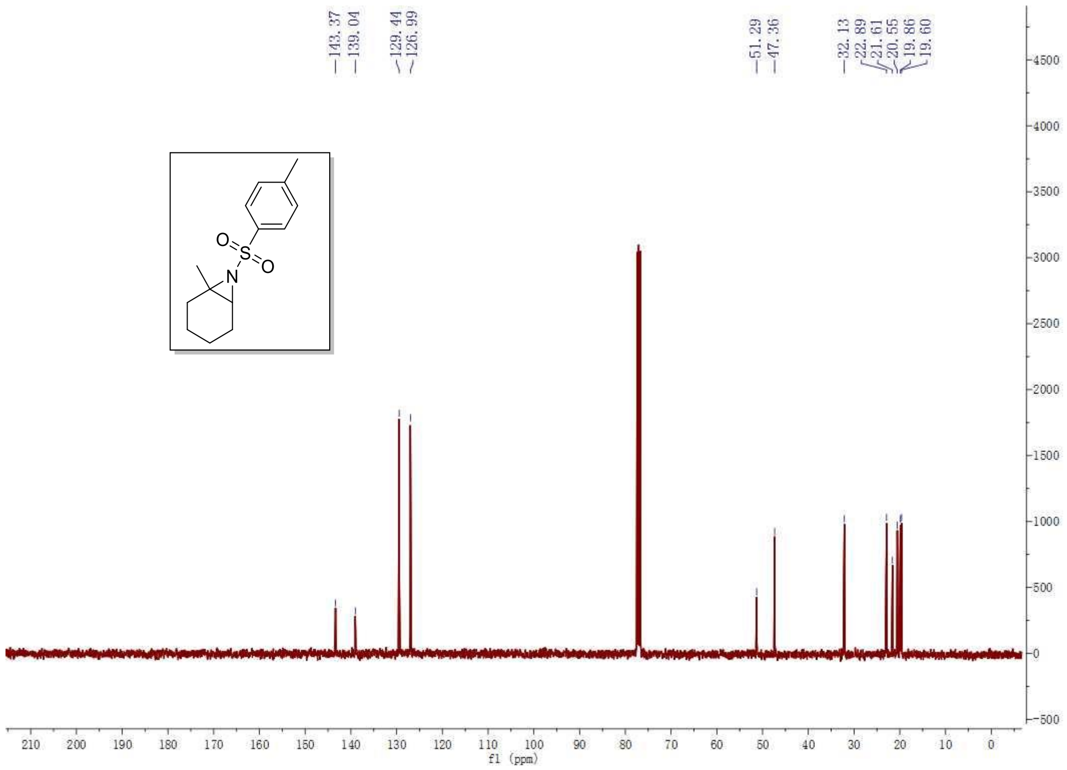

Supplementary Figure 16. ^{13}C NMR spectra for compound 11

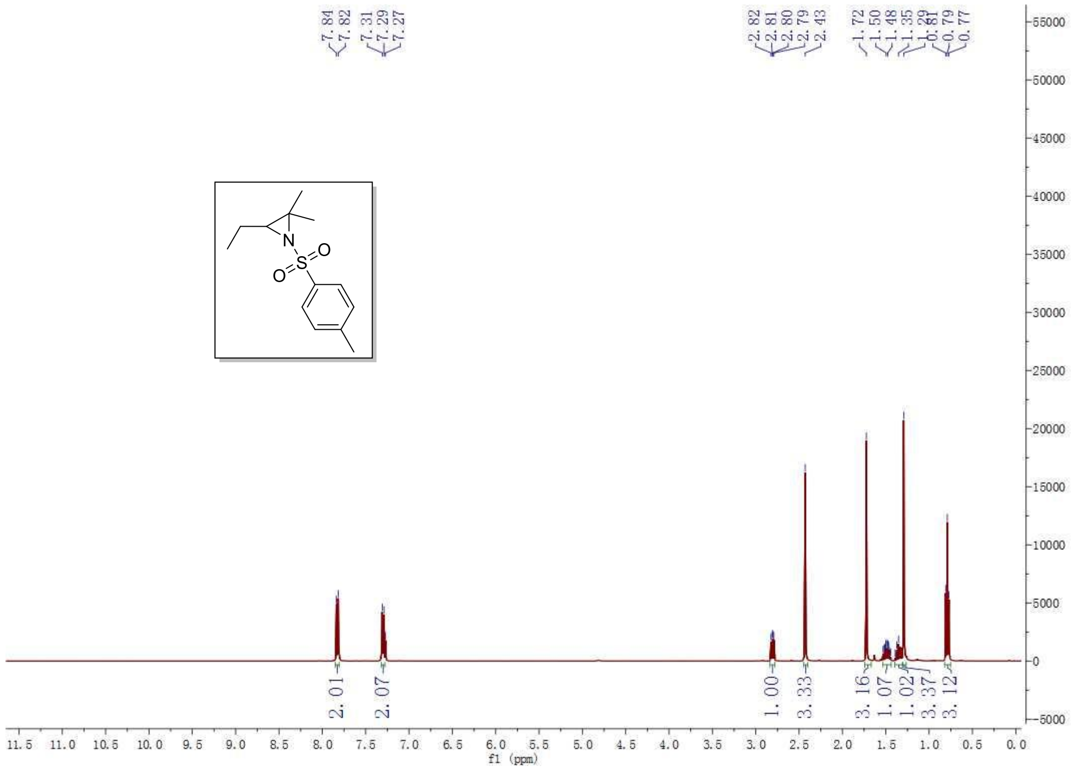

Supplementary Figure 17. ^{1}H NMR spectra for compound 12

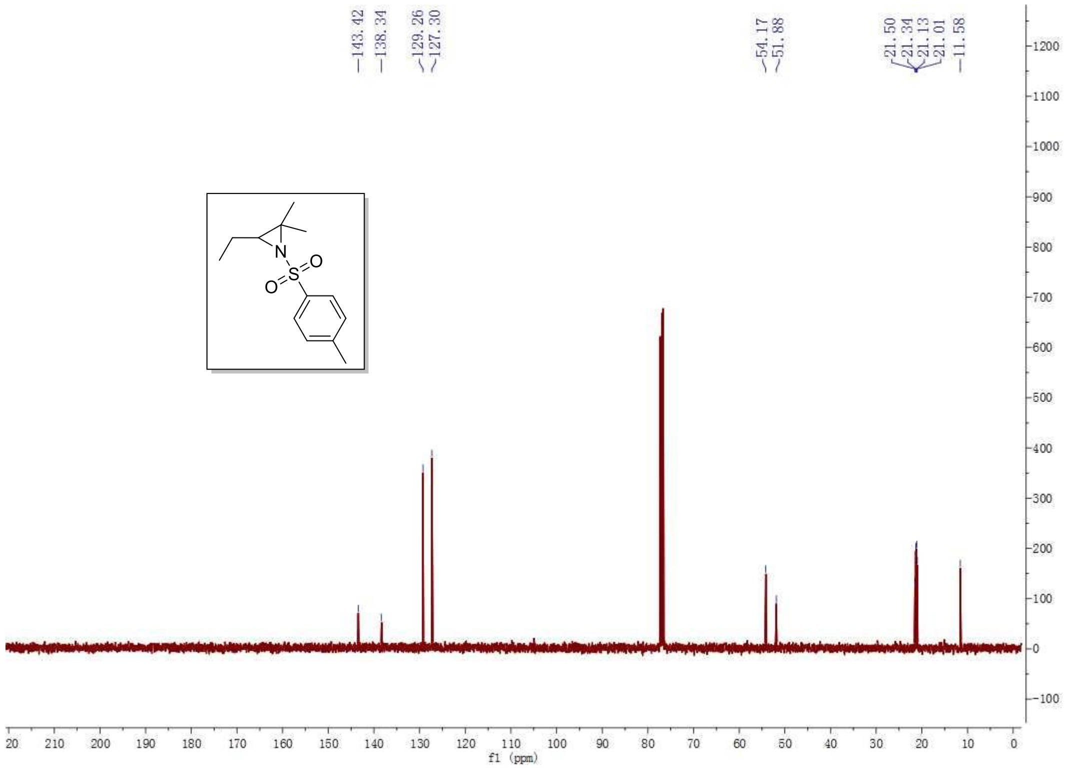

Supplementary Figure 18. ^{13}C NMR spectra for compound 12

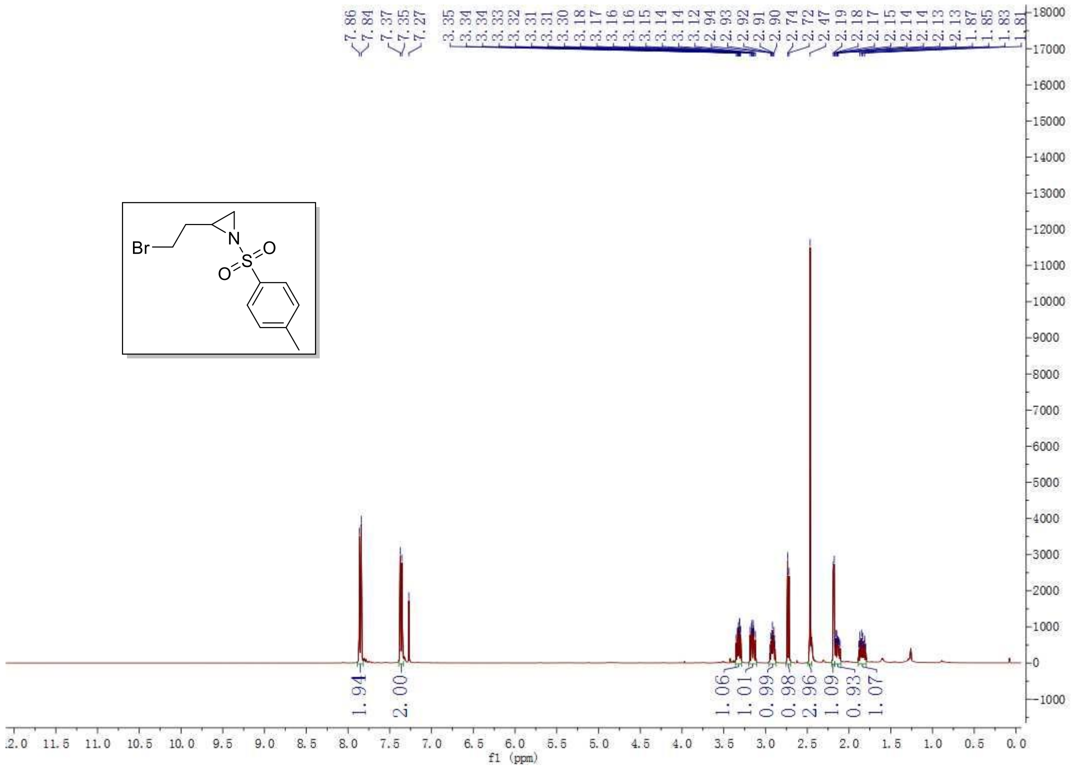

Supplementary Figure 19. ^{1}H NMR spectra for compound 13

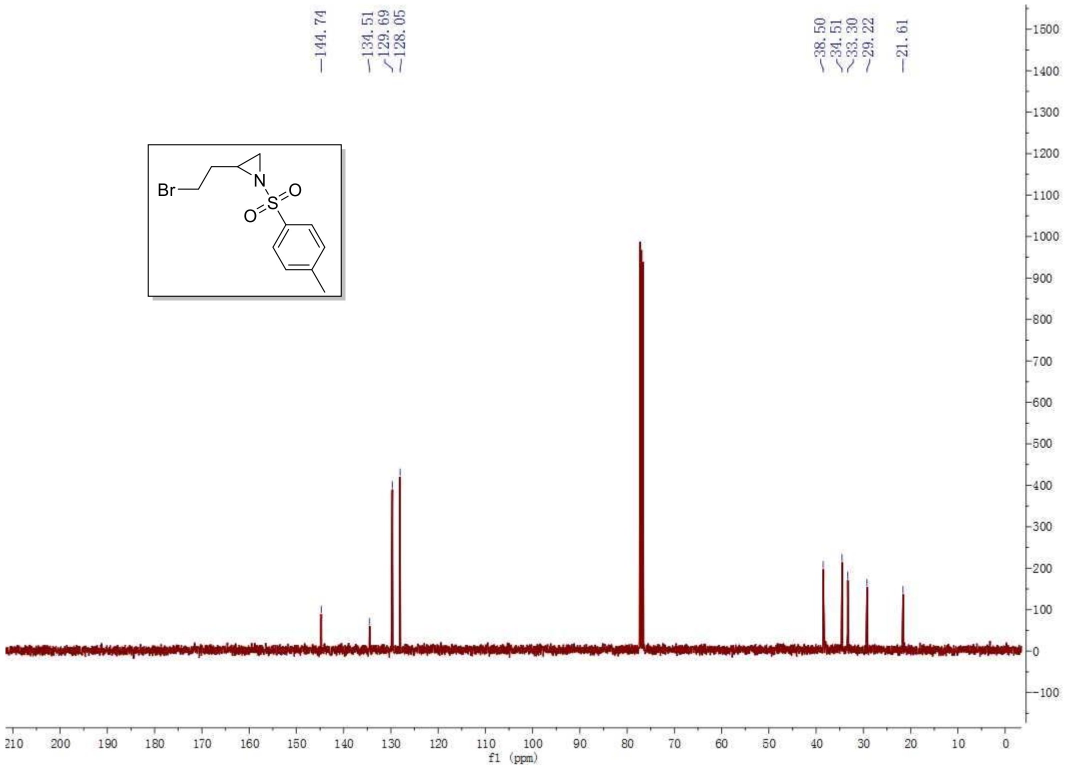

Supplementary Figure 20. ^{13}C NMR spectra for compound 13

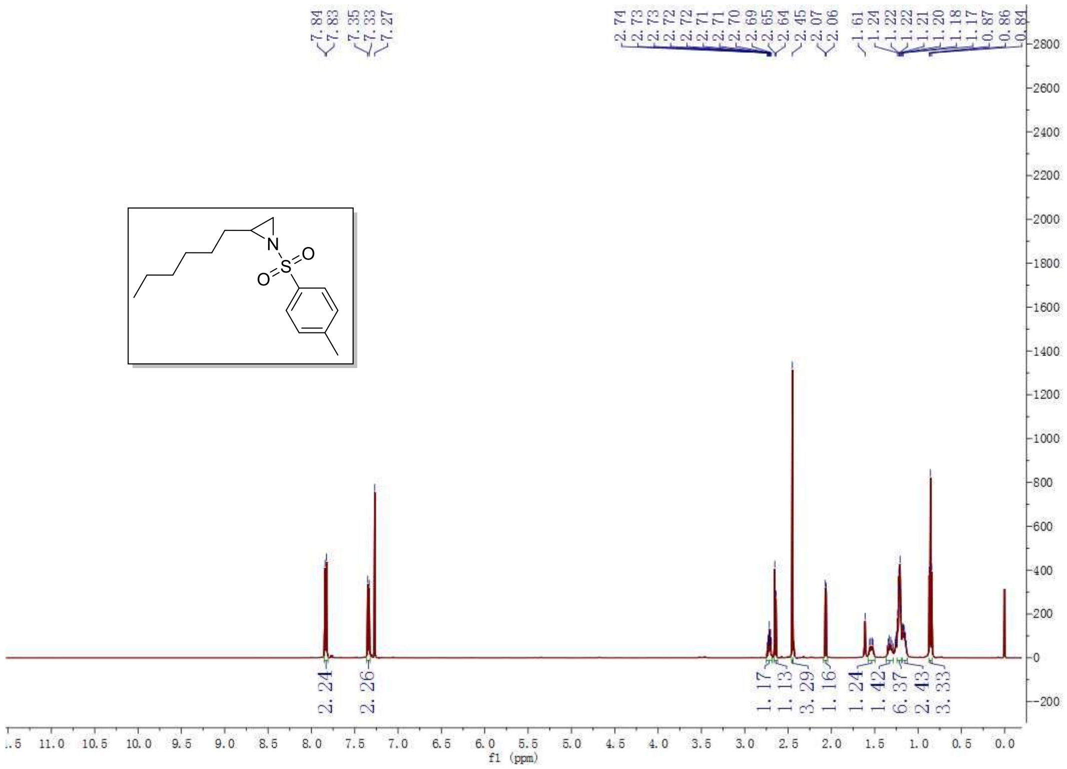

Supplementary Figure 21. ^{1}H NMR spectra for compound 14

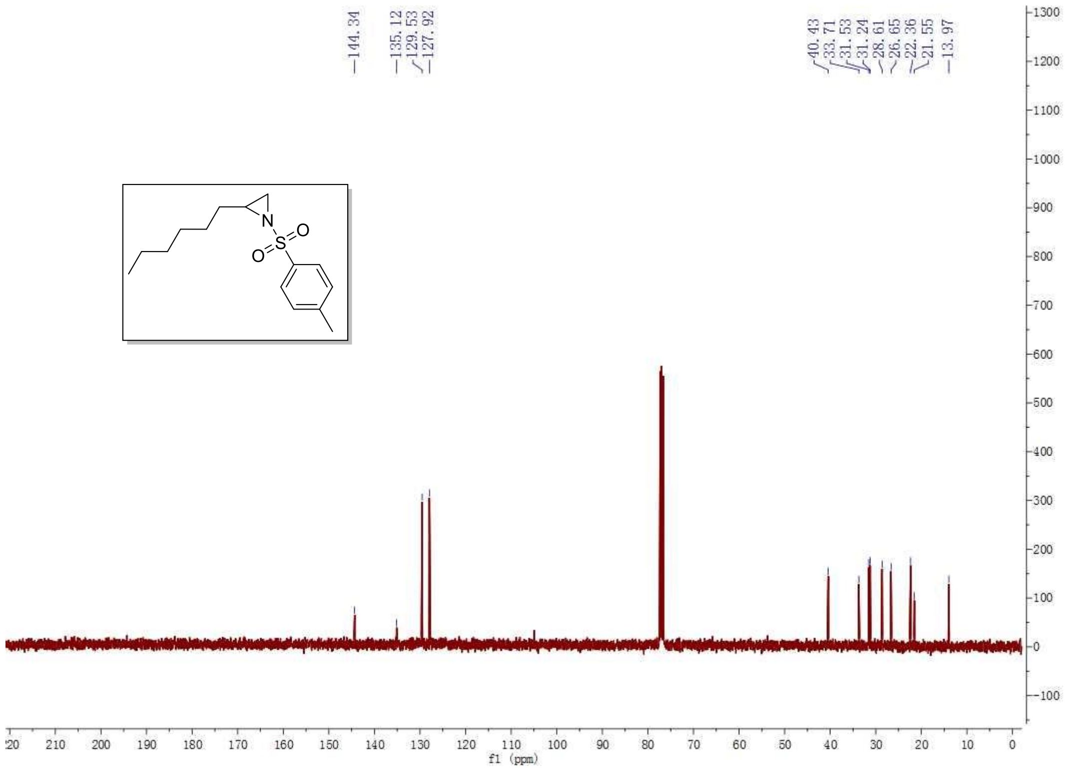

Supplementary Figure 22. ^{13}C NMR spectra for compound 14

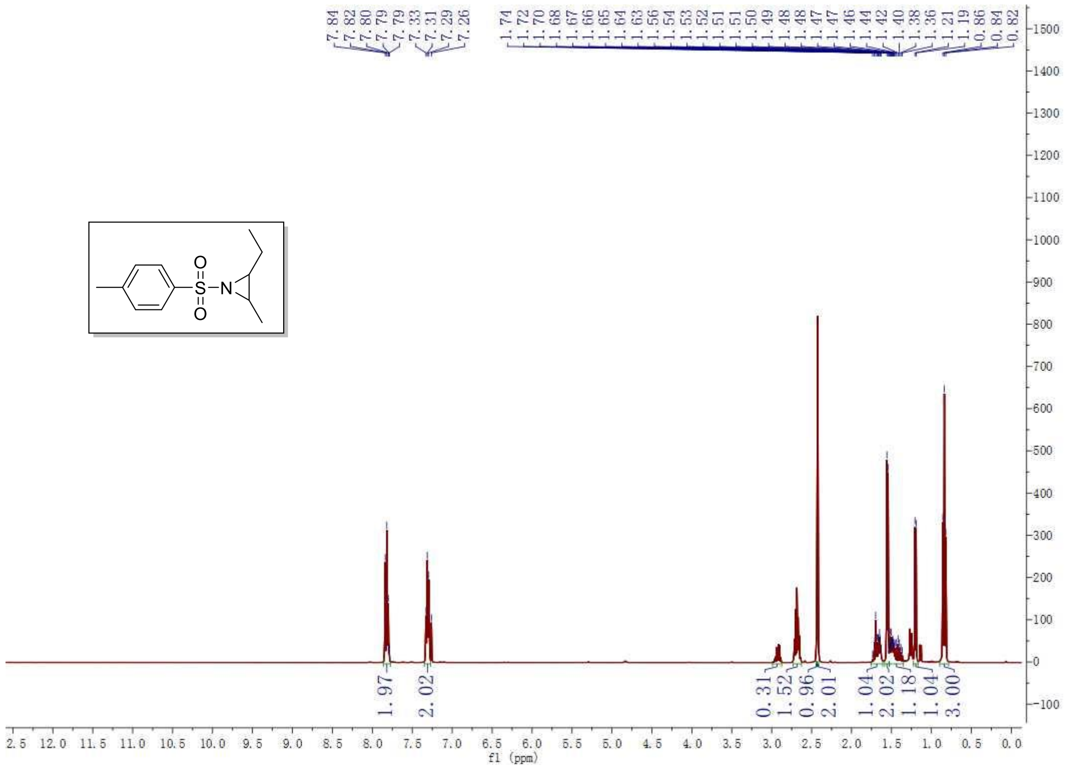

Supplementary Figure 23. ^{1}H NMR spectra for compound 15

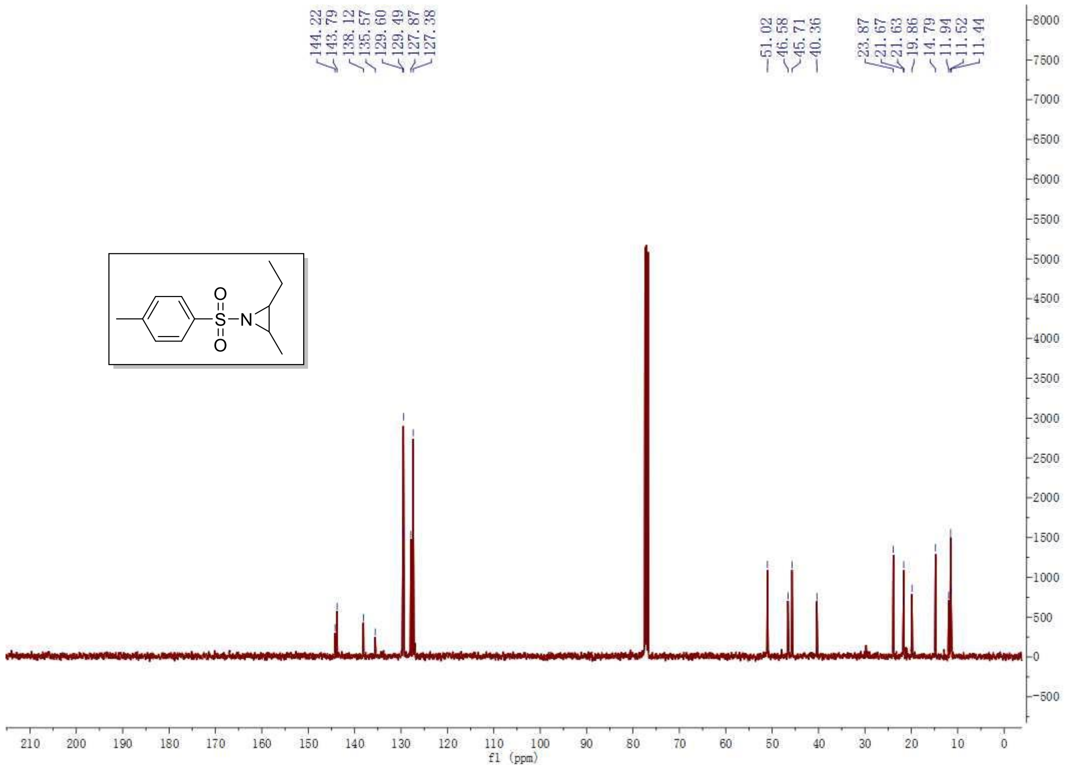

Supplementary Figure 24. ^{13}C NMR spectra for compound 15

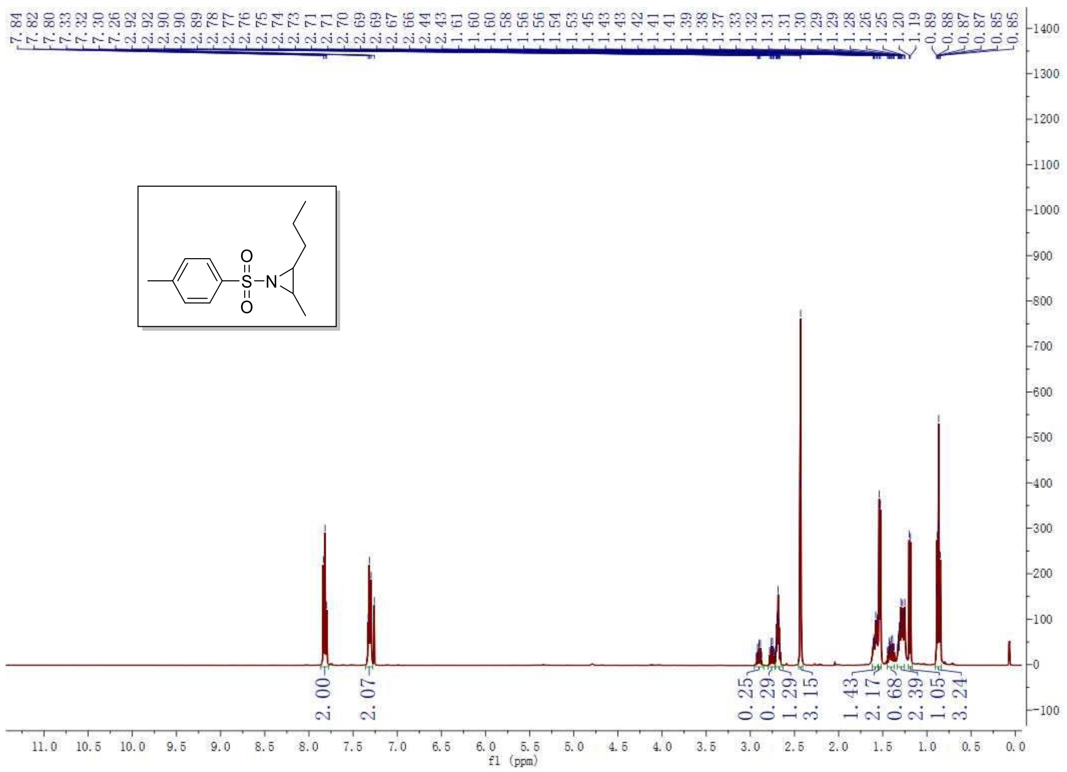

Supplementary Figure 25. ^{1}H NMR spectra for compound 16

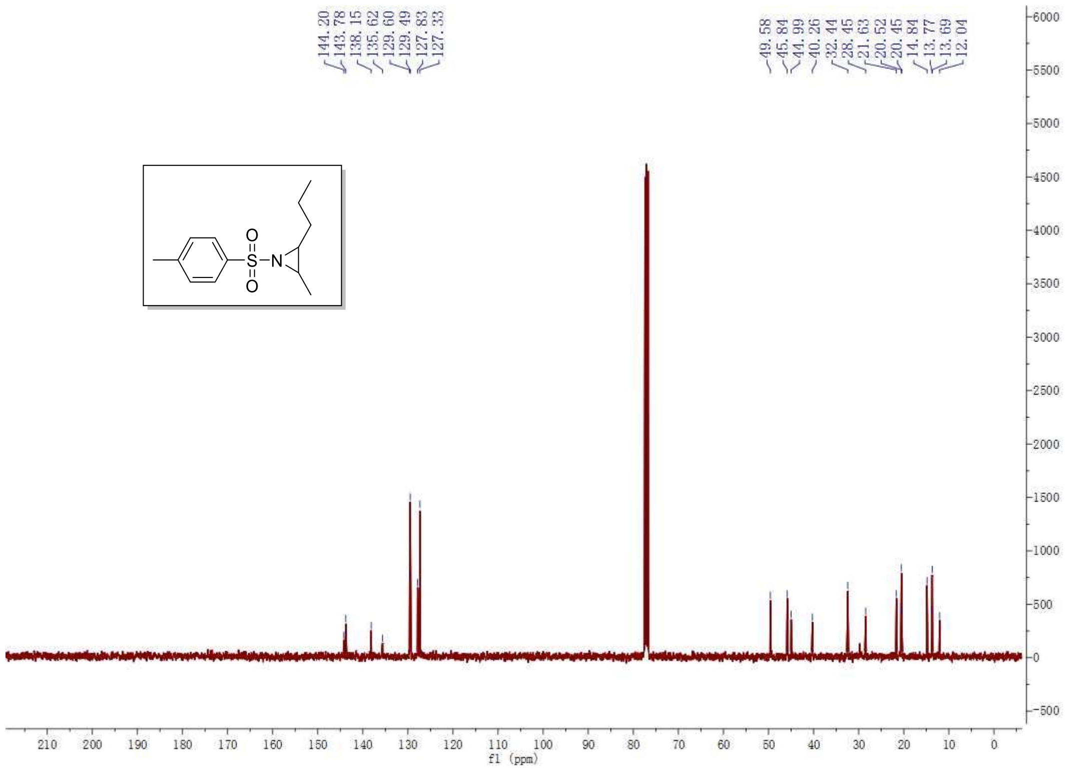

Supplementary Figure 26. ^{13}C NMR spectra for compound 16

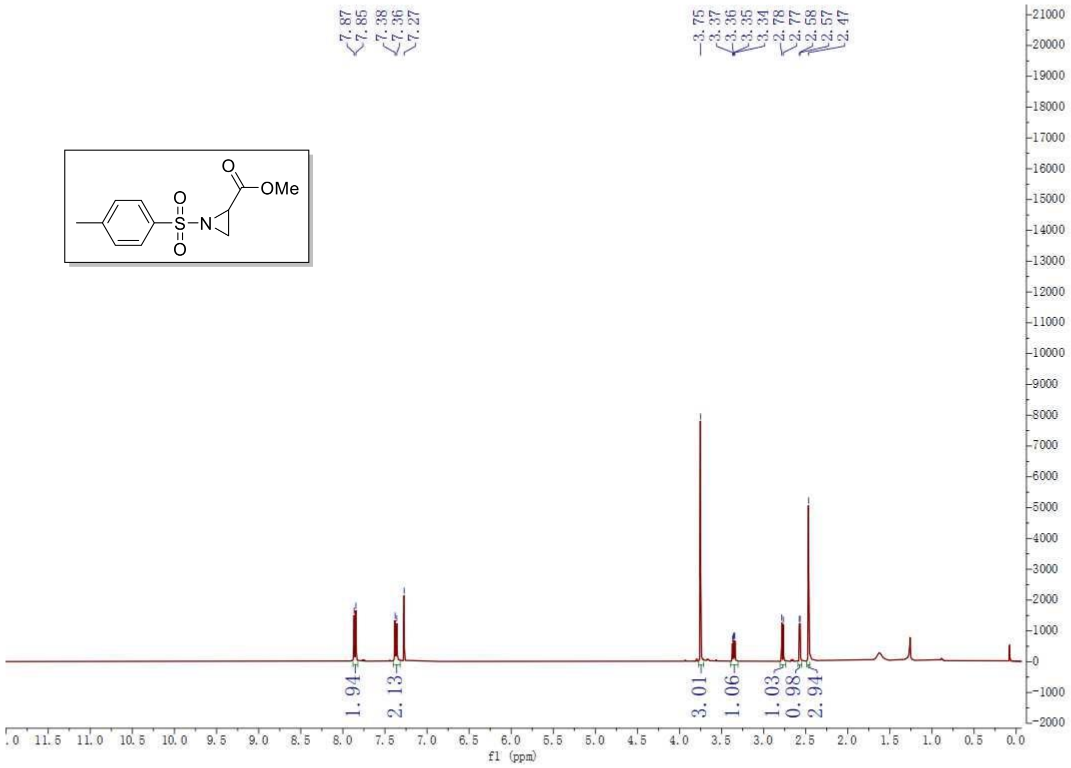

Supplementary Figure 27. ^{1}H NMR spectra for compound 17

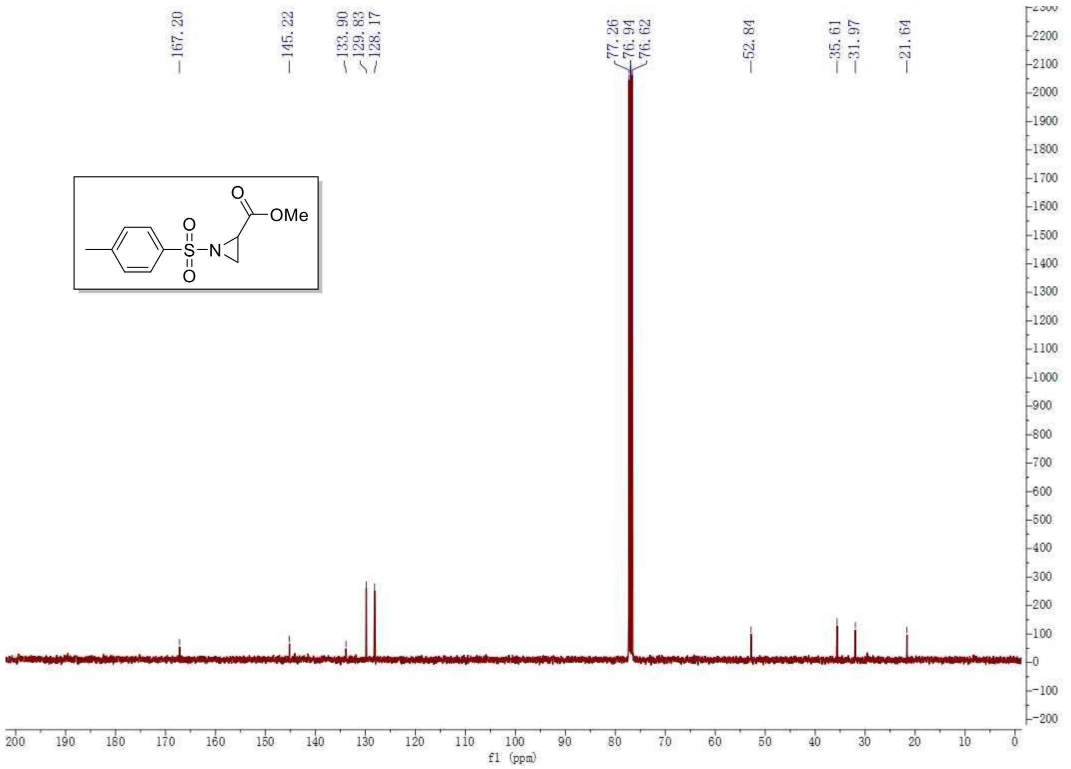

Supplementary Figure 28. ^{13}C NMR spectra for compound 17

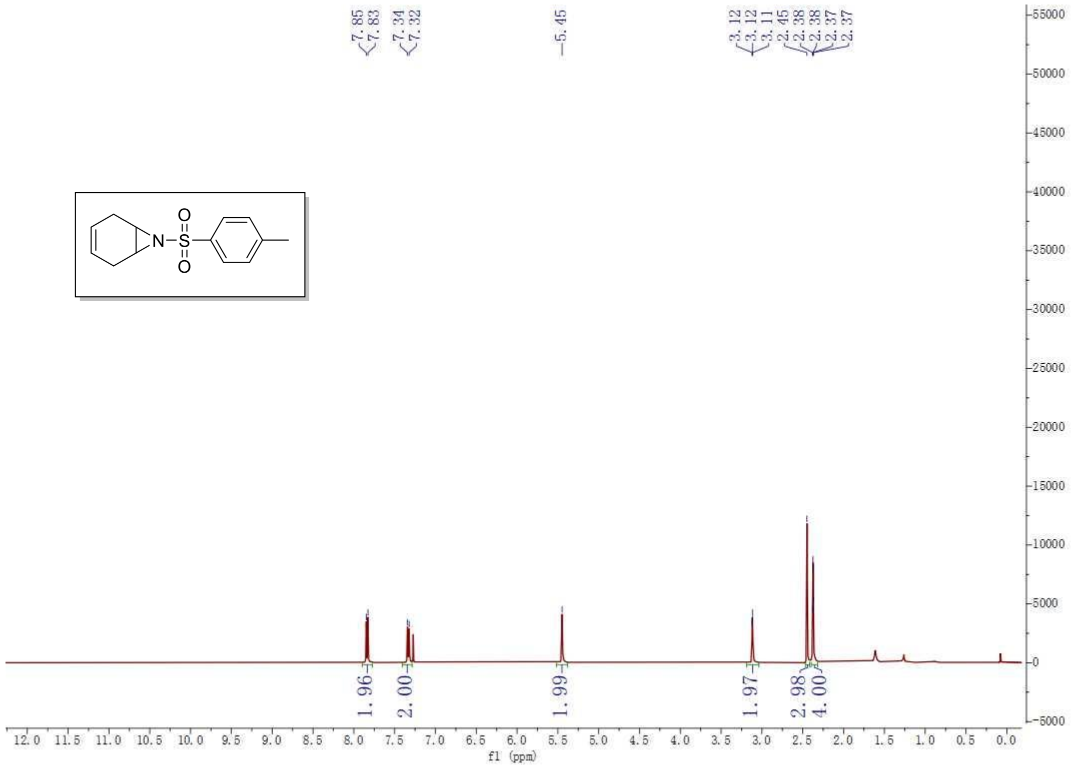

Supplementary Figure 29. ^{1}H NMR spectra for compound 18

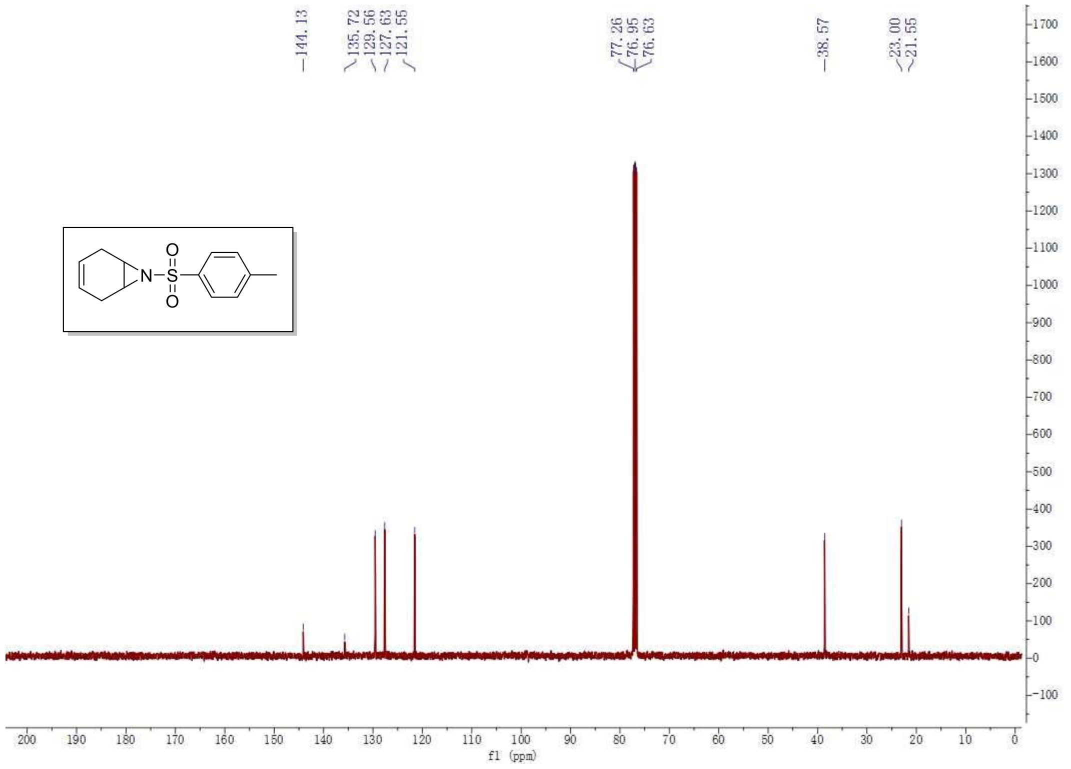

Supplementary Figure 30. ^{13}C NMR spectra for compound 18

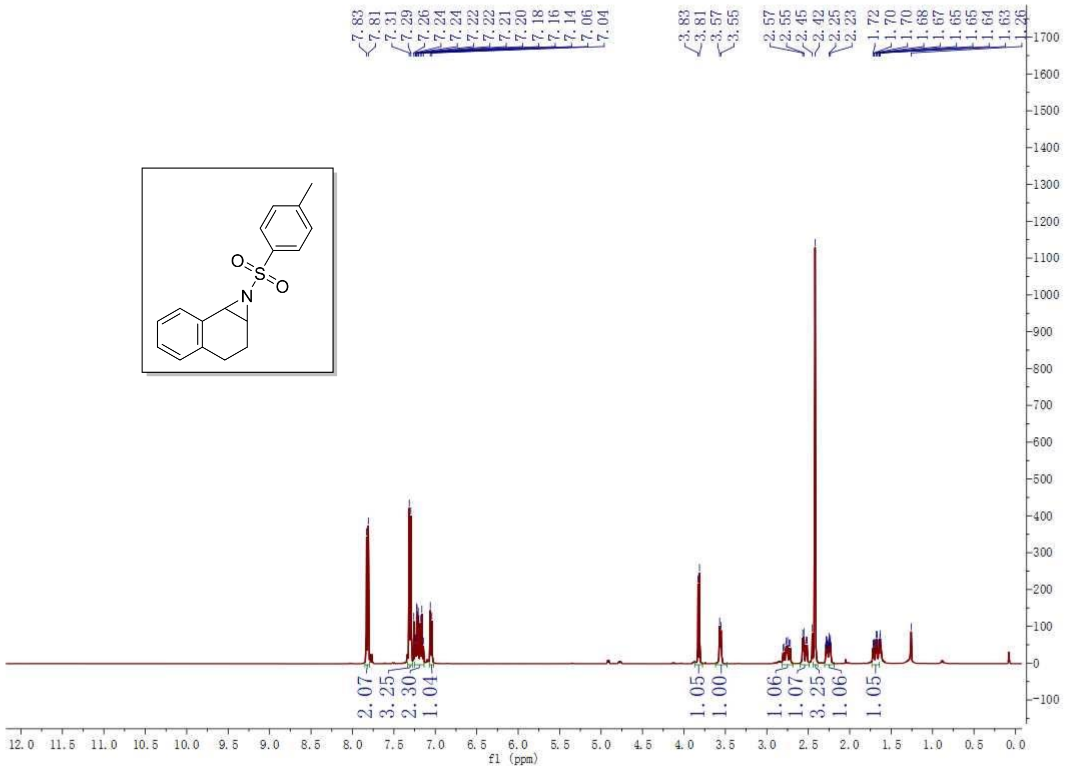

Supplementary Figure 31. ^{1}H NMR spectra for compound 19

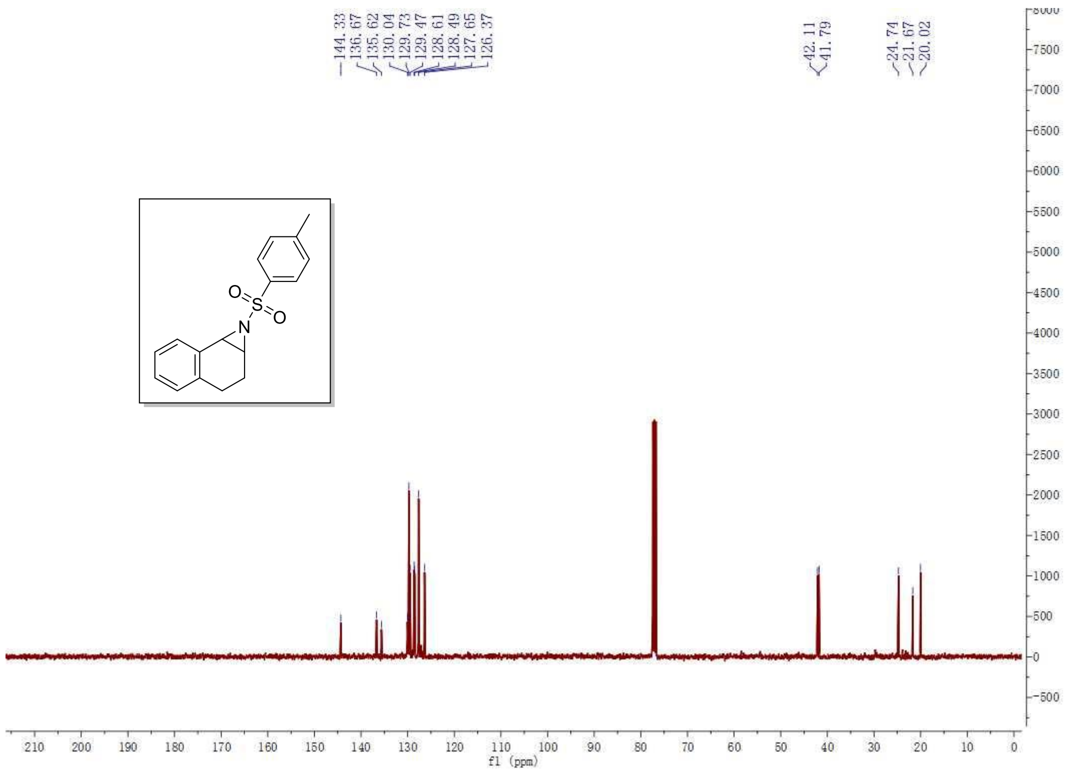

Supplementary Figure 32. ^{13}C NMR spectra for compound 19

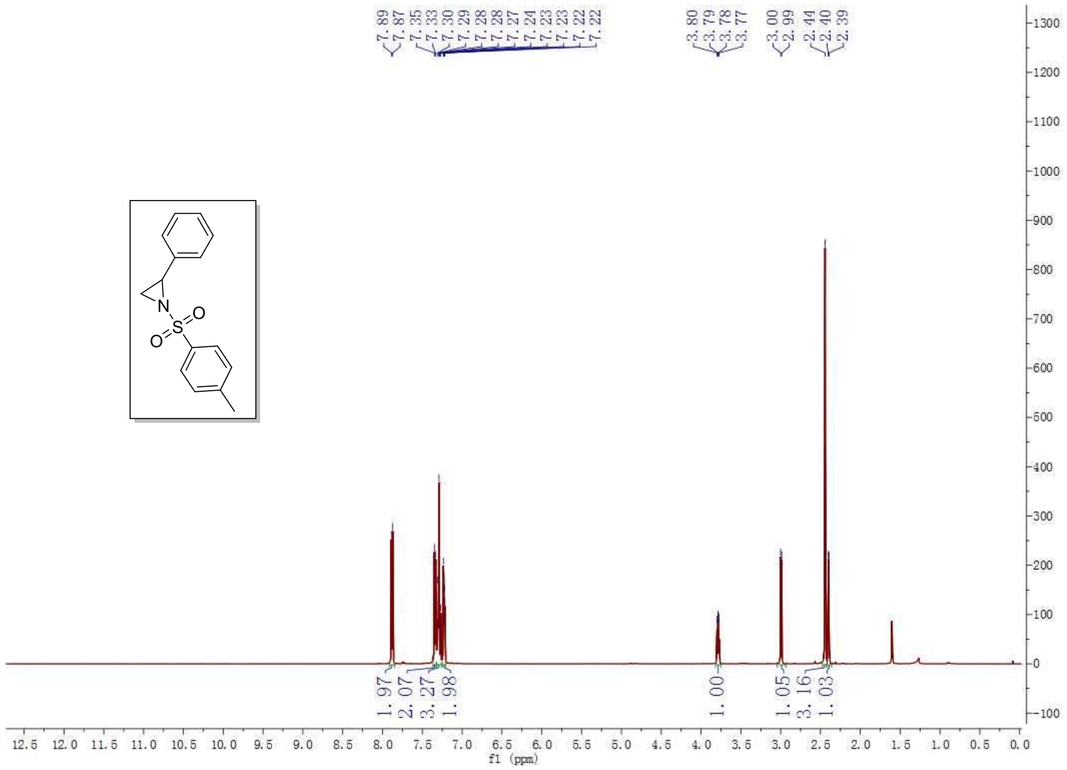

Supplementary Figure 33. ¹H NMR spectra for compound 20

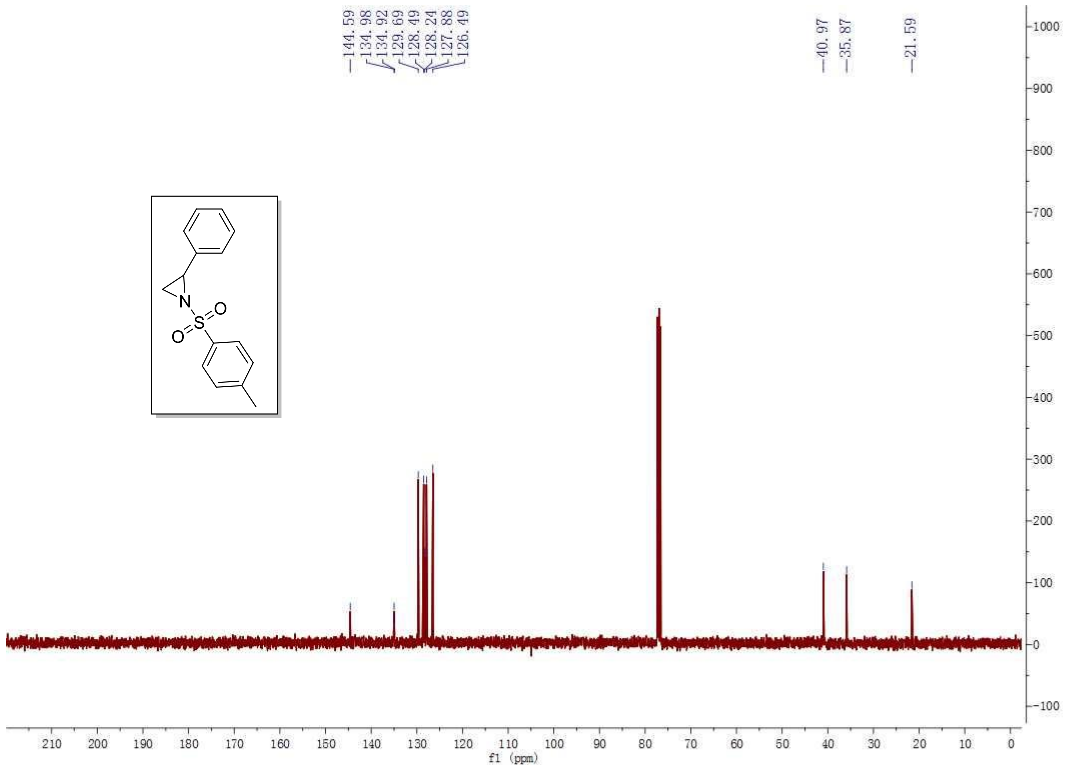

Supplementary Figure 34. ¹³C NMR spectra for compound 20

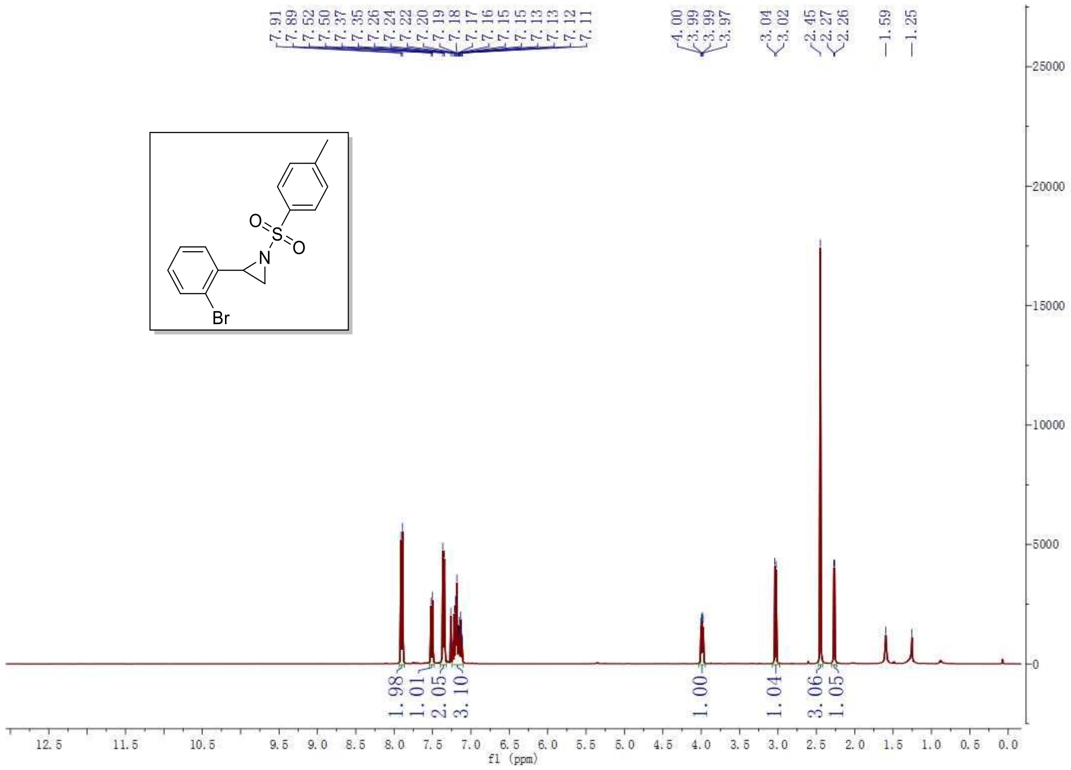

Supplementary Figure 35. ^{1}H NMR spectra for compound 21

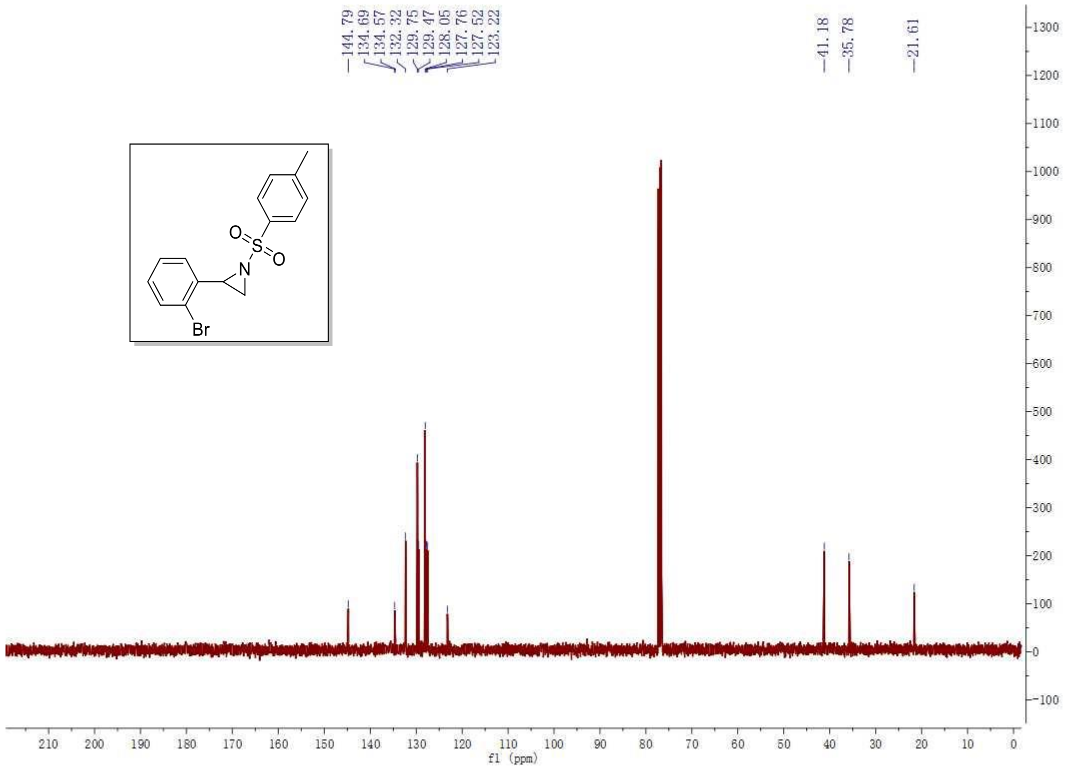

Supplementary Figure 36. ^{13}C NMR spectra for compound 21

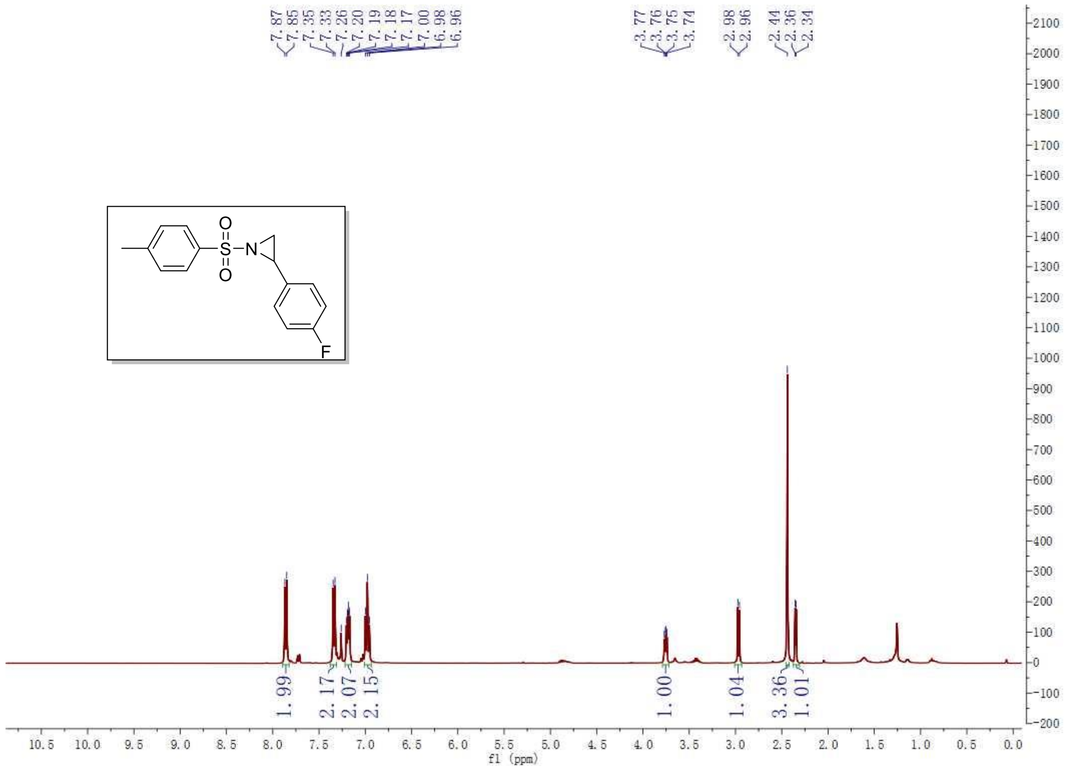

Supplementary Figure 37. ^{1}H NMR spectra for compound 22

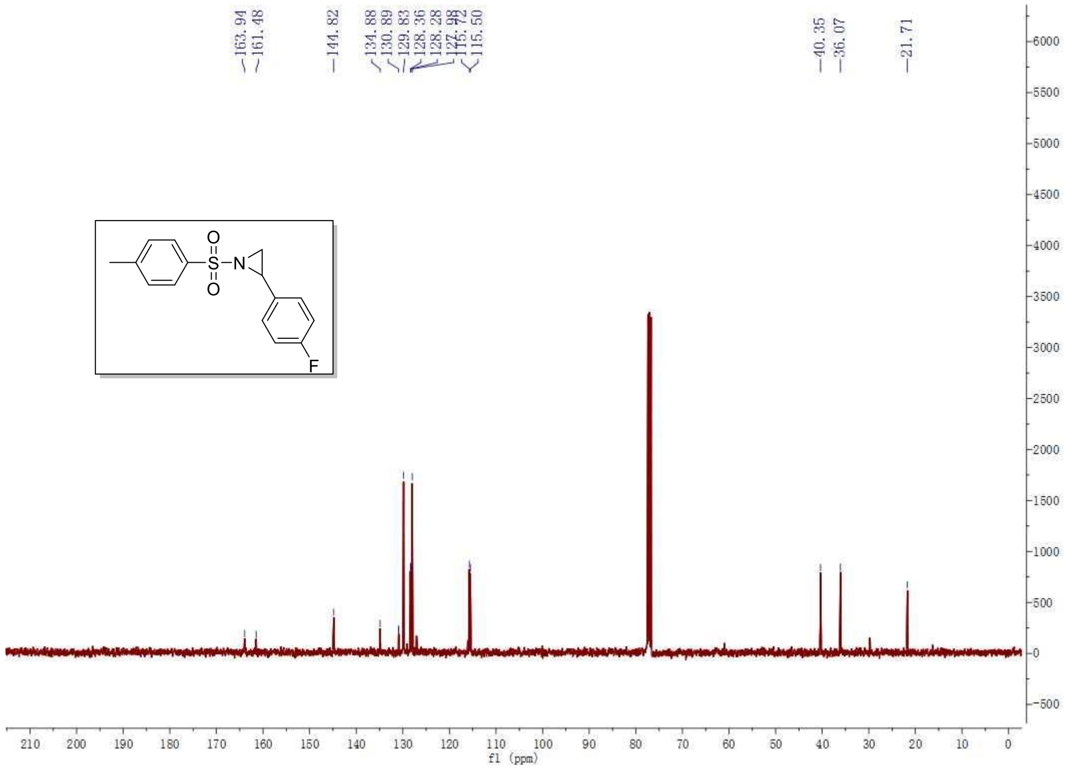

Supplementary Figure 38. ^{13}C NMR spectra for compound 22

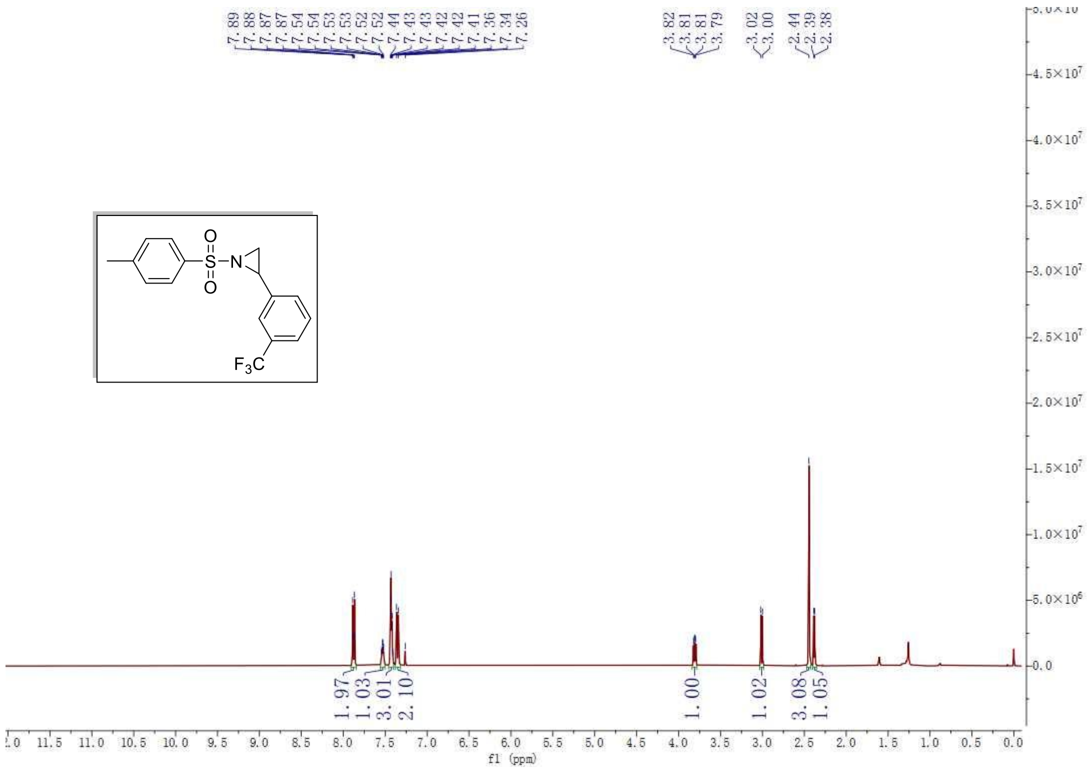

Supplementary Figure 39. ^{1}H NMR spectra for compound 23

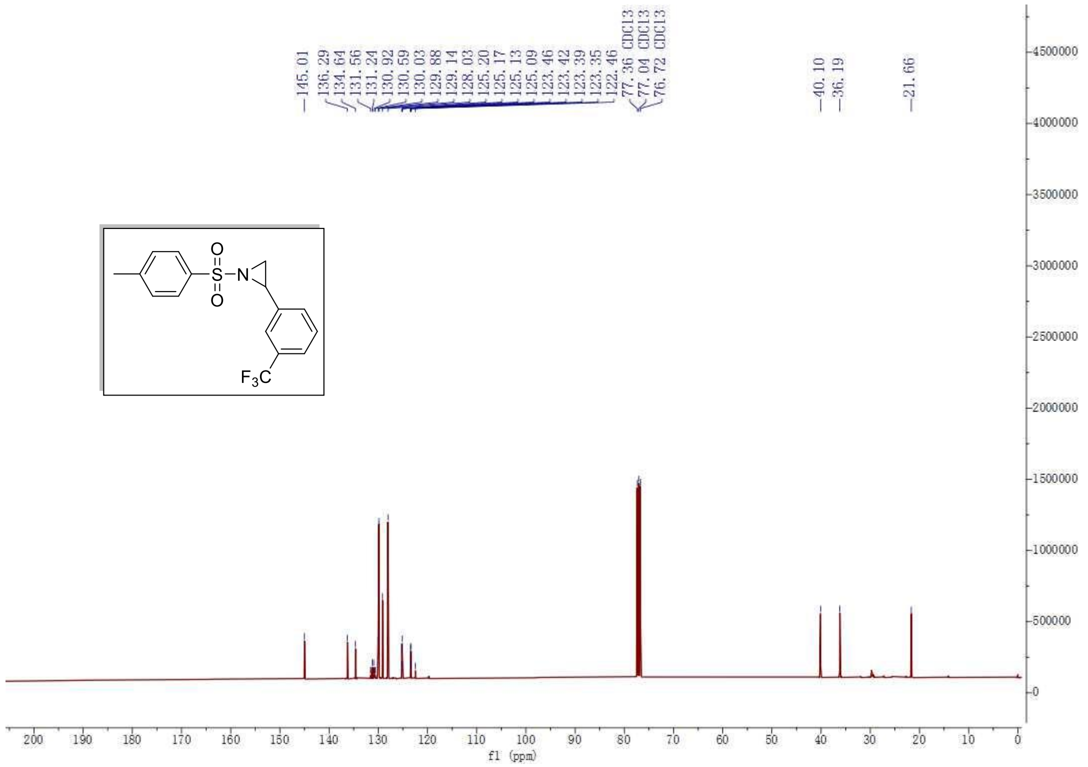

Supplementary Figure 40. ^{13}C NMR spectra for compound 23

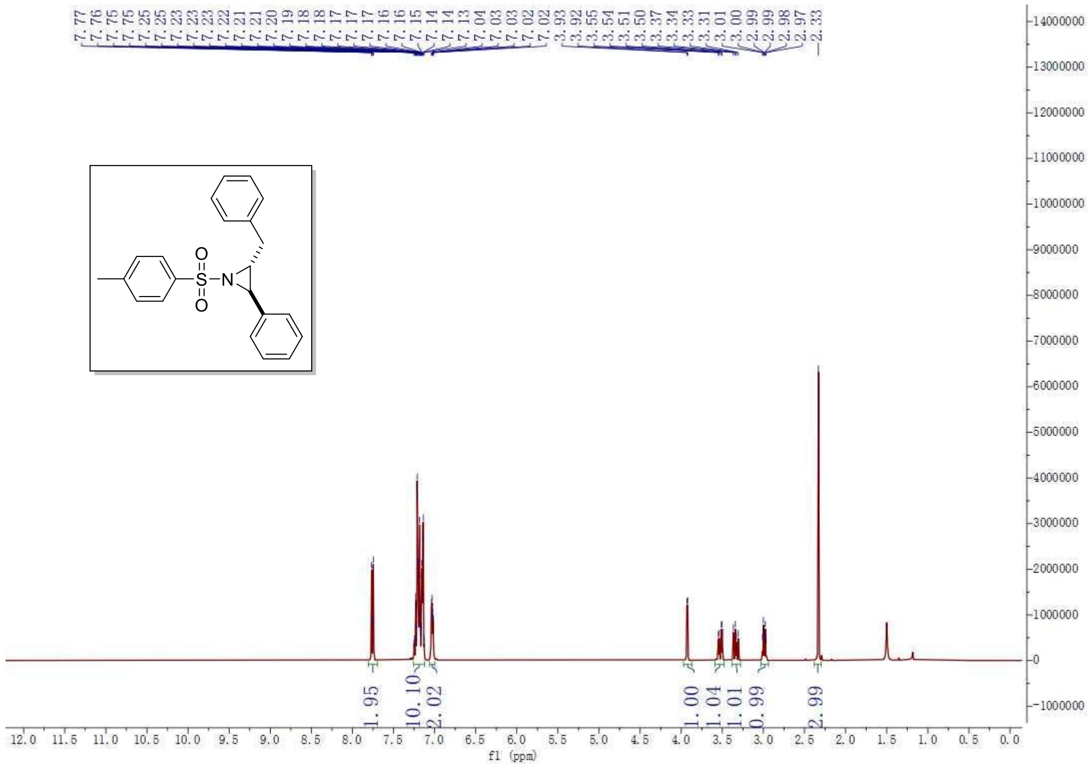

Supplementary Figure 41. ^{1}H NMR spectra for compound 24

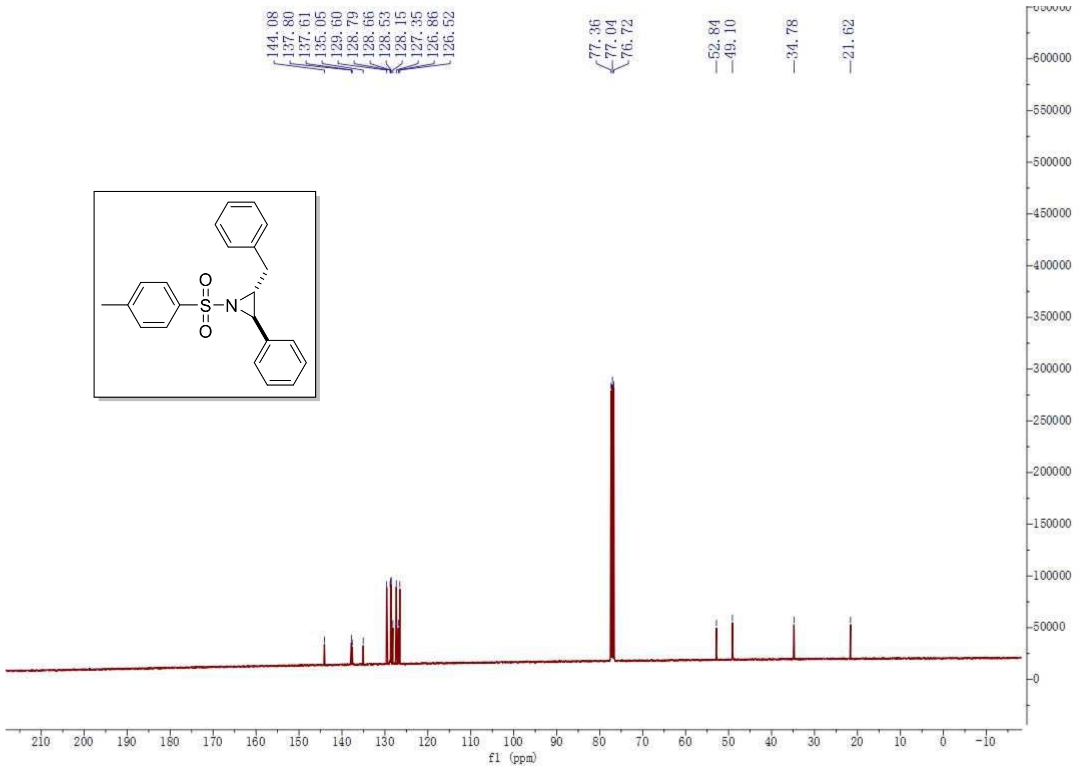

Supplementary Figure 42. ^{13}C NMR spectra for compound 24

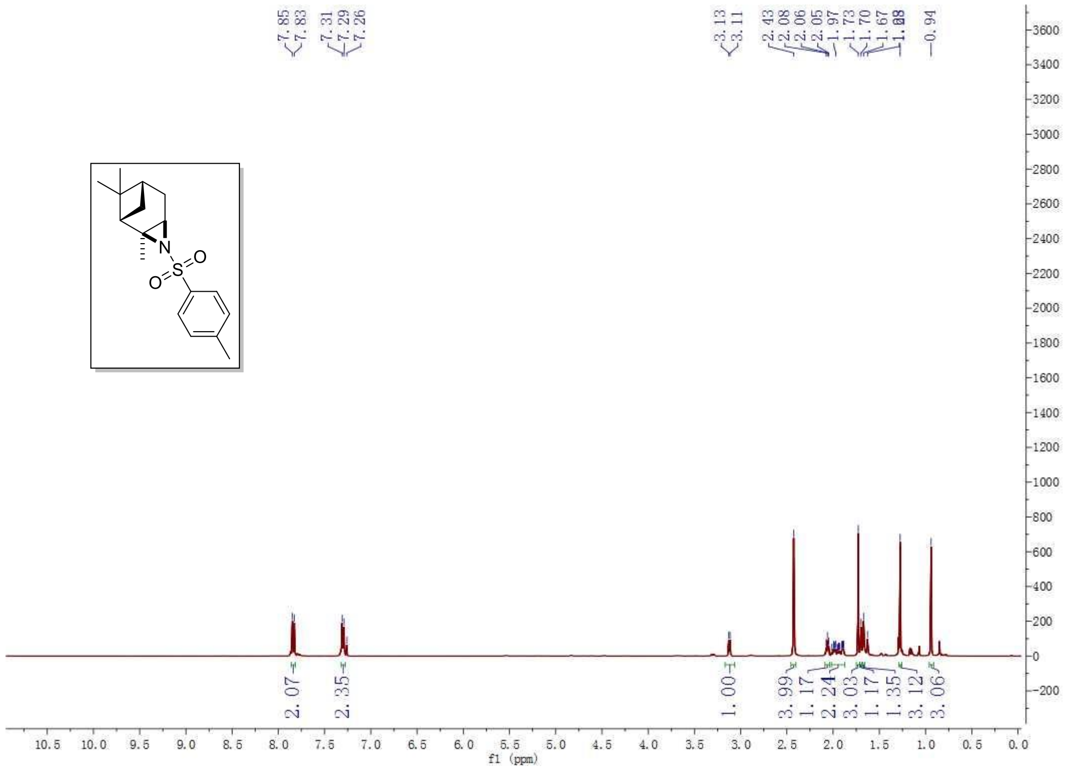

Supplementary Figure 43. ^{1}H NMR spectra for compound 25a

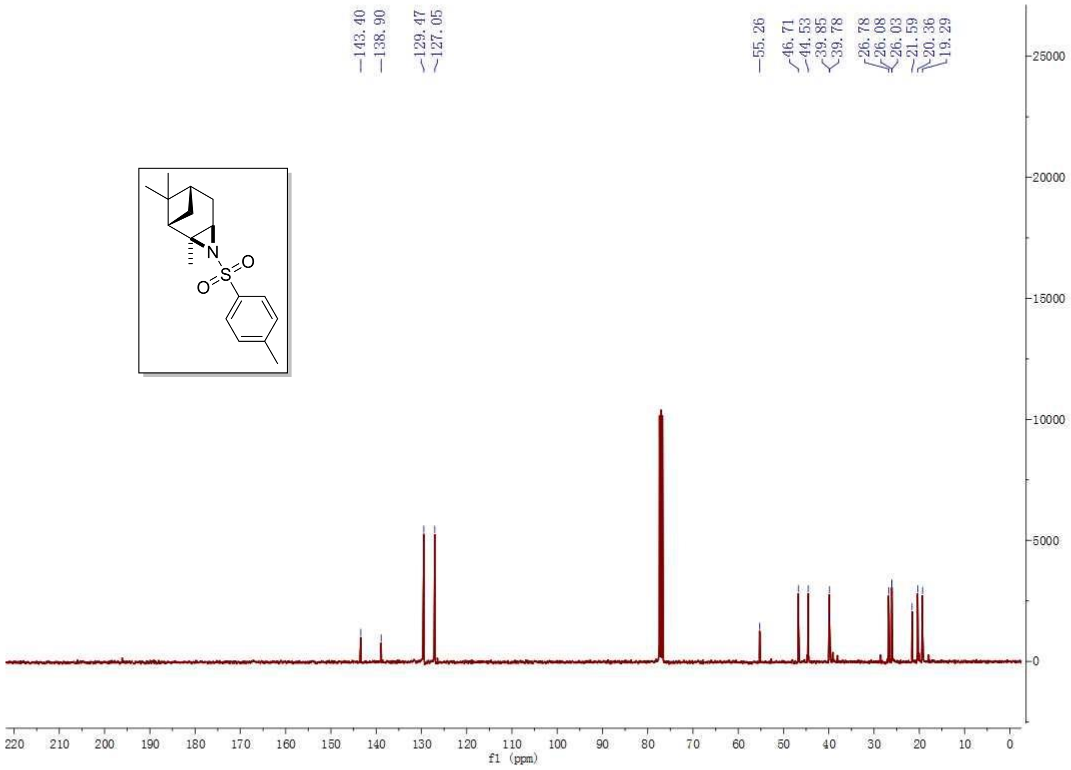

Supplementary Figure 44. ^{13}C NMR spectra for compound 25a

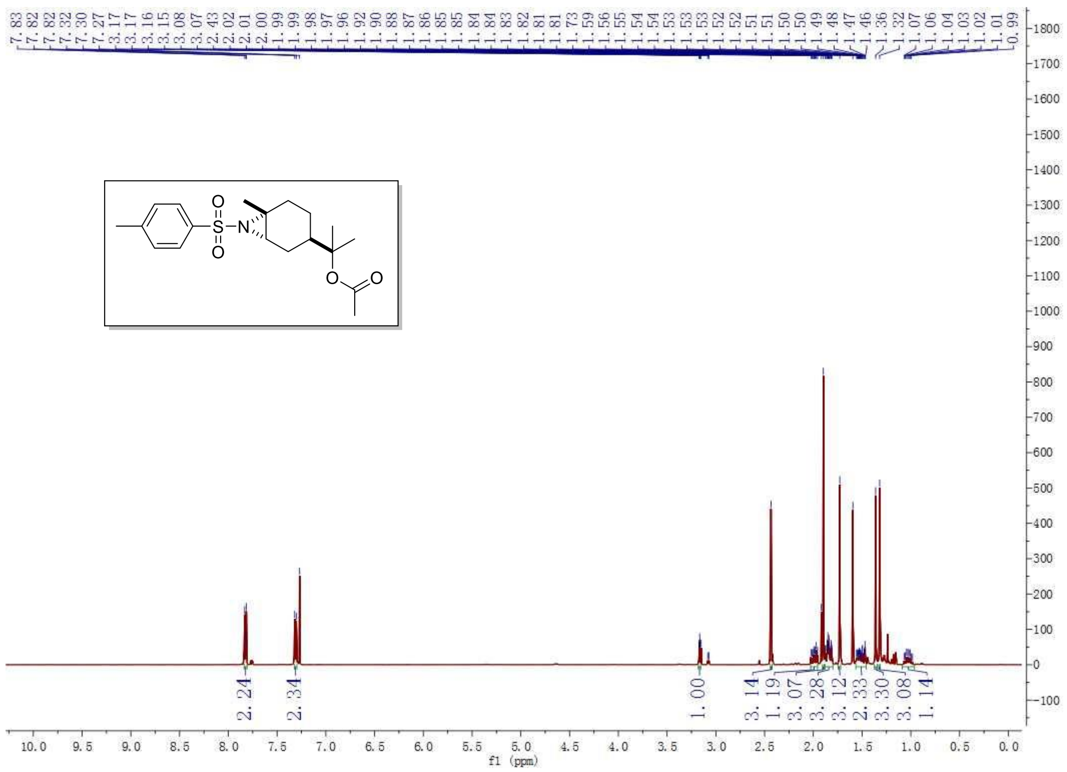

Supplementary Figure 45. ^{1}H NMR spectra for compound 26a

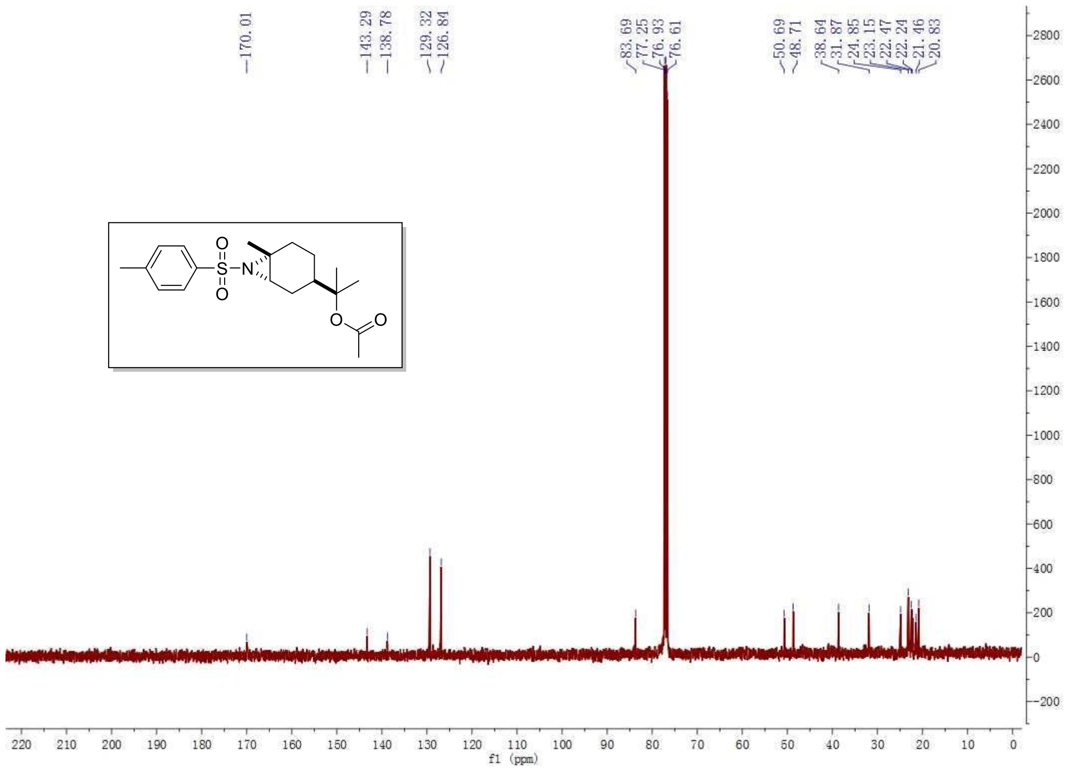

Supplementary Figure 46. ^{13}C NMR spectra for compound 26a

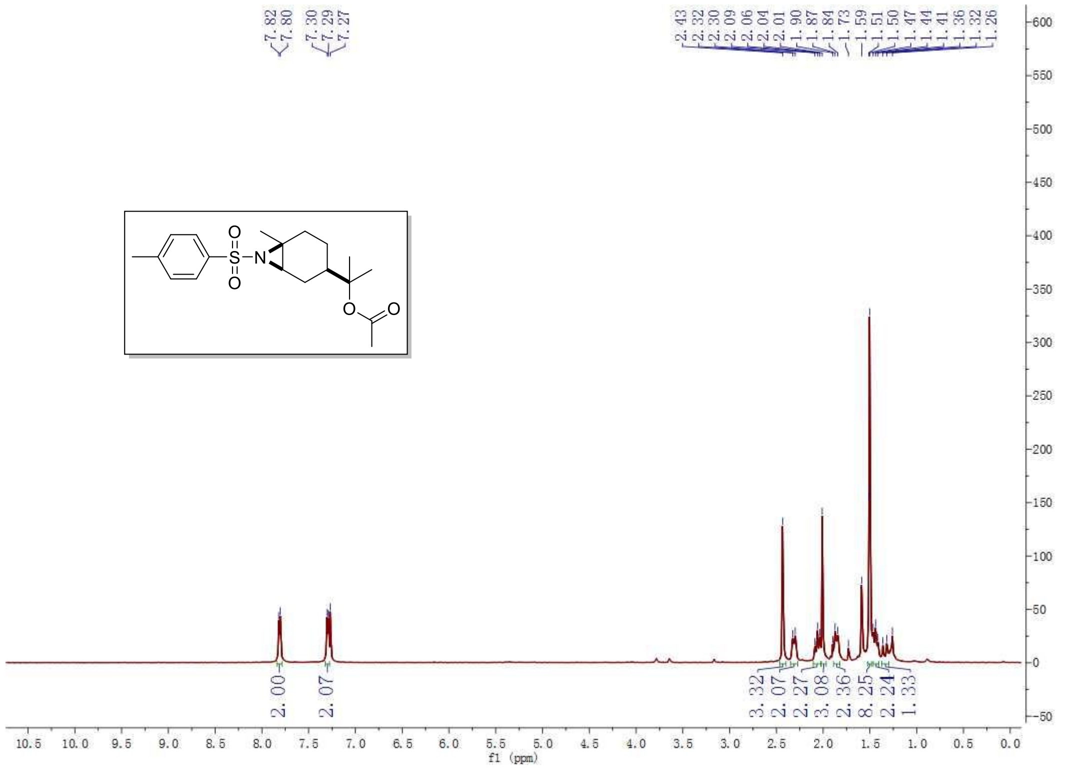

Supplementary Figure 47. ^{1}H NMR spectra for compound 26b

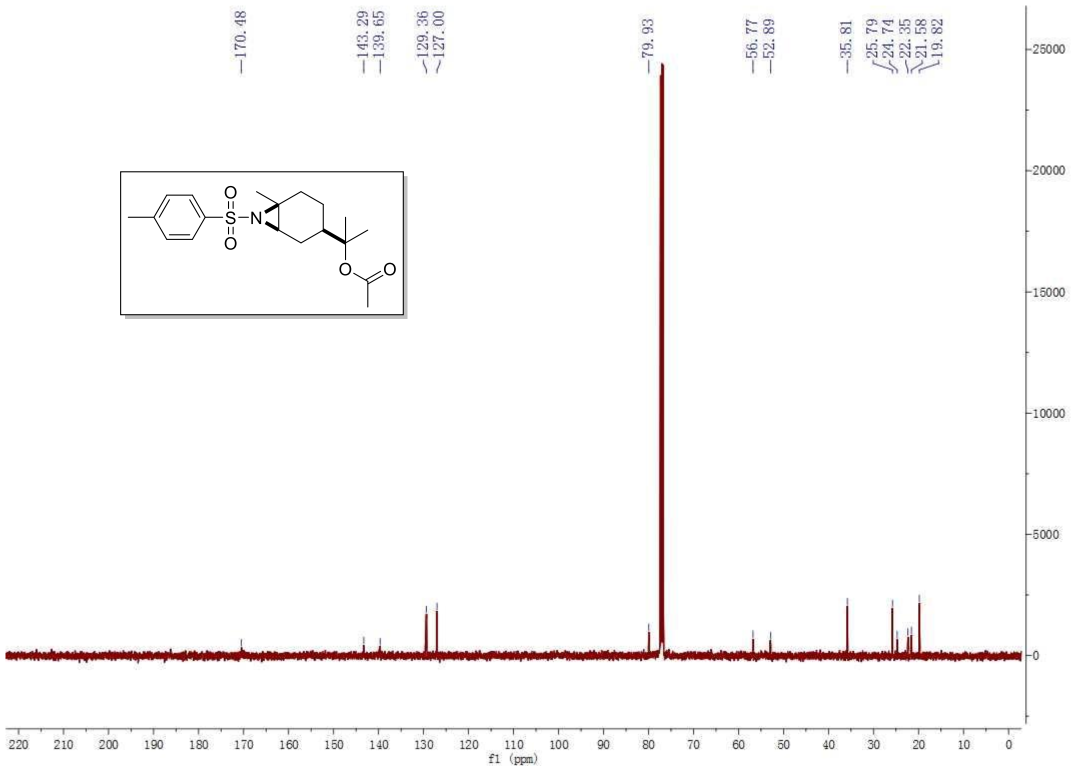

Supplementary Figure 48. ^{13}C NMR spectra for compound 26b

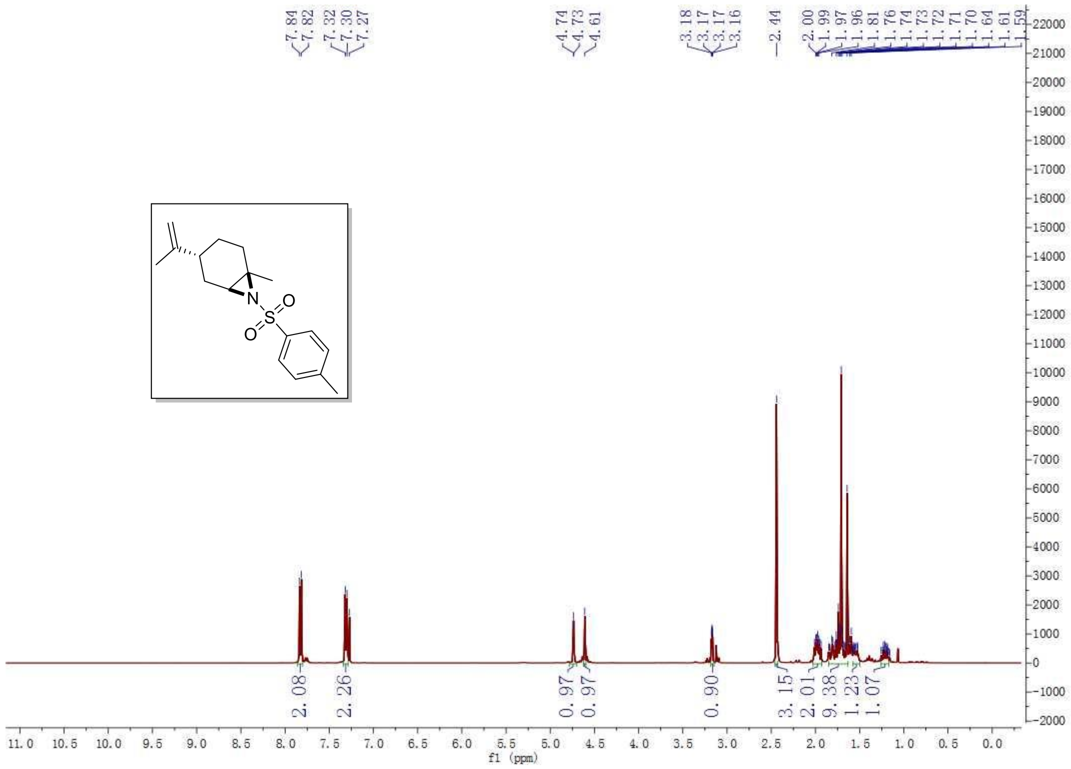

Supplementary Figure 49. ^{1}H NMR spectra for compound 27a

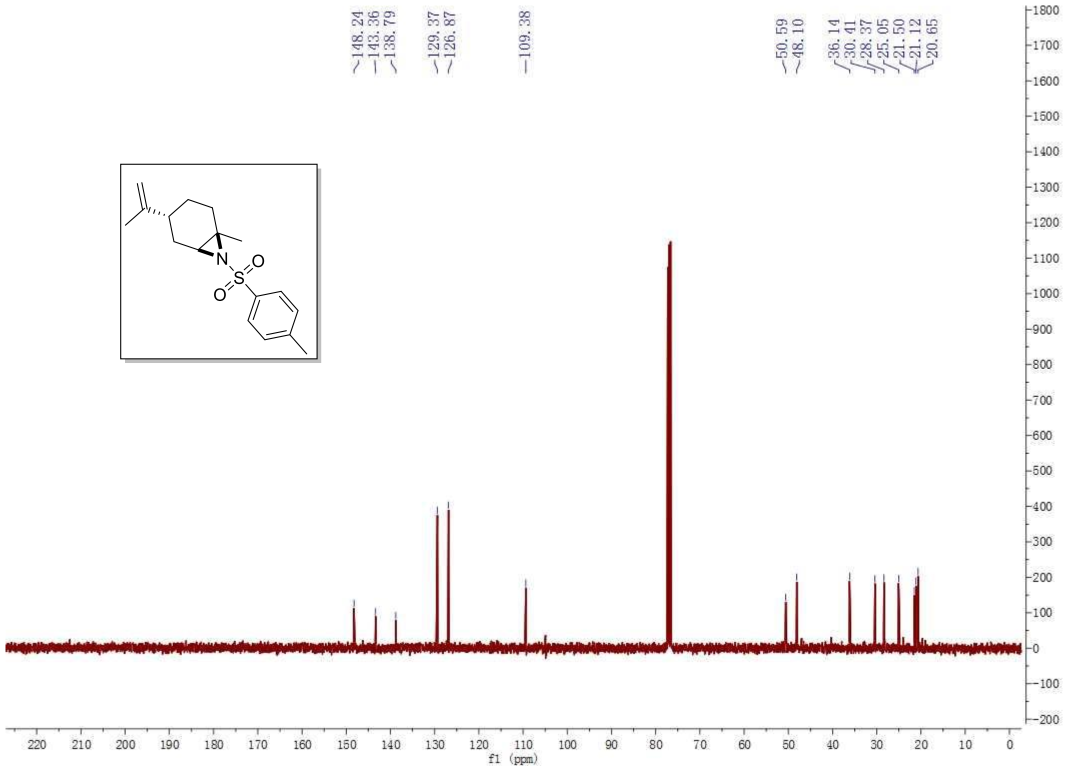

Supplementary Figure 50. ^{13}C NMR spectra for compound 27a

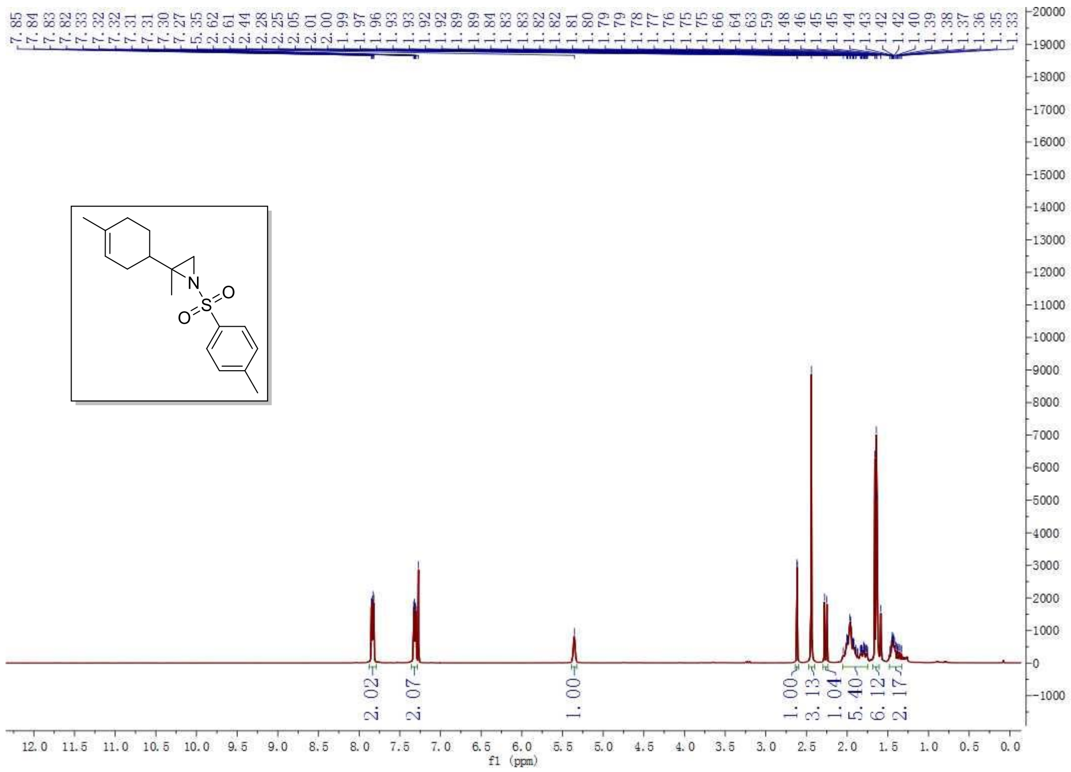

Supplementary Figure 51. ^{1}H NMR spectra for compound 27b

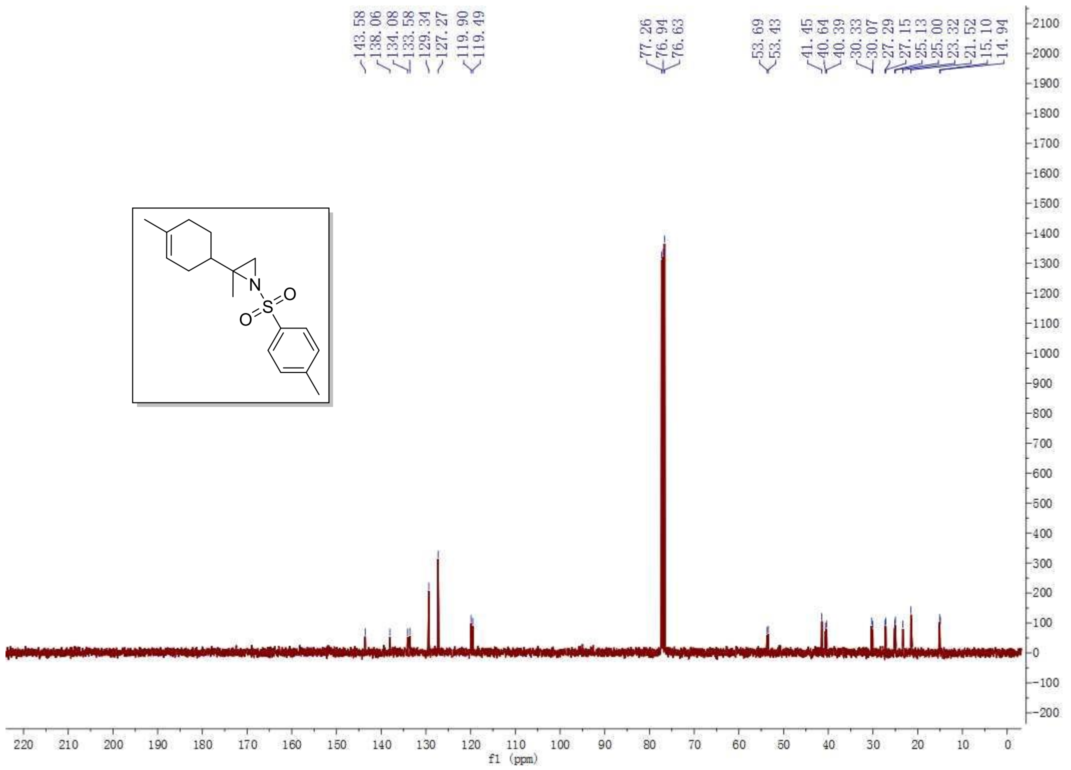

Supplementary Figure 52. ^{13}C NMR spectra for compound 27b

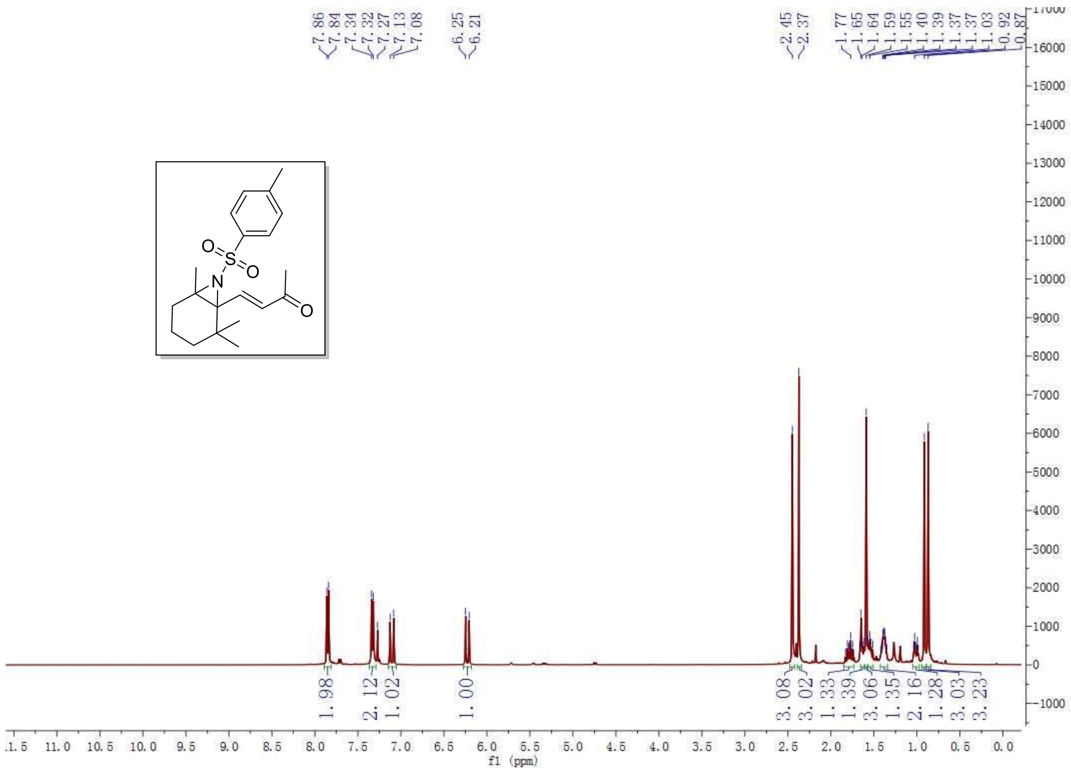

Supplementary Figure 53. ^{1}H NMR spectra for compound 42a

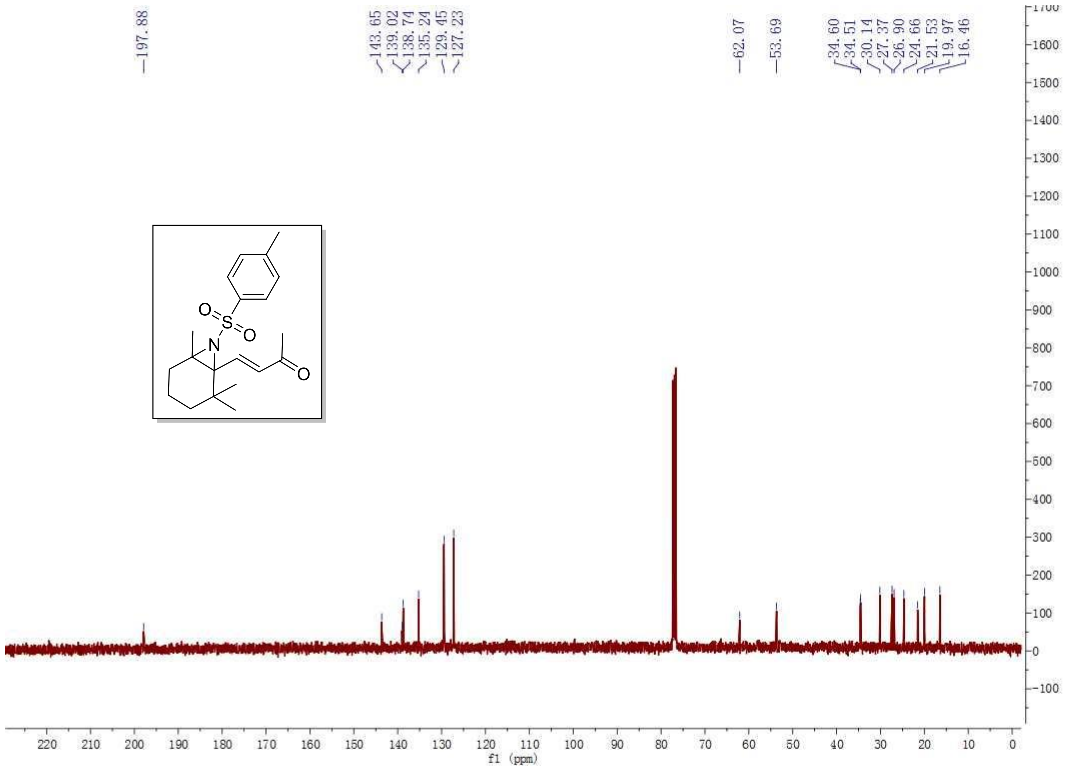

Supplementary Figure 54. ^{13}C NMR spectra for compound 42a

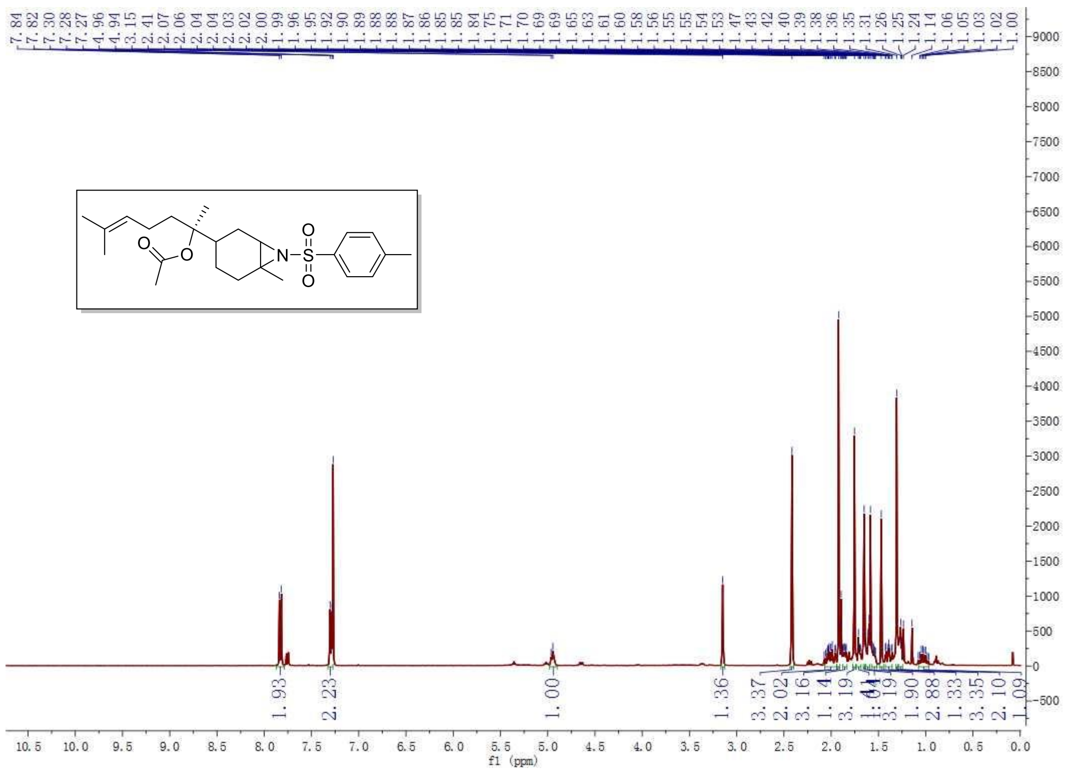

Supplementary Figure 55. ^{1}H NMR spectra for compound 43a

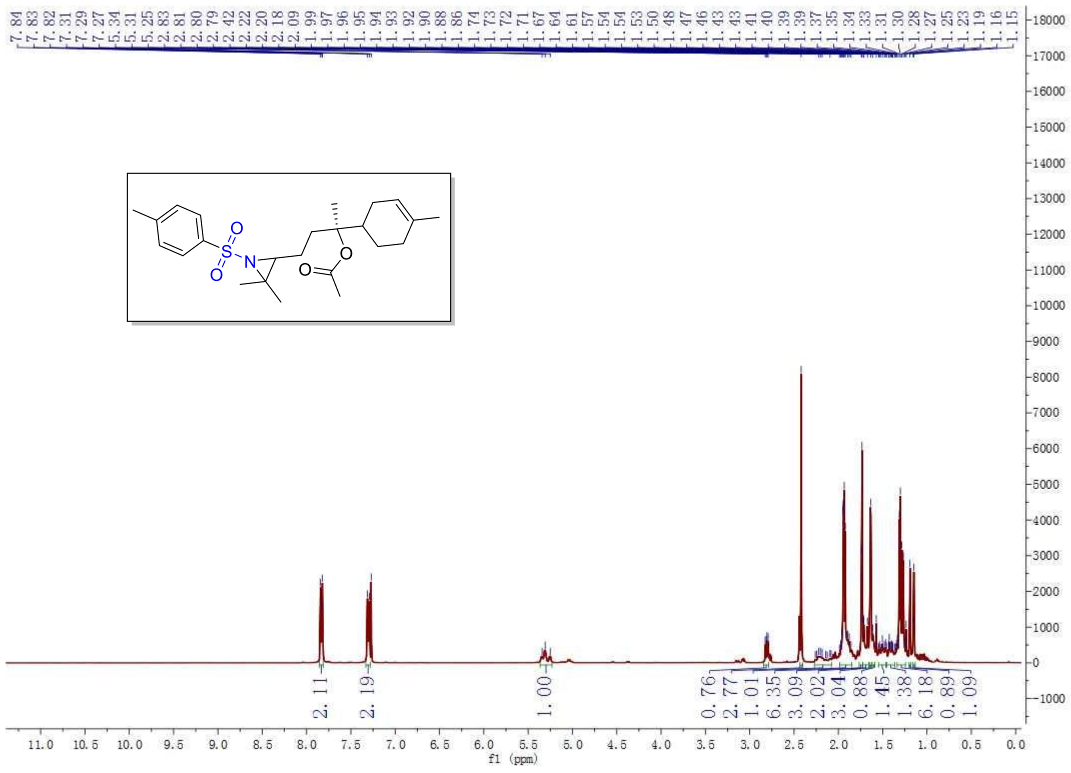

Supplementary Figure 56. ^{1}H NMR spectra for compound 43b

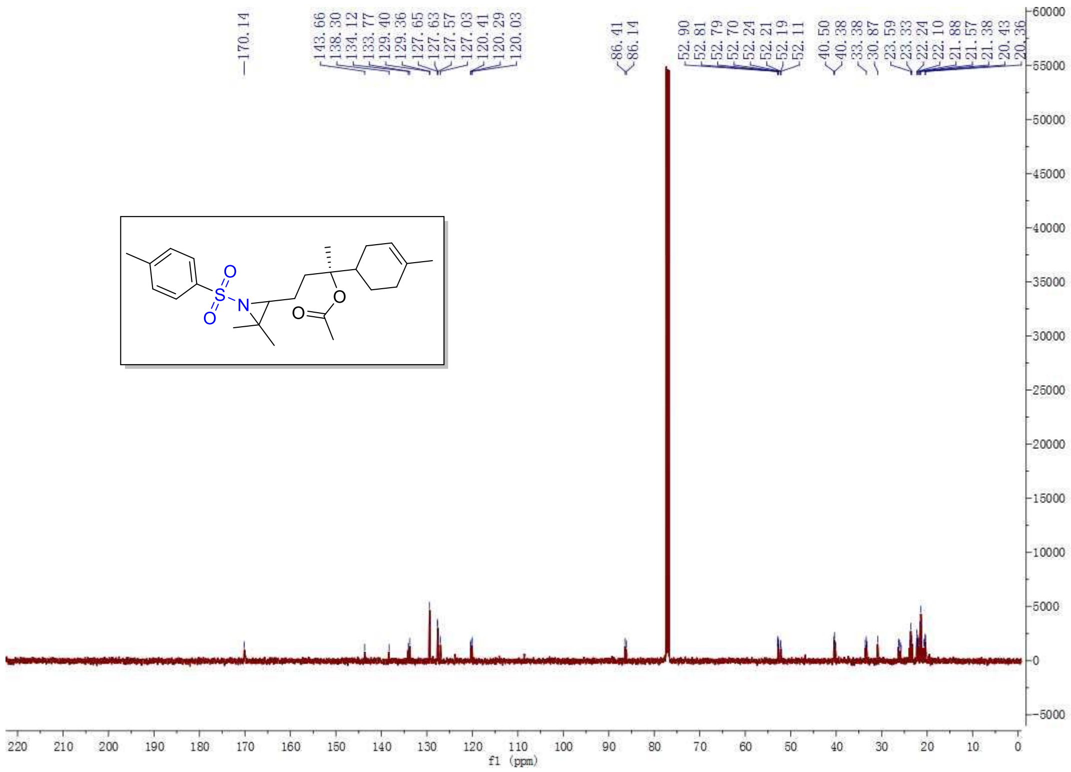

Supplementary Figure 57. ^{13}**C NMR spectra for compound 43b**

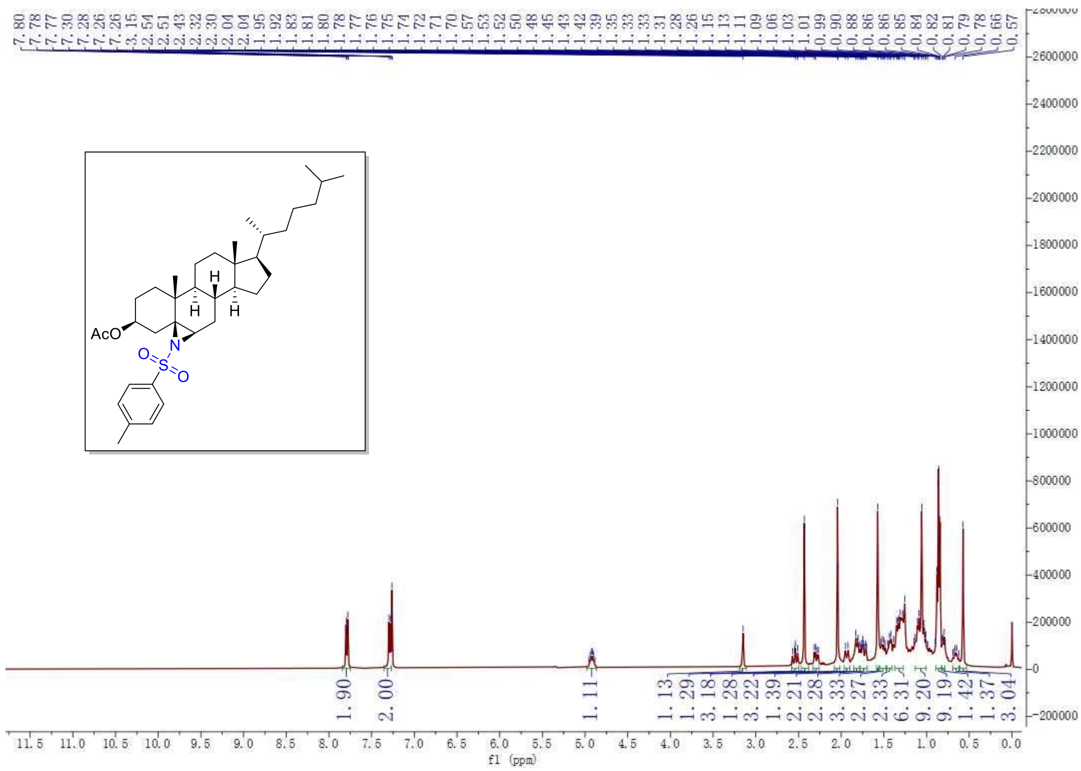

Supplementary Figure 58. ^{1}**H NMR spectra for compound 44a**

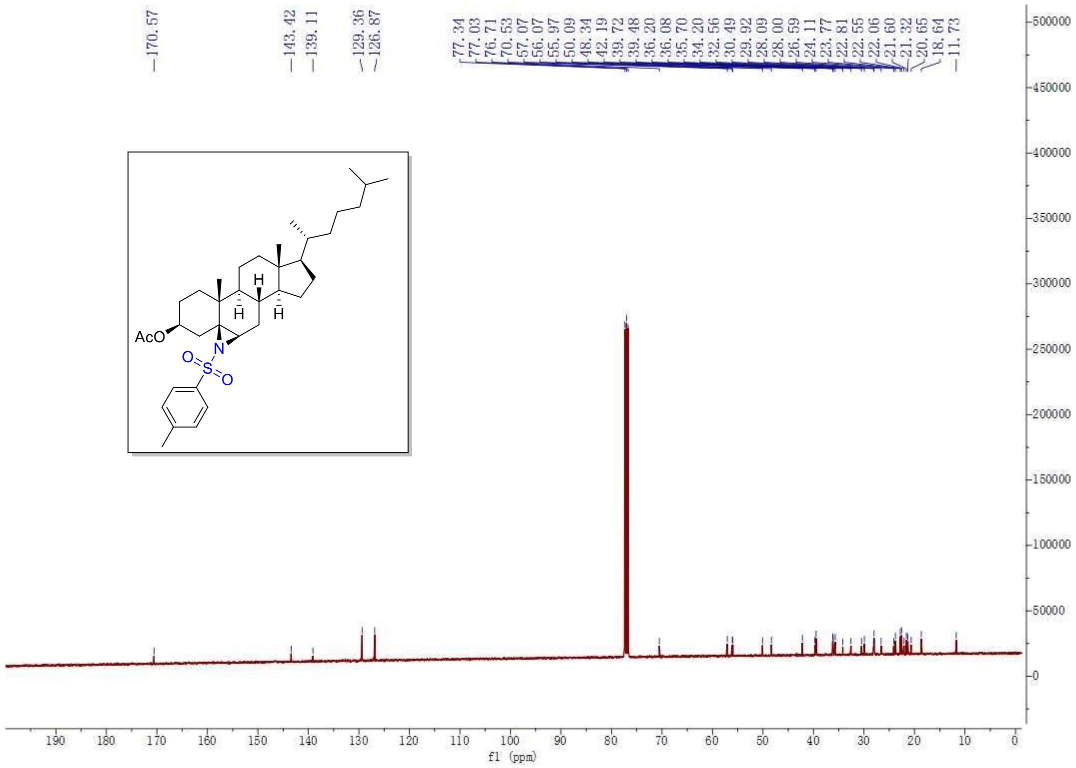

Supplementary Figure 59. ^{13}C NMR spectra for compound 44a

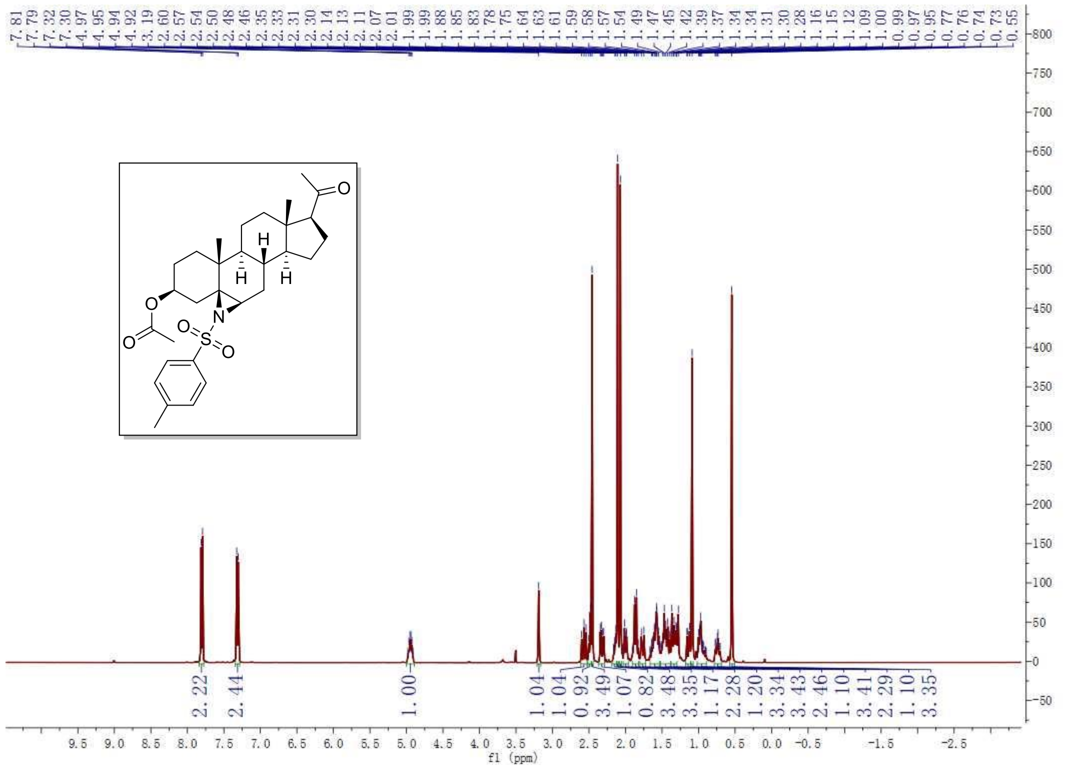

Supplementary Figure 60. ^{1}H NMR spectra for compound 45a

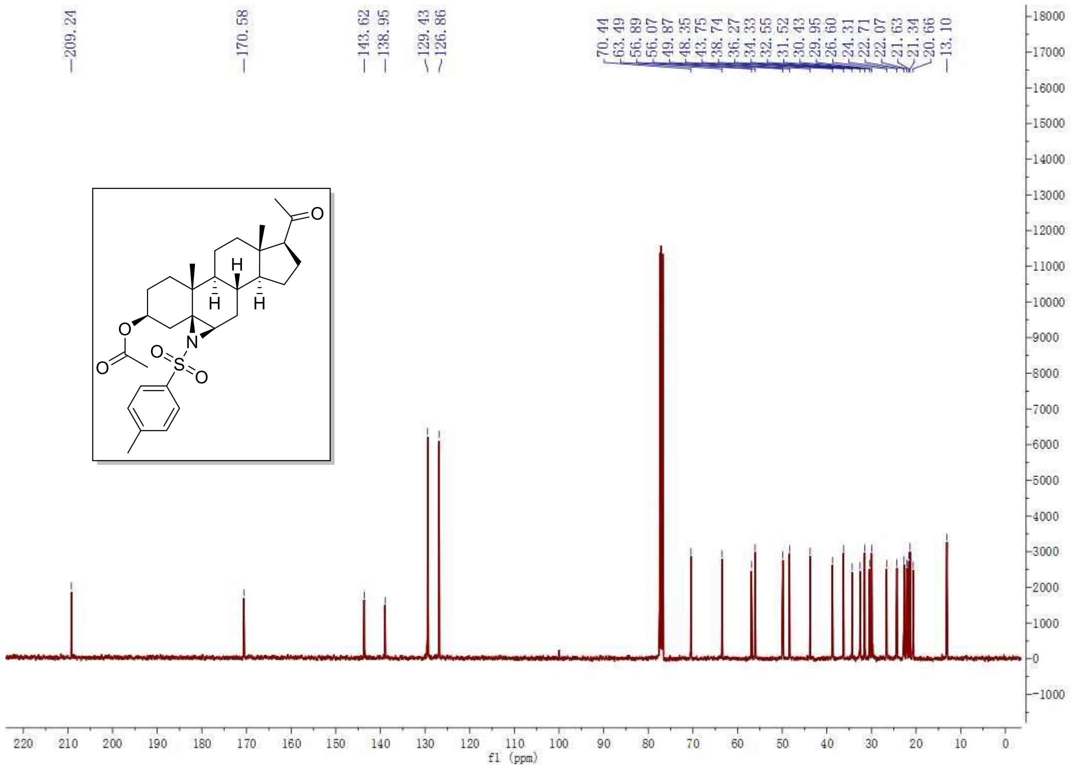

Supplementary Figure 61. ^{13}C NMR spectra for compound 45a

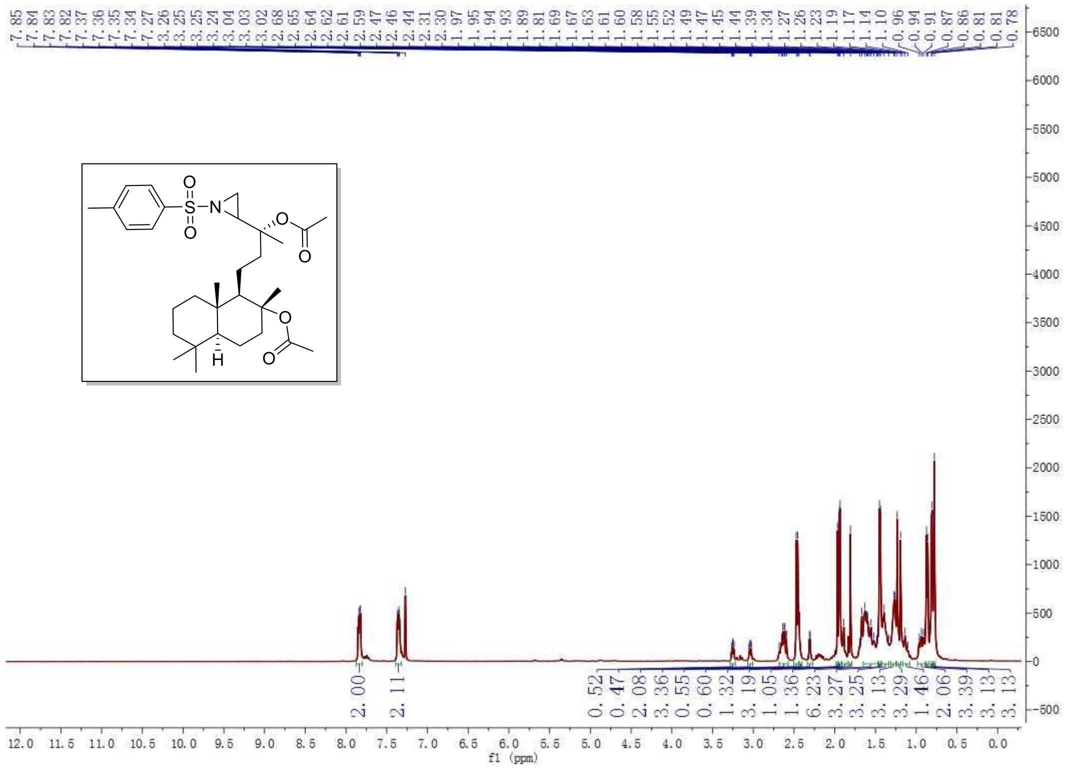

Supplementary Figure 62. ^{1}H NMR spectra for compound 46a

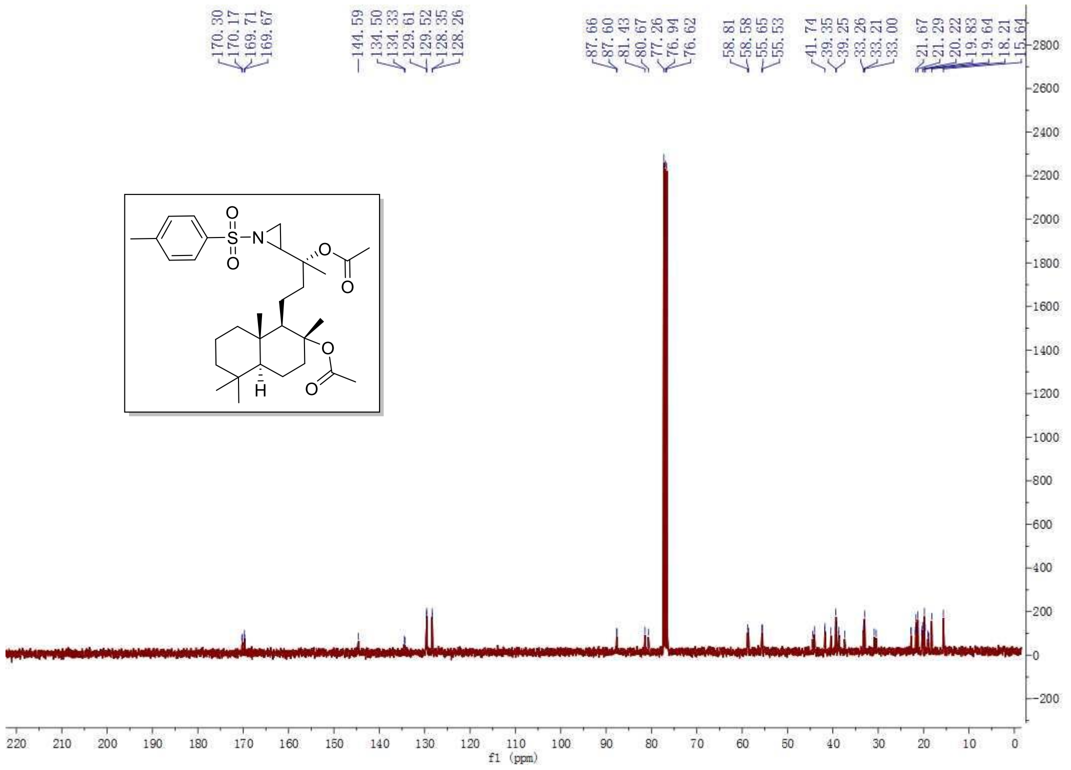

Supplementary Figure 63. ^{13}C NMR spectra for compound 46a

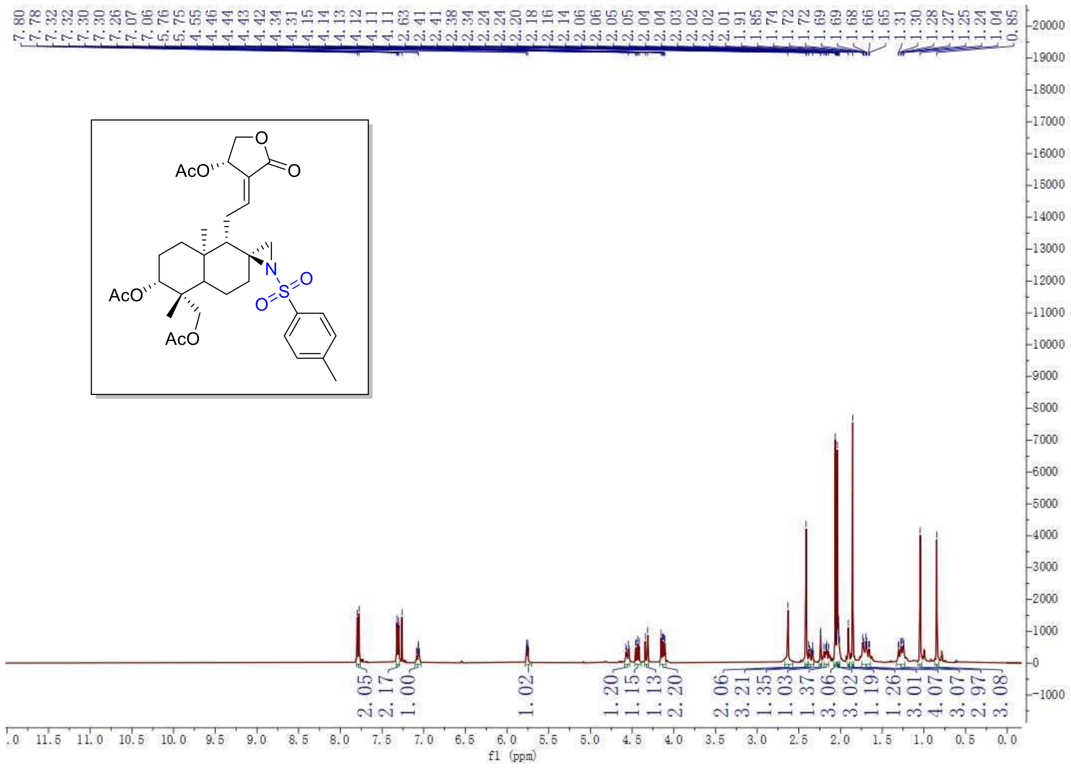

Supplementary Figure 64. ^{1}H NMR spectra for compound 47a

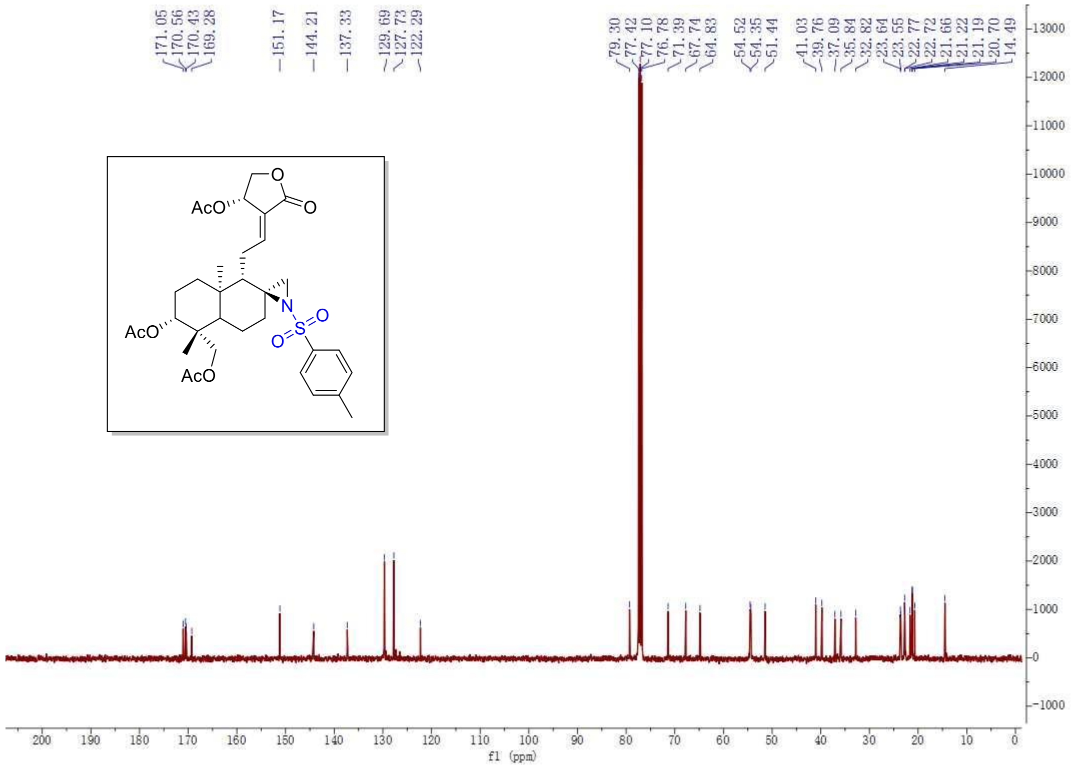

Supplementary Figure 65. ^{13}C NMR spectra for compound 47a

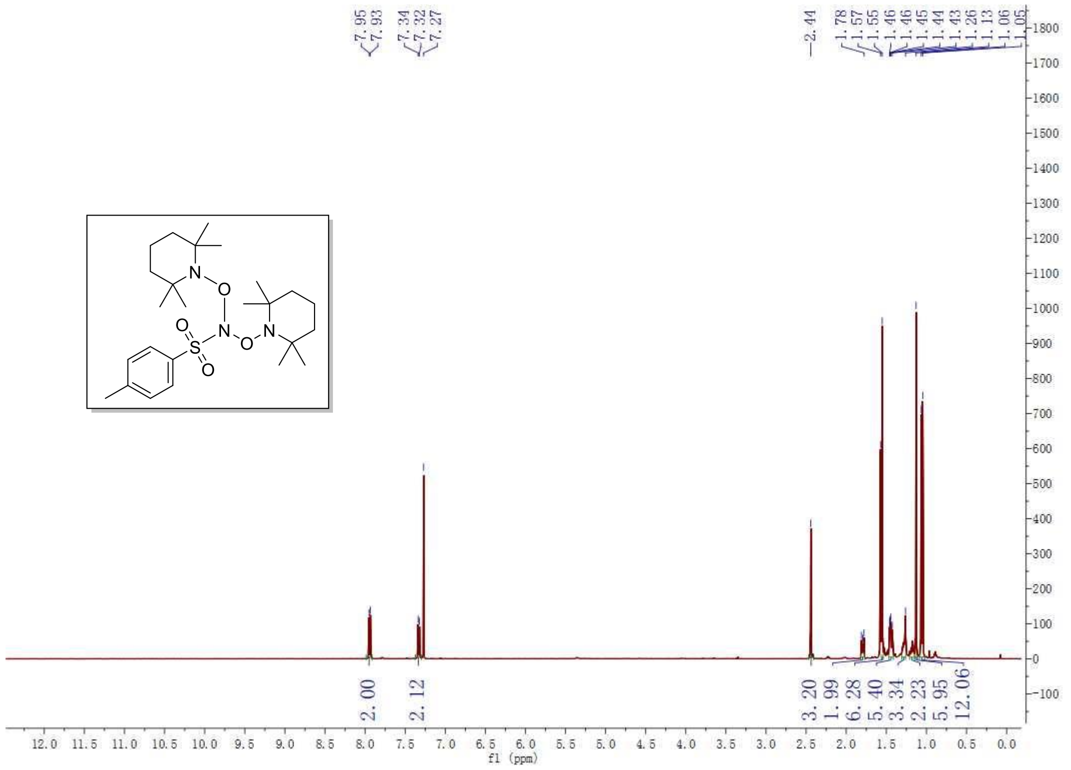

Supplementary Figure 66. ^{1}H NMR spectra for compound 48

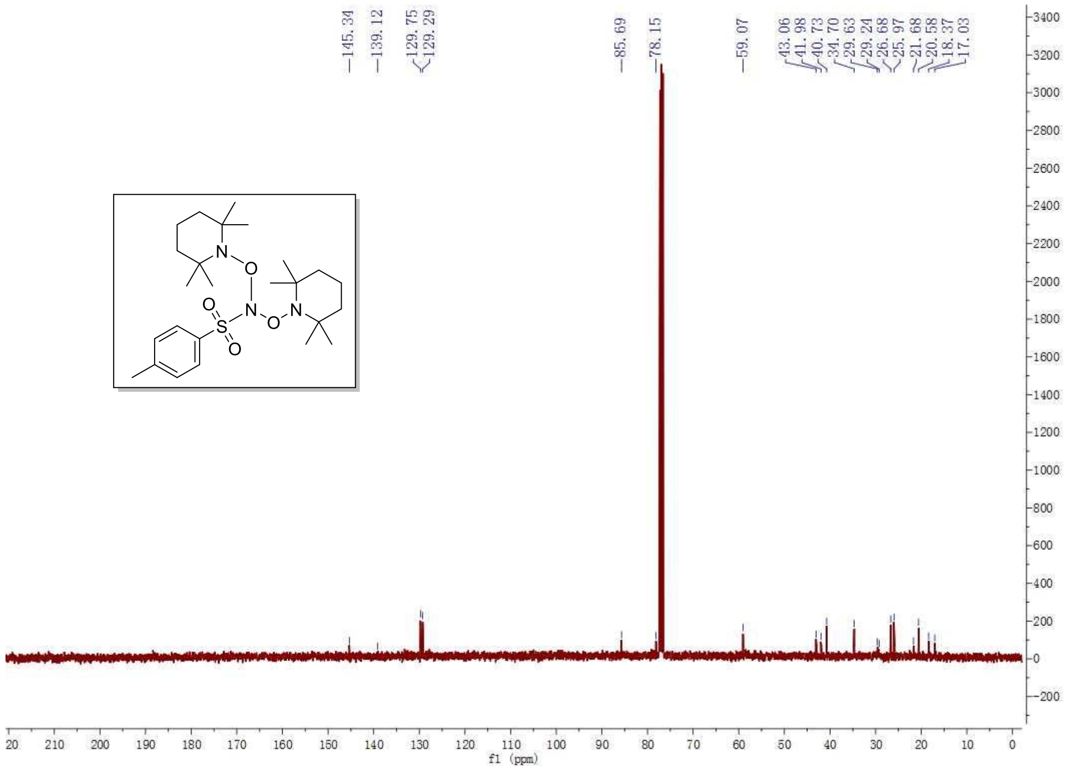

Supplementary Figure 67. ^{13}C NMR spectra for compound 48

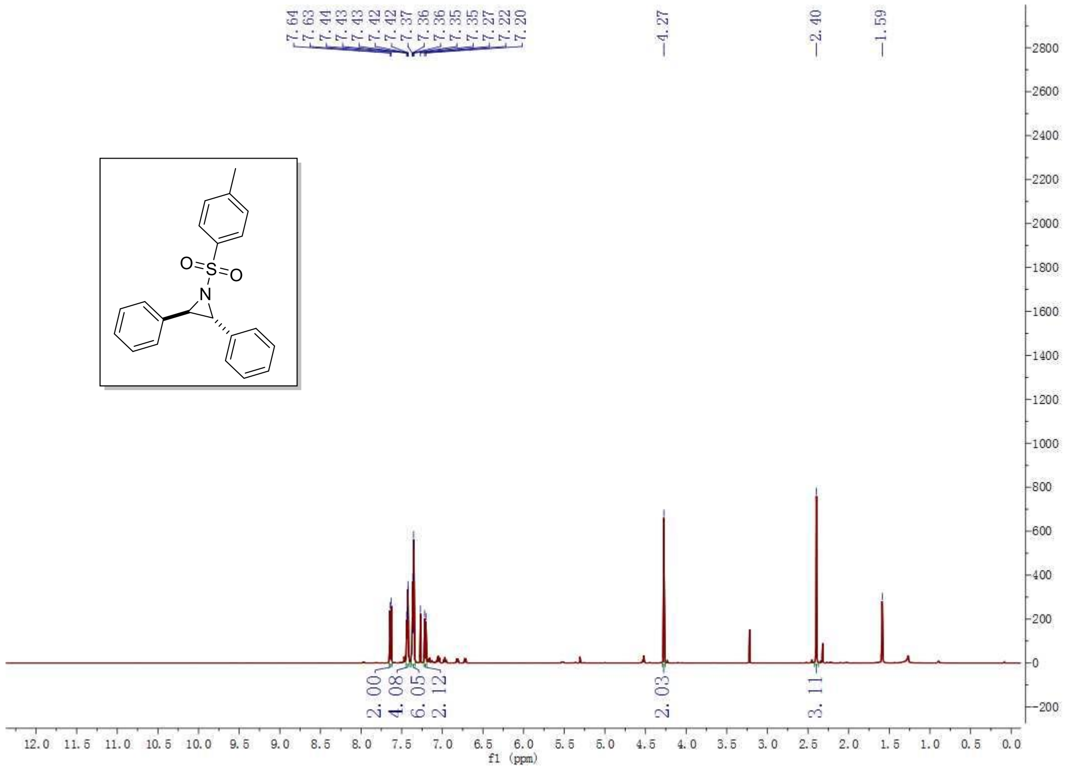

Supplementary Figure 68. ^{1}H NMR spectra for compound 50 (*anti* isomer)

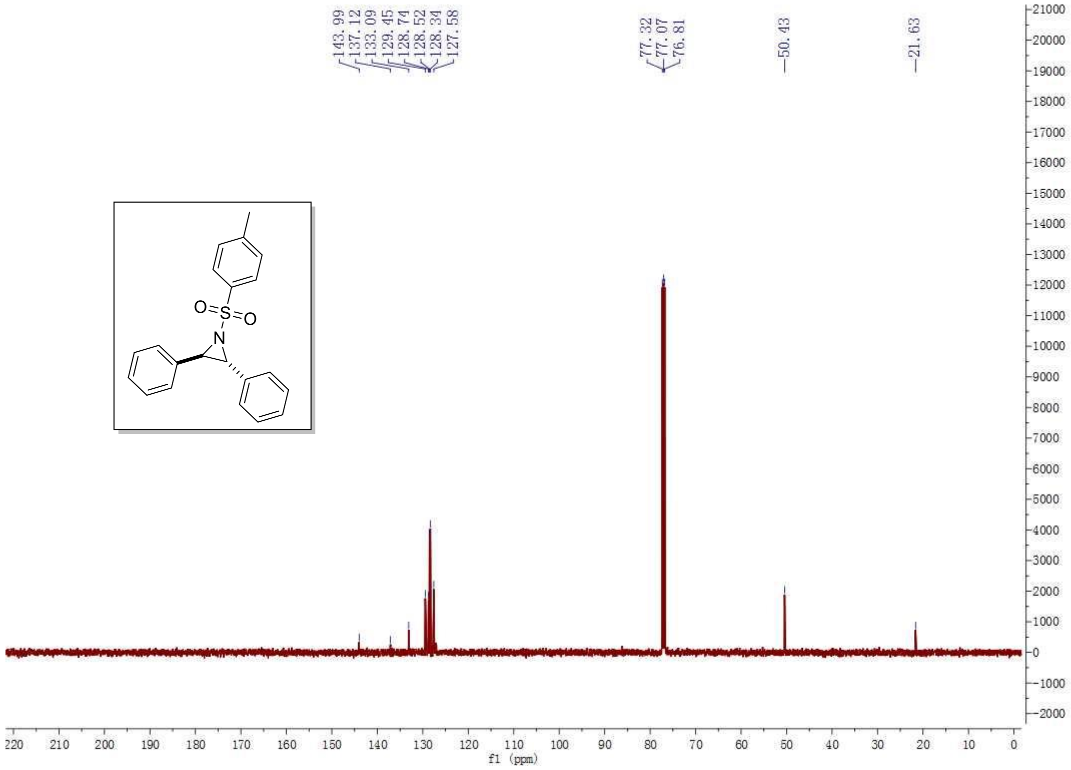

Supplementary Figure 69. **^{1}H NMR spectra for compound 50 (*anti* isomer)**

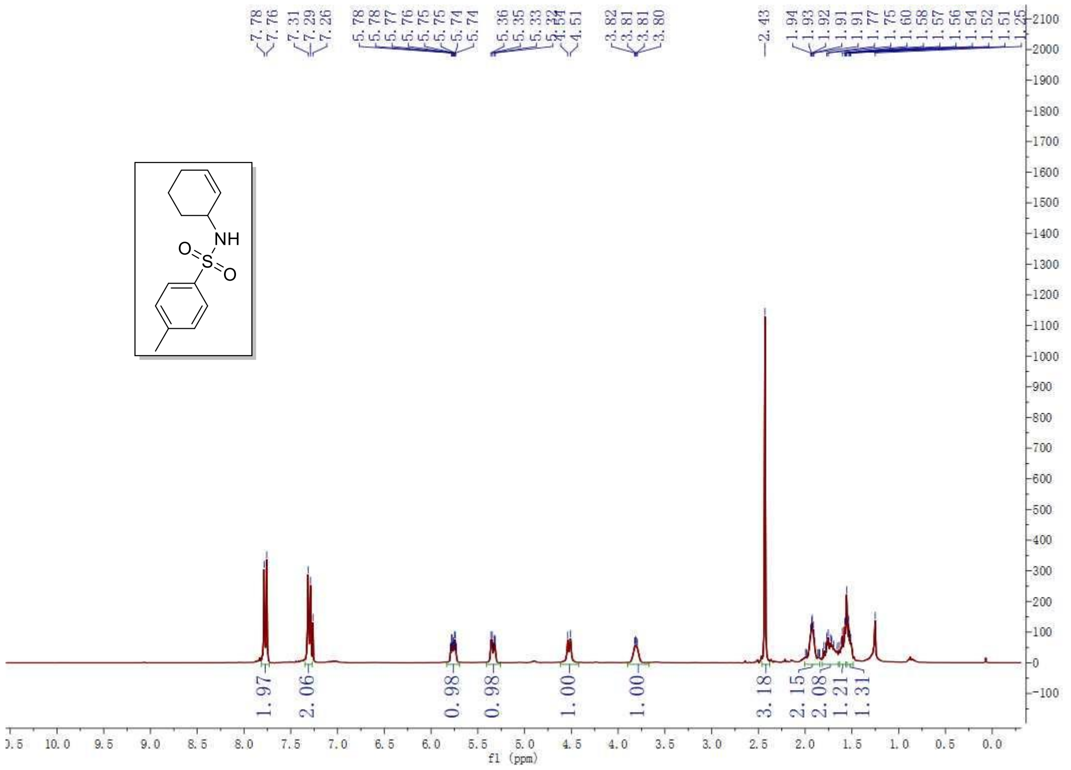

Supplementary Figure 70. **^{1}H NMR spectra for compound 4**

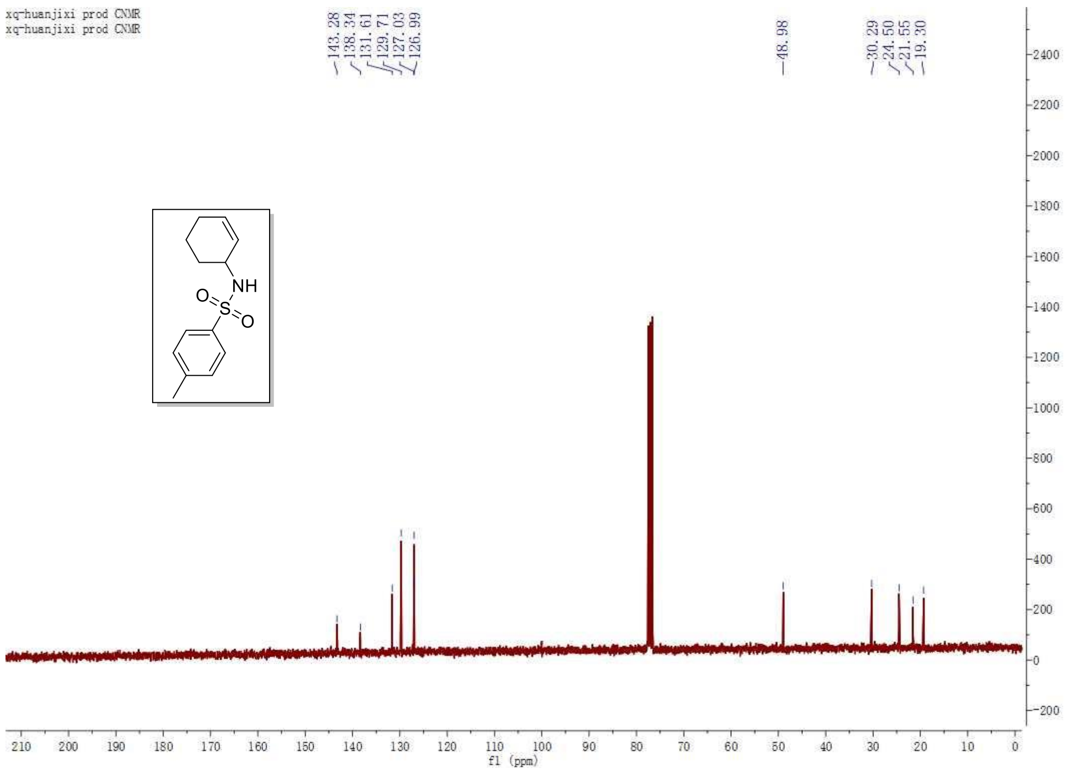

Supplementary Figure 71. ^{13}**C NMR spectra for compound 4**

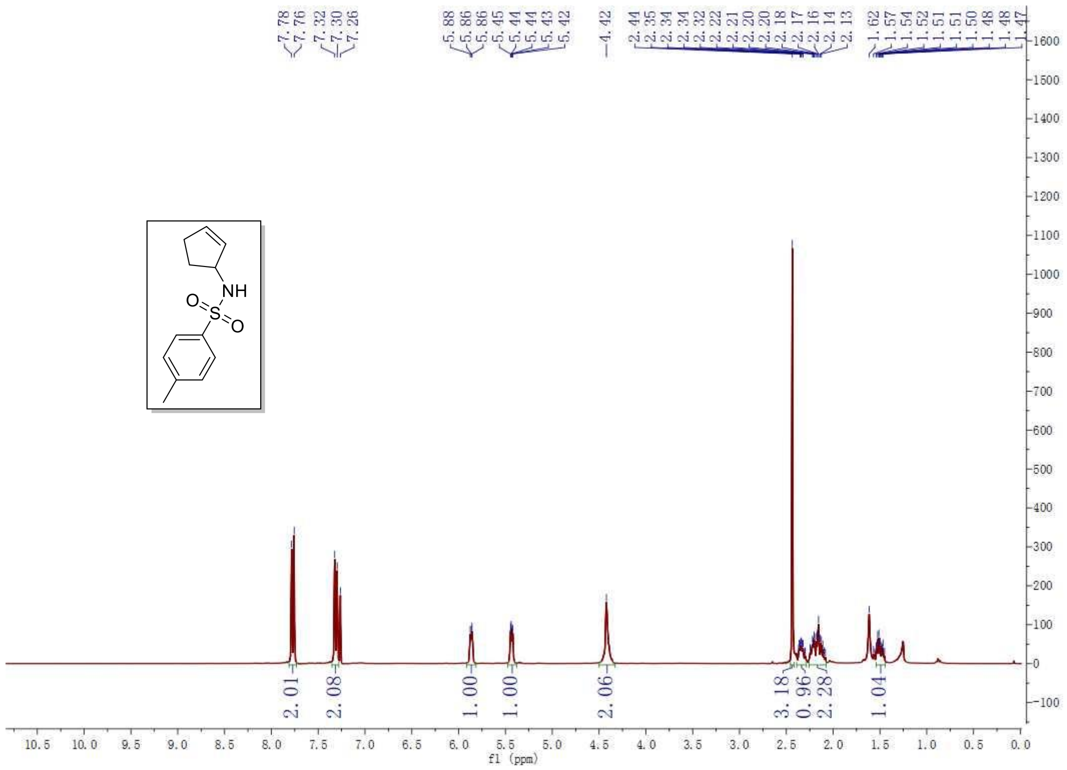

Supplementary Figure 72. ^{1}**H NMR spectra for compound 28**

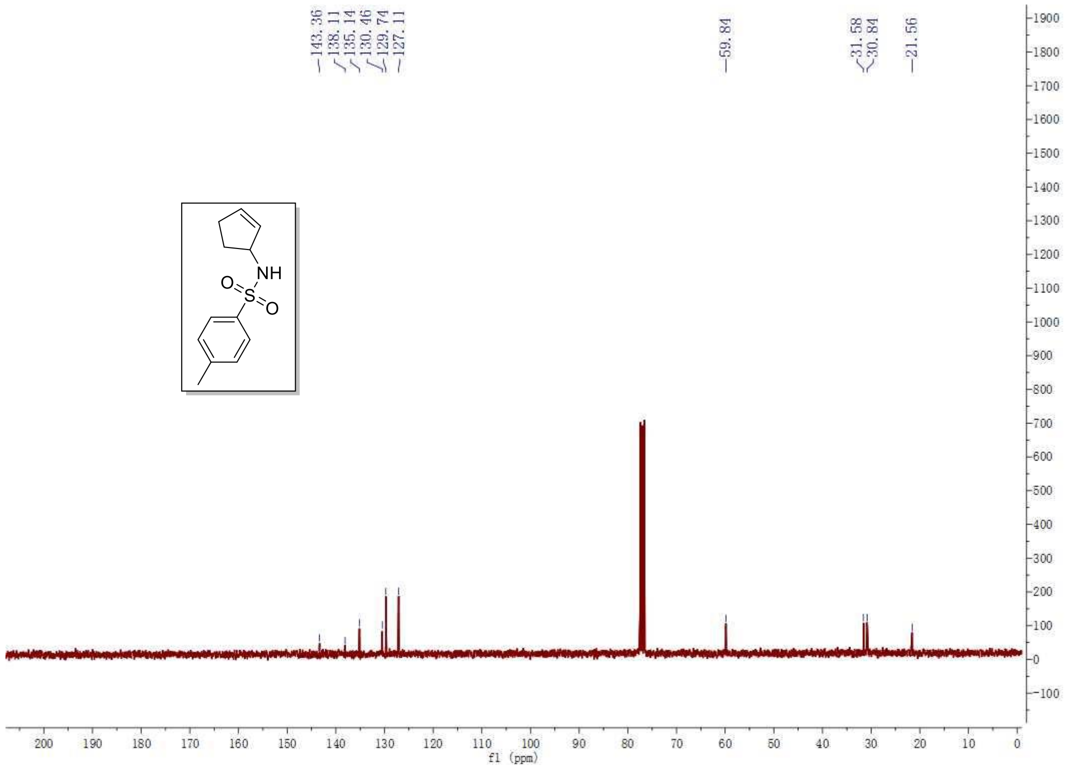

Supplementary Figure 73. ^{13}C NMR spectra for compound 28

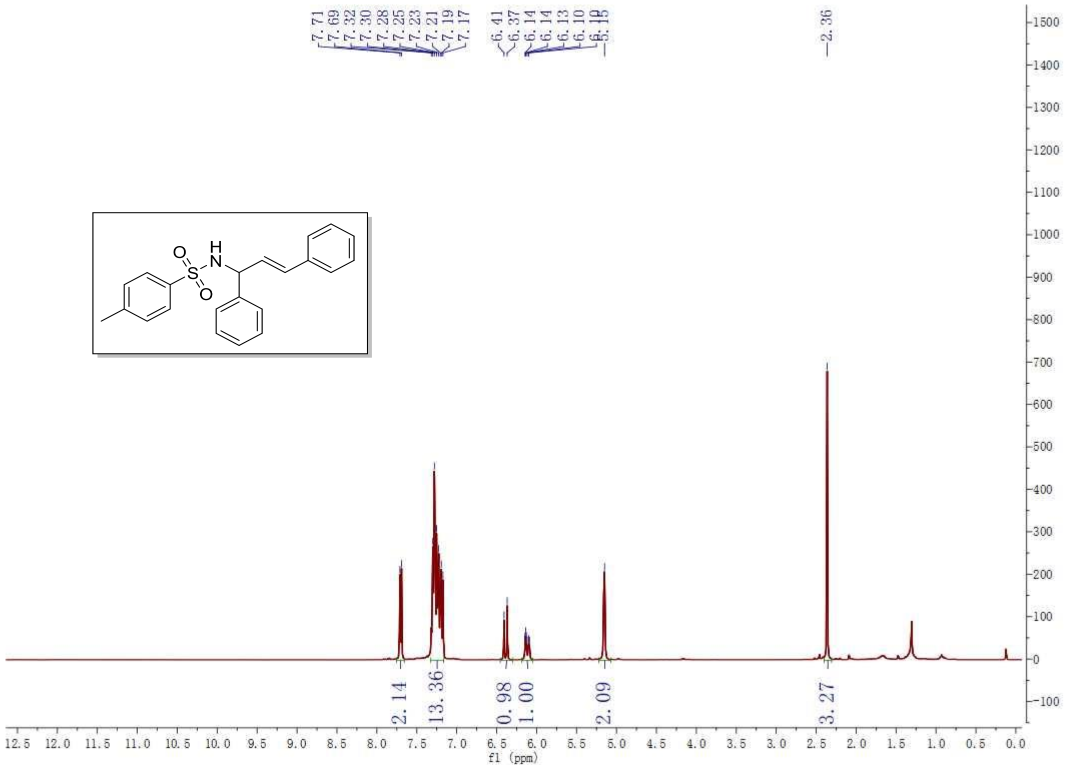

Supplementary Figure 74. ^{1}H NMR spectra for compound 30

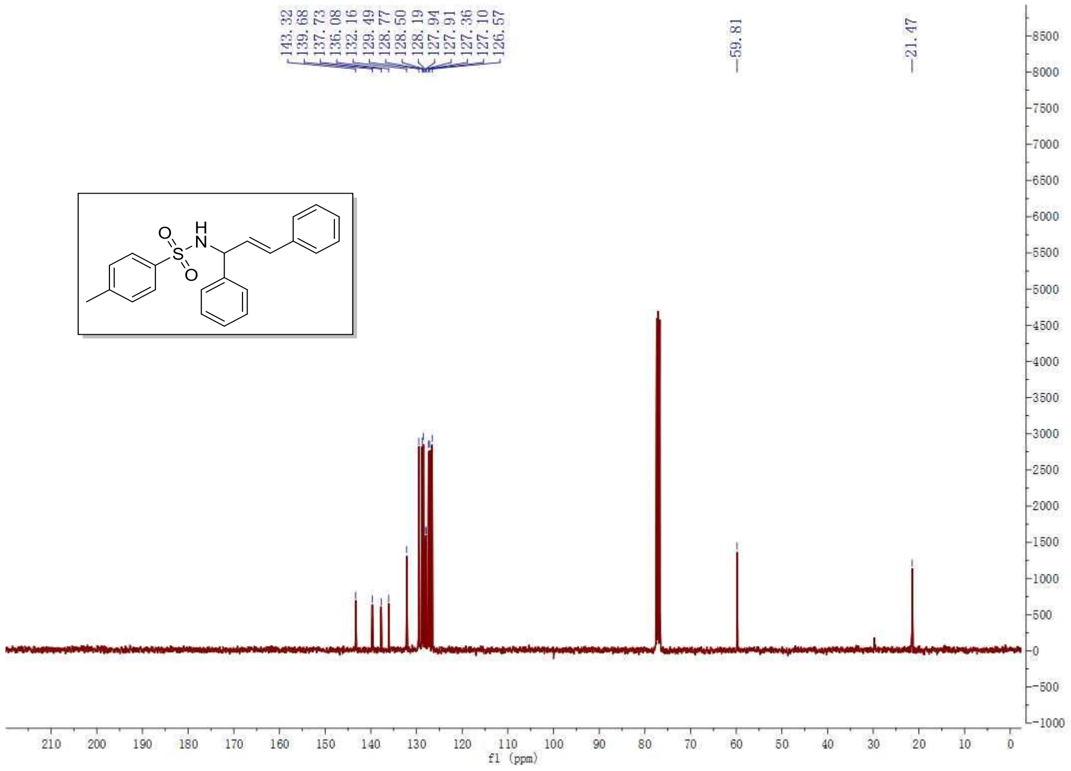

Supplementary Figure 75. ^{13}**C NMR spectra for compound 30**

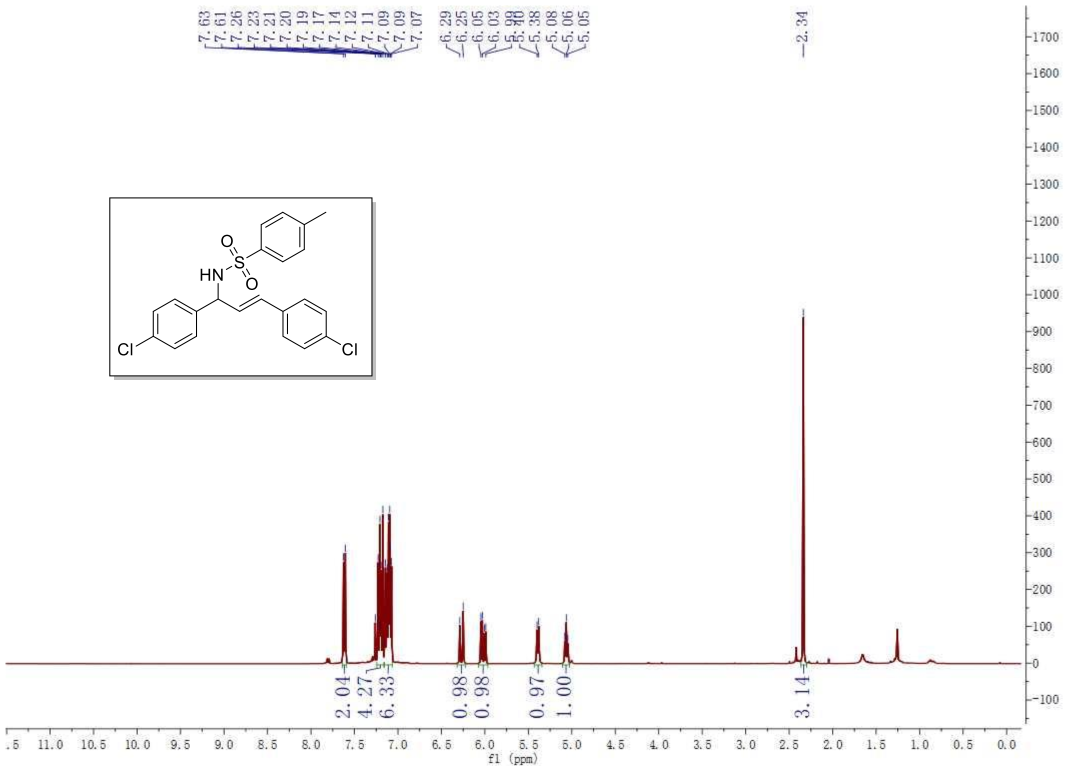

Supplementary Figure 76. ^{1}**H NMR spectra for compound 31**

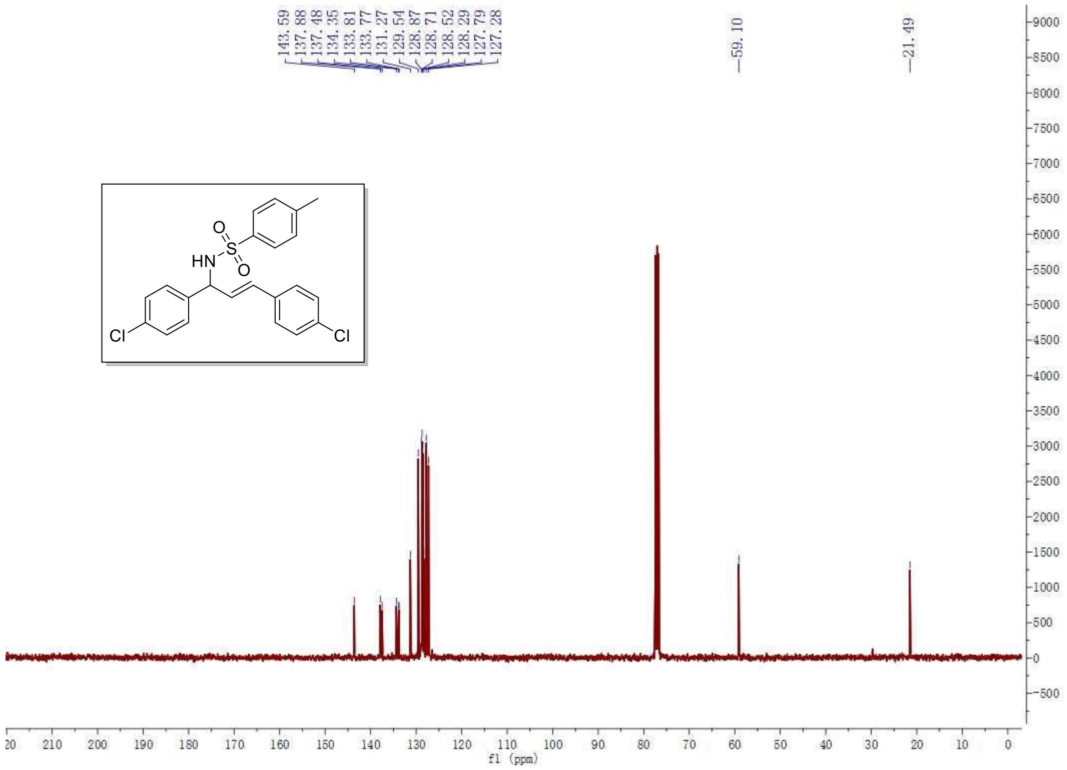

Supplementary Figure 77. ^{13}C NMR spectra for compound 31

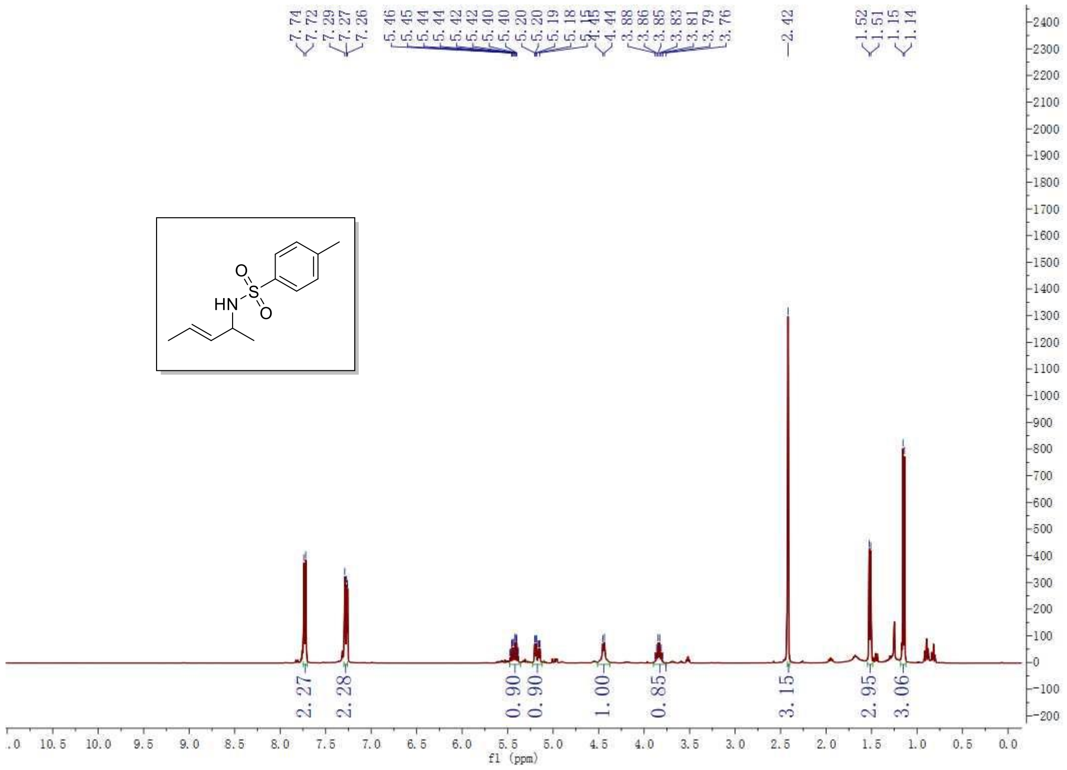

Supplementary Figure 78. ^{1}H NMR spectra for compound 32

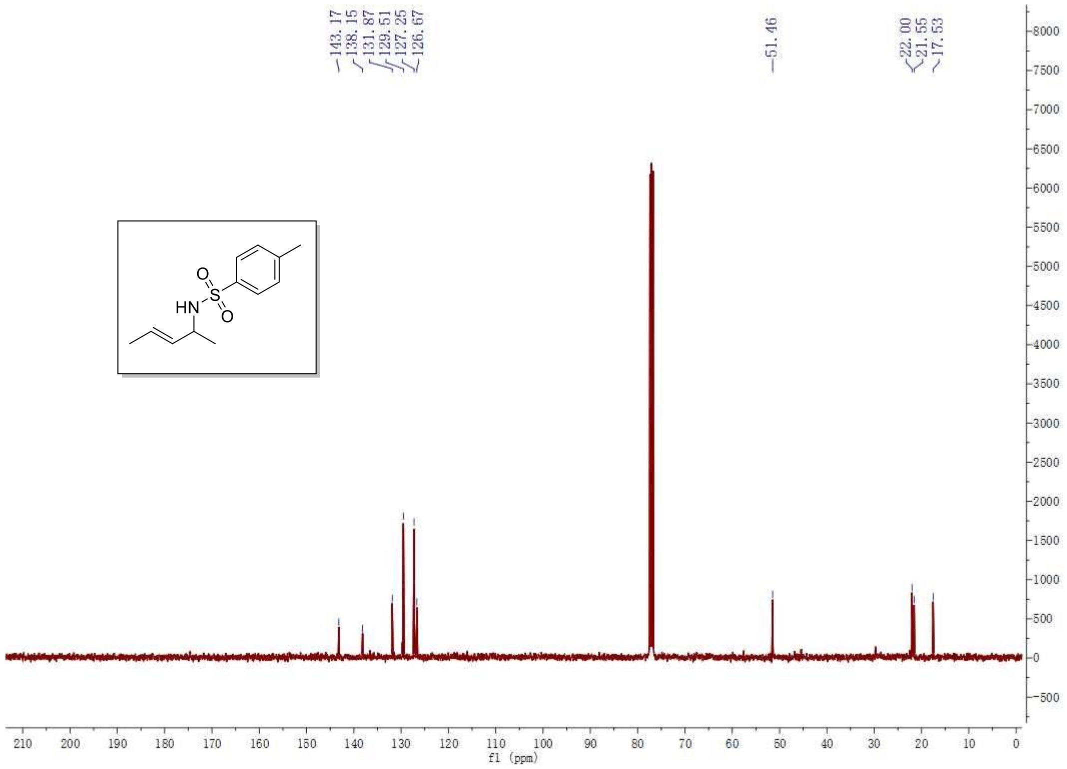

Supplementary Figure 79. ^{13}C NMR spectra for compound 32

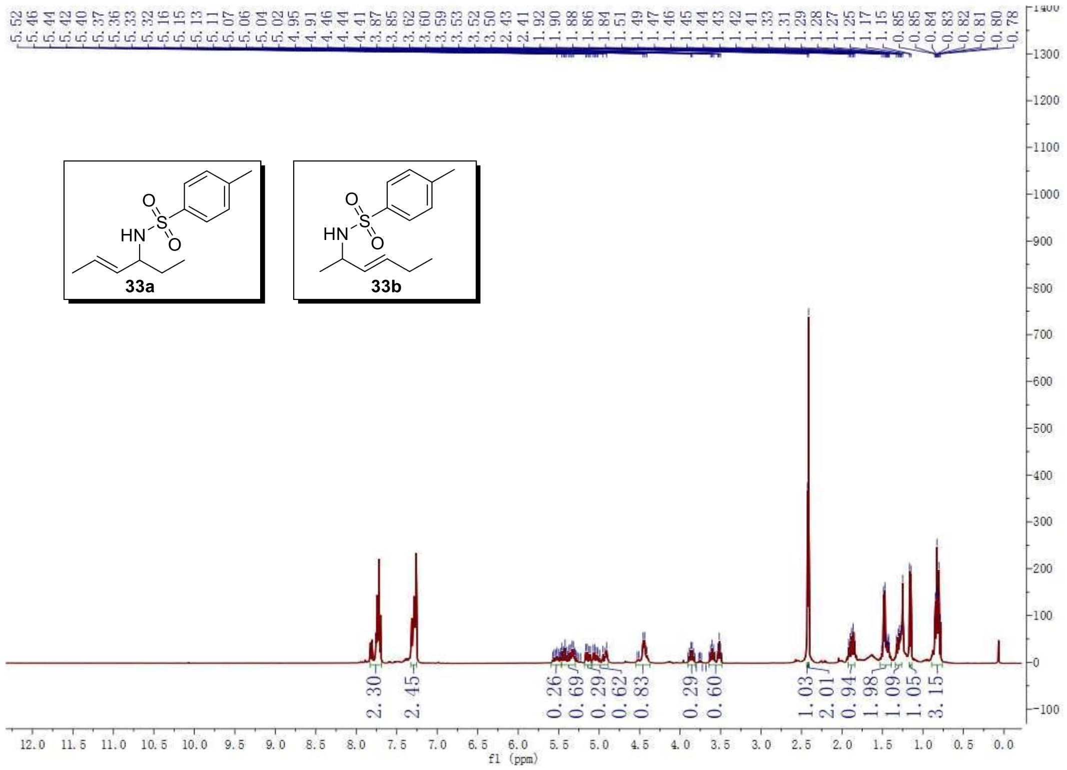

Supplementary Figure 80. ^{1}H NMR spectra for the mixture of 33a and 33b

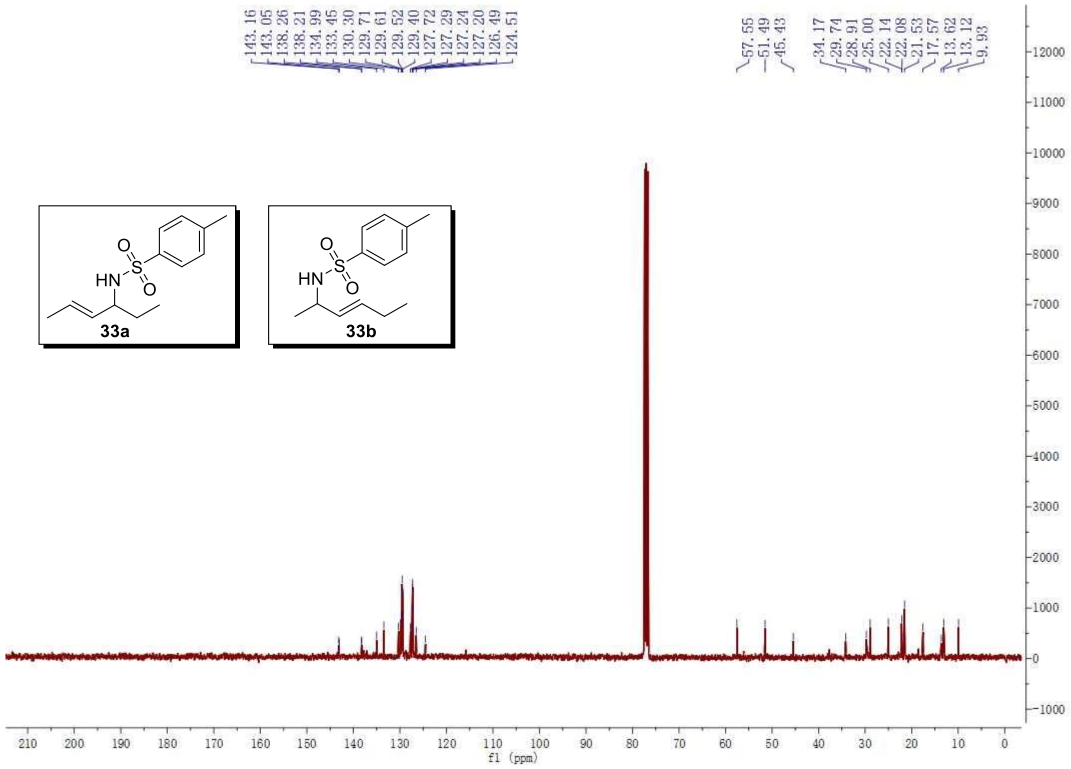

Supplementary Figure 81. ^{13}C NMR spectra for the mixture of 33a and 33b

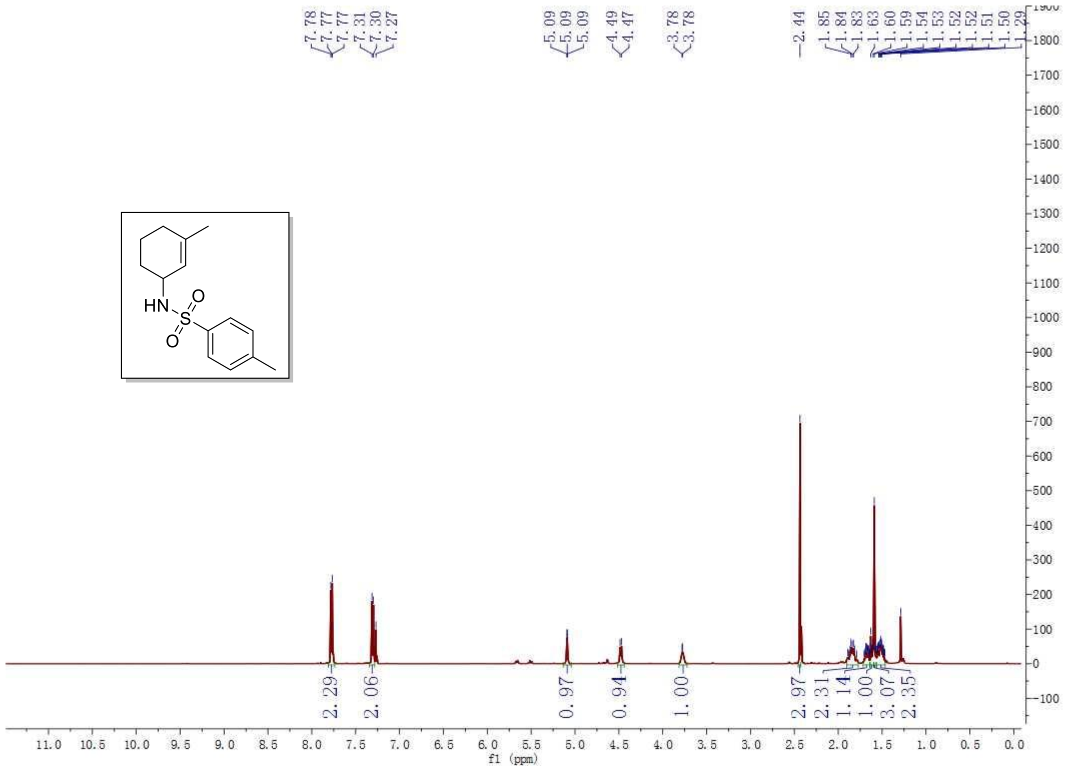

Supplementary Figure 82. ^{1}H NMR spectra for compound 34a

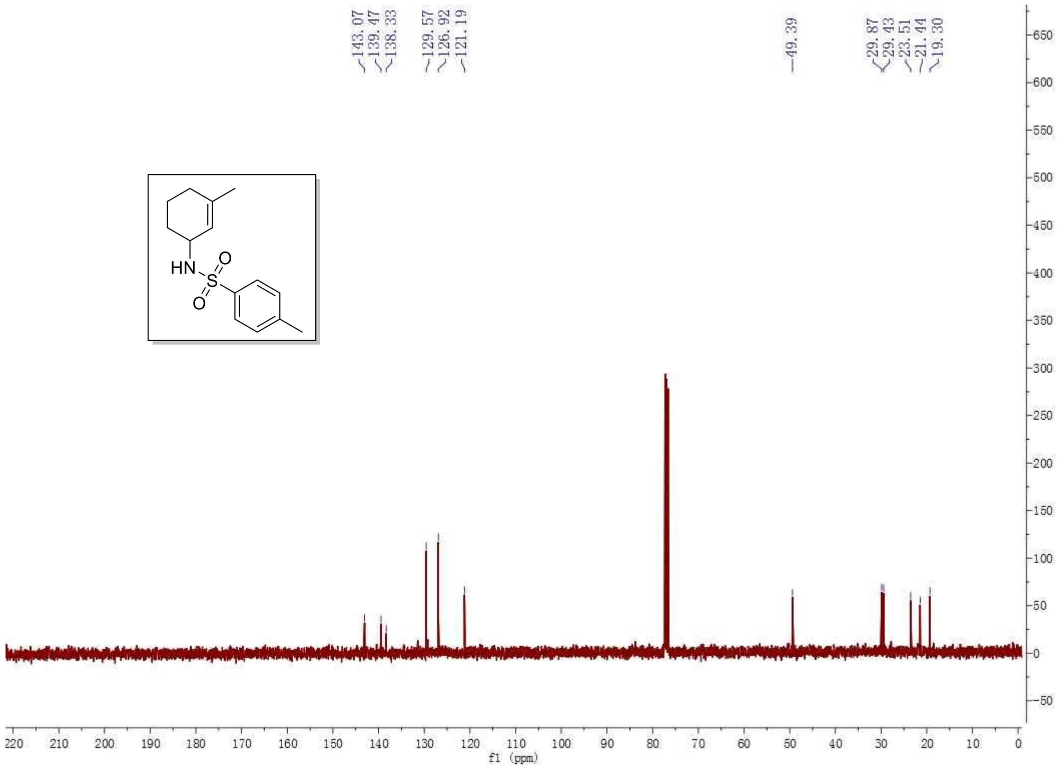

Supplementary Figure 83. **^{13}C NMR spectra for compound 34a**

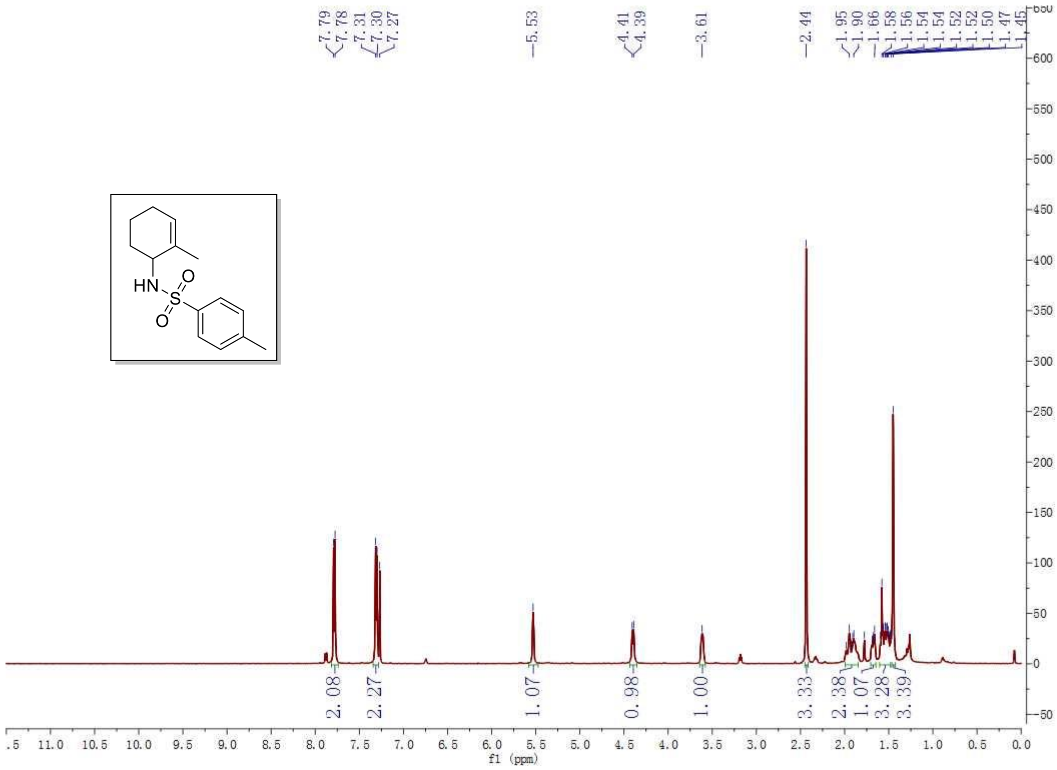

Supplementary Figure 84. **^{1}H NMR spectra for compound 34b**

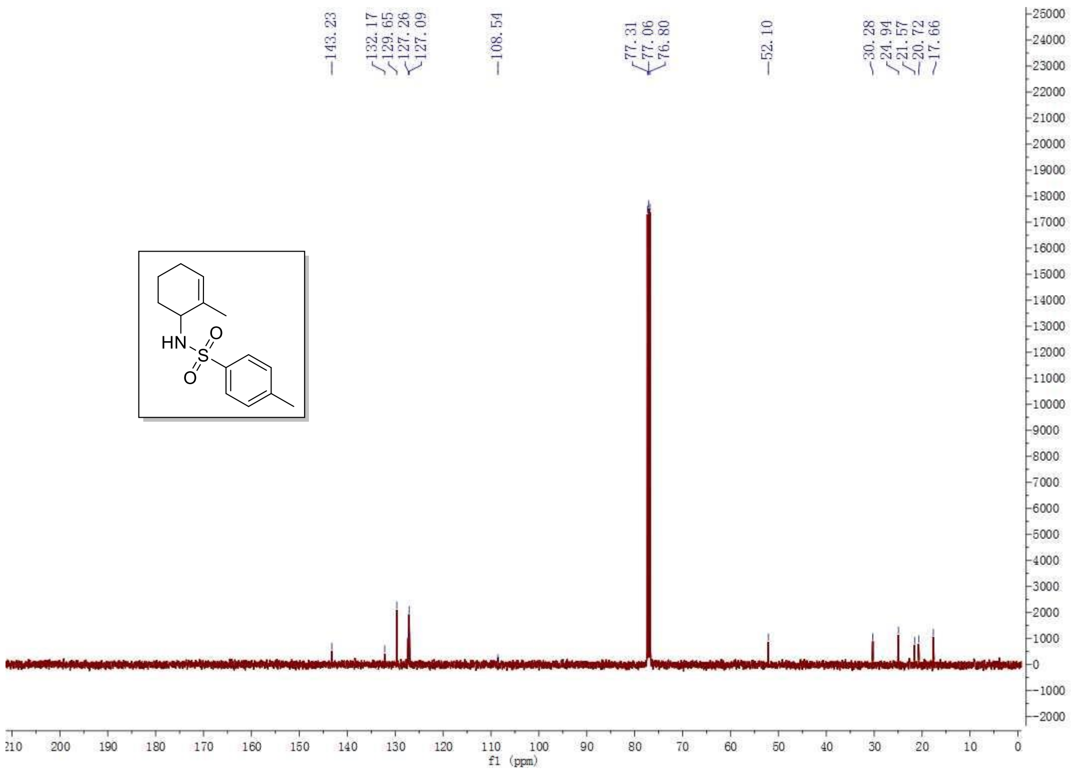

Supplementary Figure 85. ^{13}C NMR spectra for compound 34b

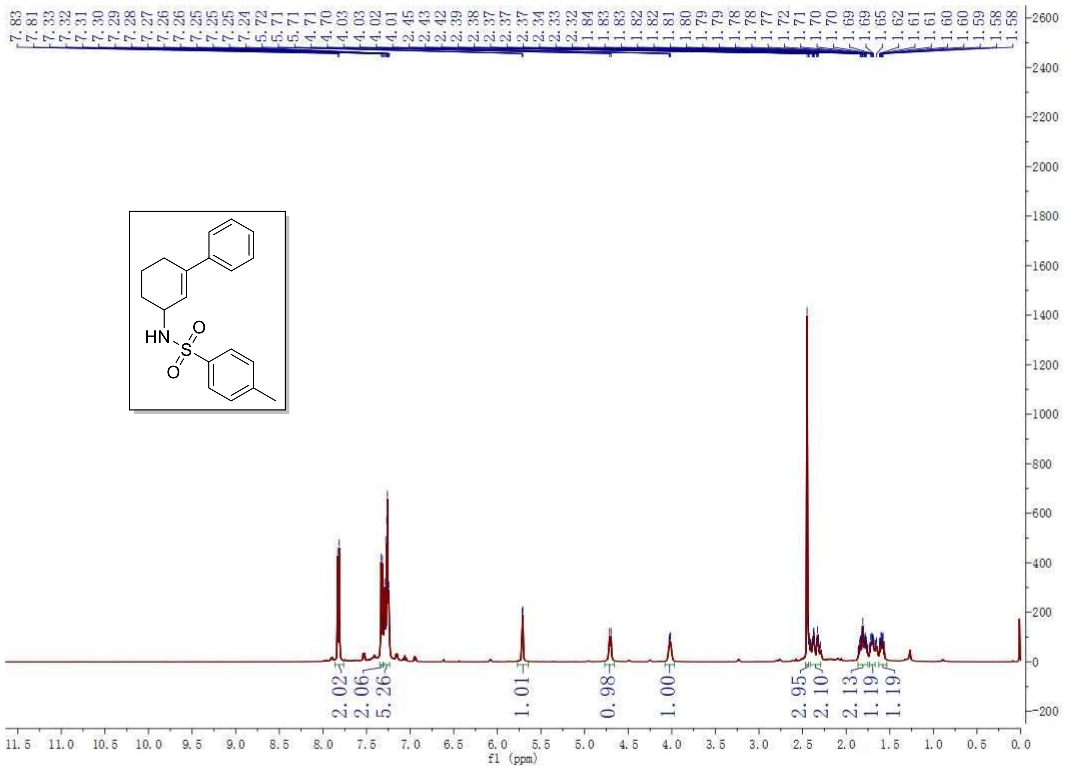

Supplementary Figure 86. ^{1}H NMR spectra for compound 35a

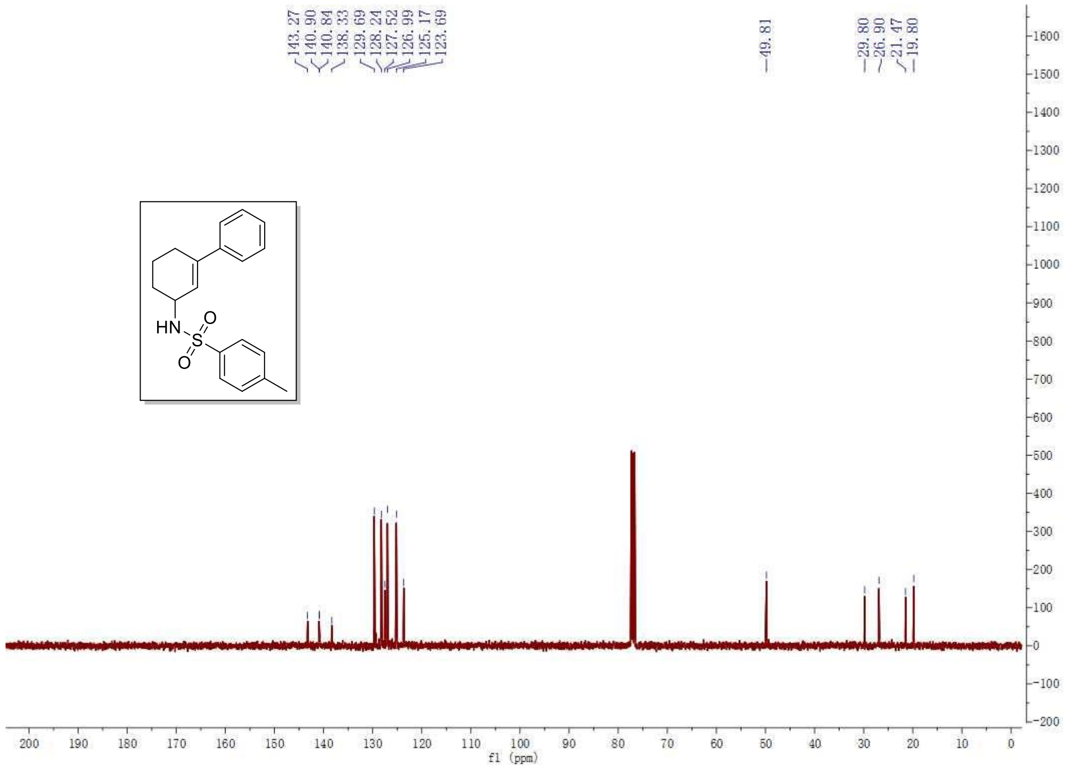

Supplementary Figure 87. ^{13}C NMR spectra for compound 35a

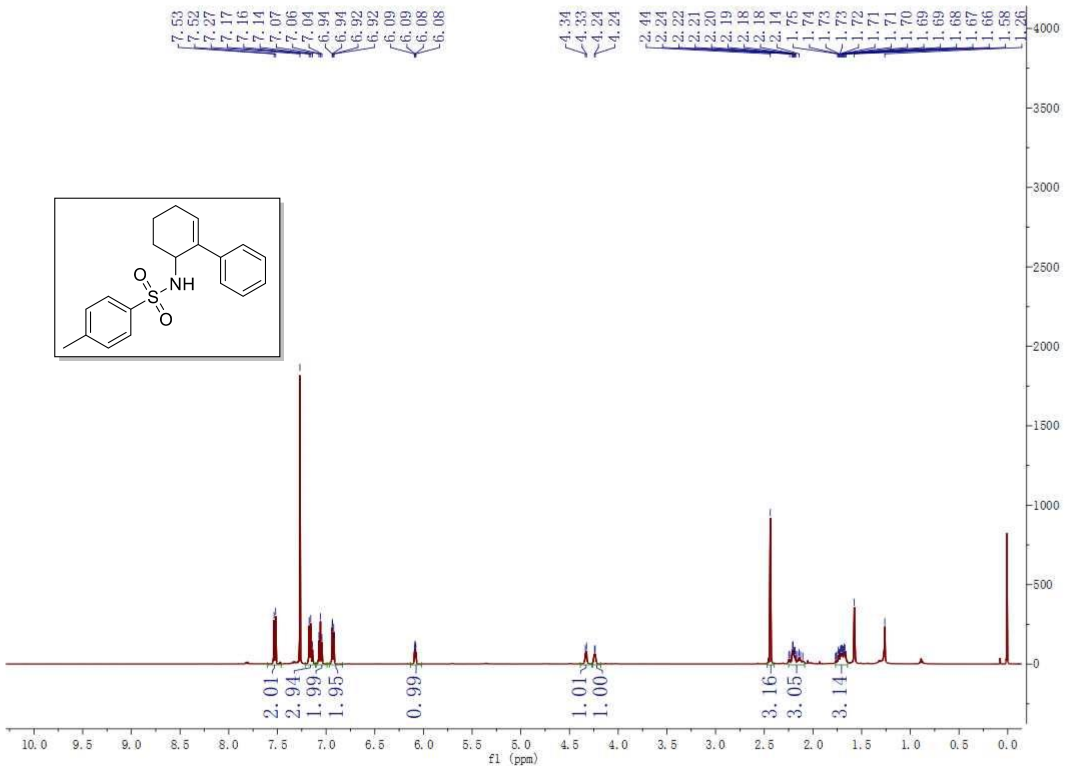

Supplementary Figure 88. ^{1}H NMR spectra for compound 35b

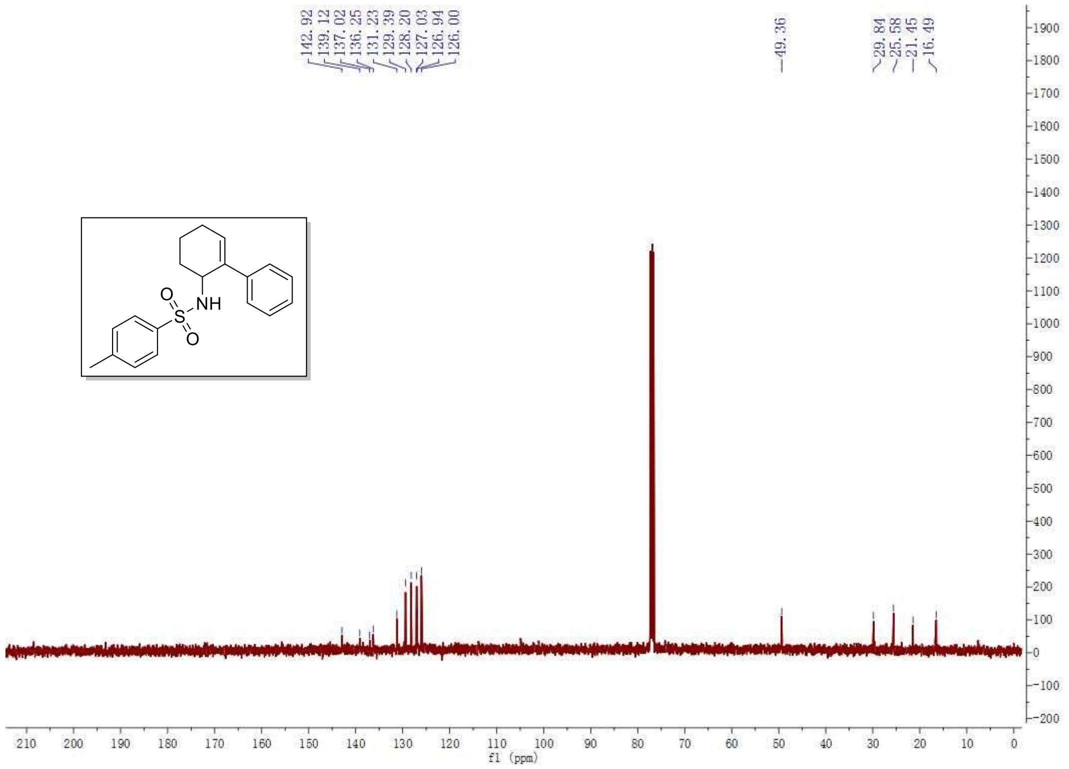

Supplementary Figure 89. ^{13}**C NMR spectra for compound 35b**

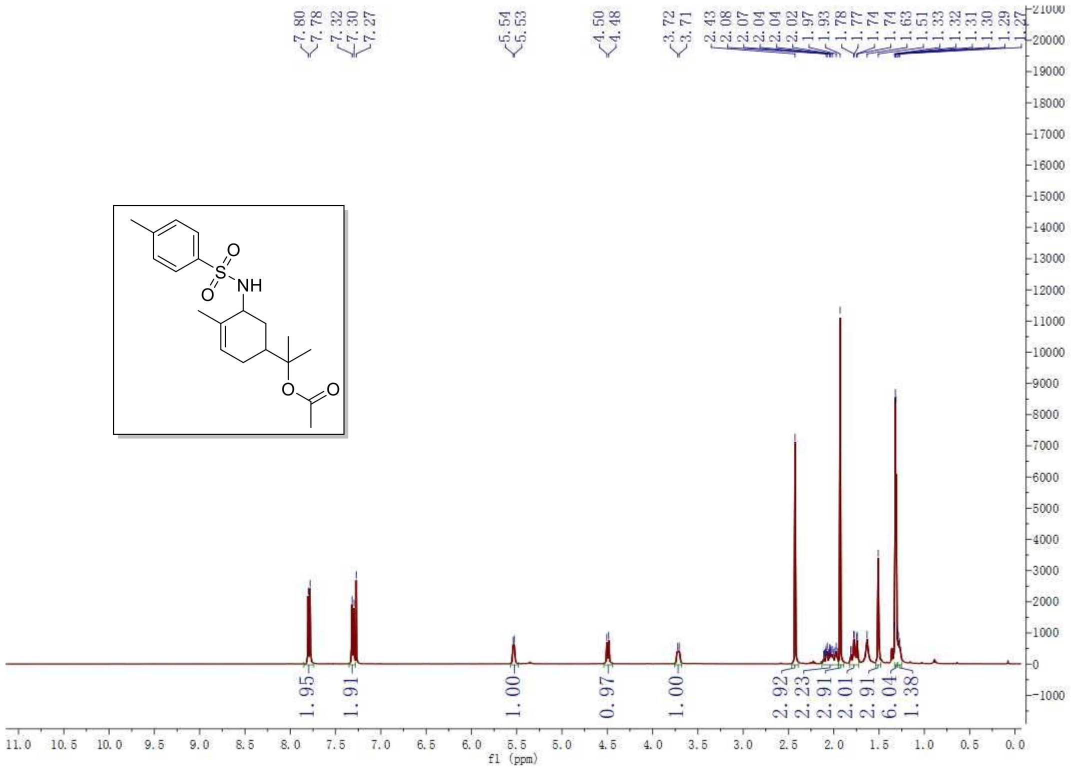

Supplementary Figure 90. ^{1}**H NMR spectra for compound 36c**

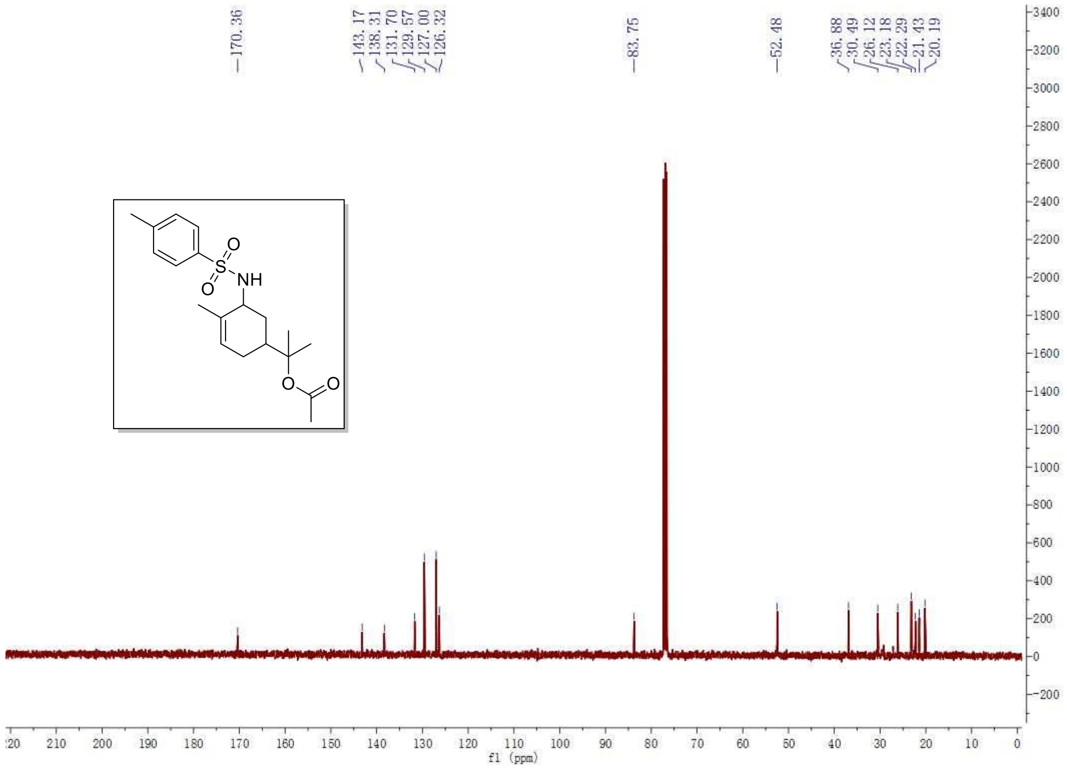

Supplementary Figure 91. ^{13}C NMR spectra for compound 36c

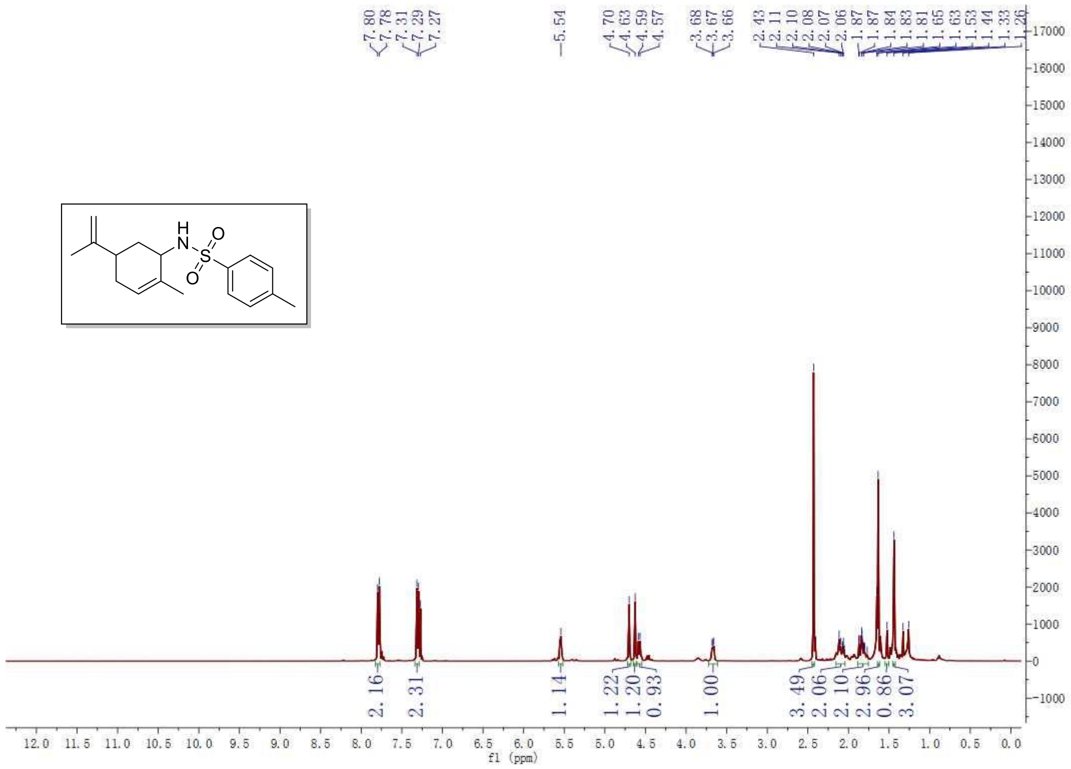

Supplementary Figure 92. ^{1}H NMR spectra for compound 37c

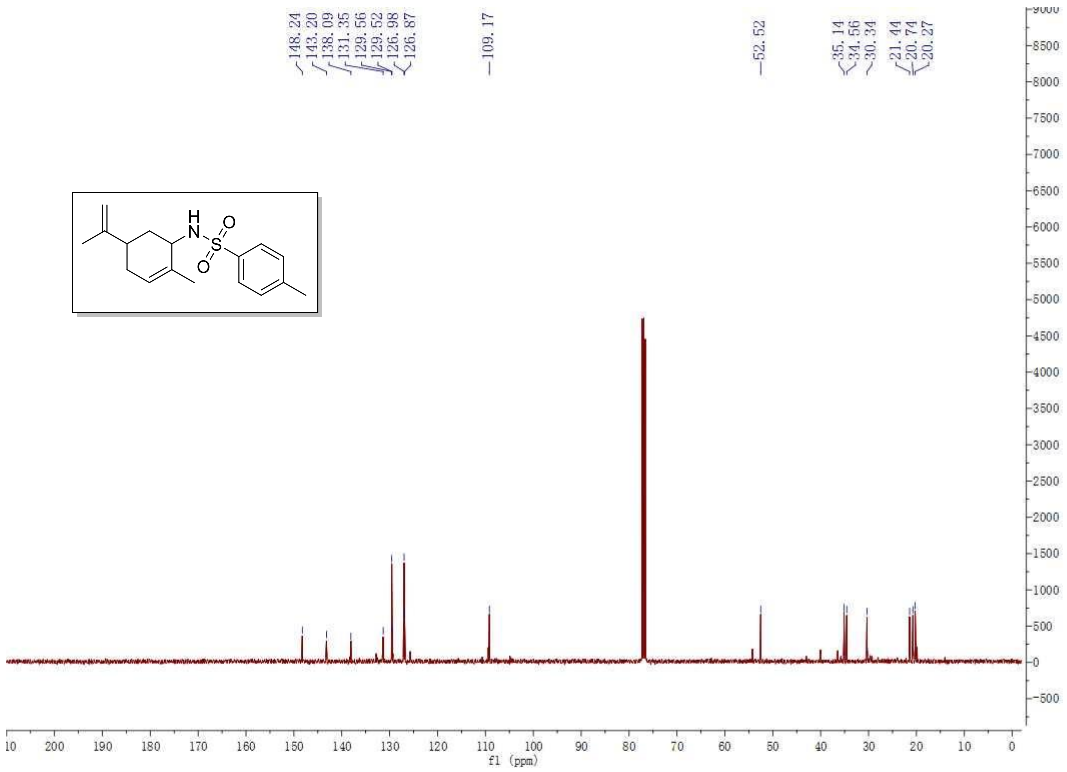

Supplementary Figure 93. ^{13}C NMR spectra for compound 37c

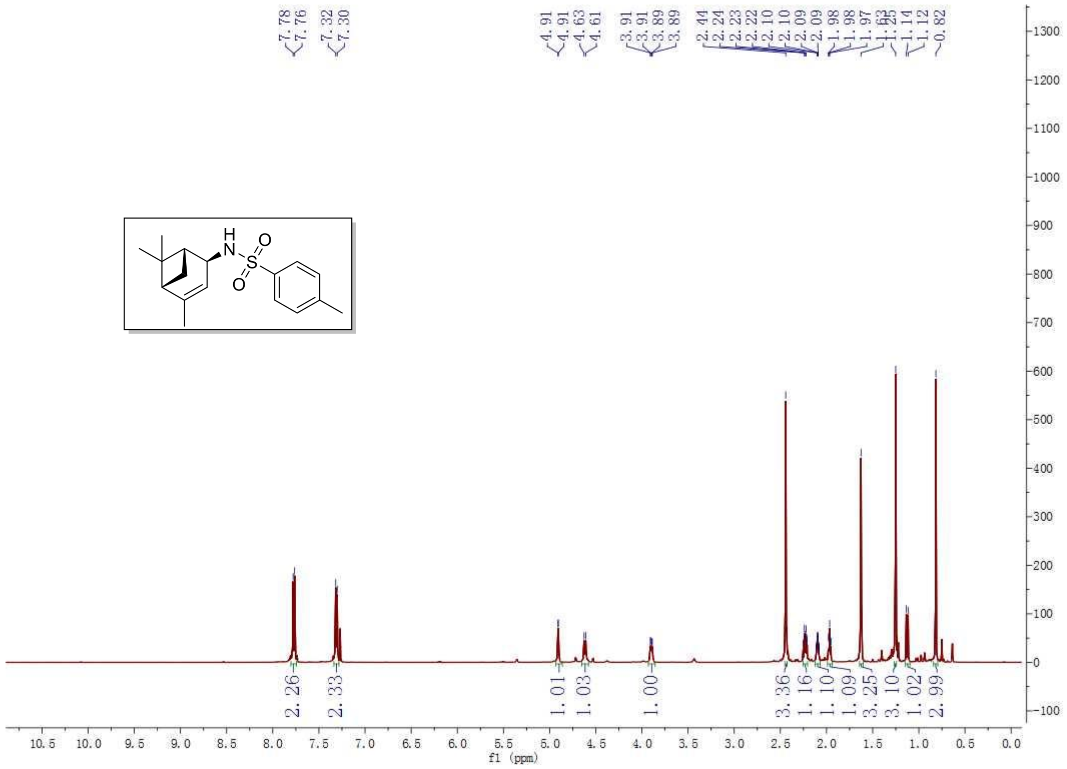

Supplementary Figure 94. ^{1}H NMR spectra for compound 38b

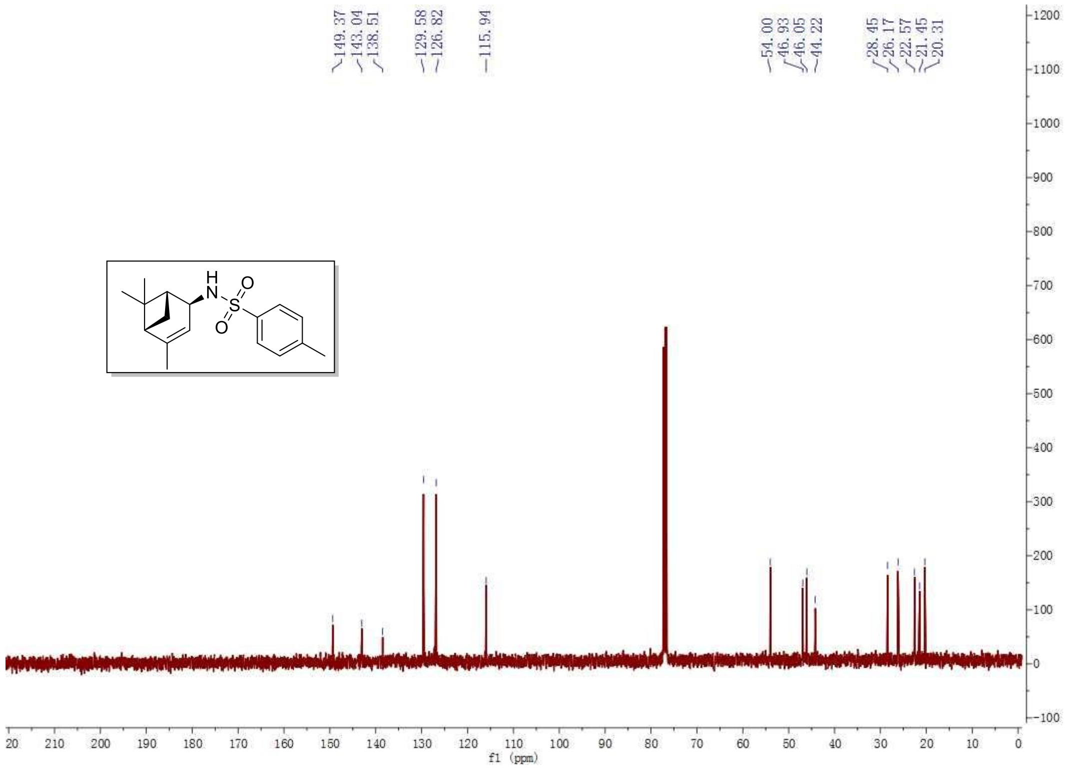

Supplementary Figure 95. ^{13}C NMR spectra for compound 38b

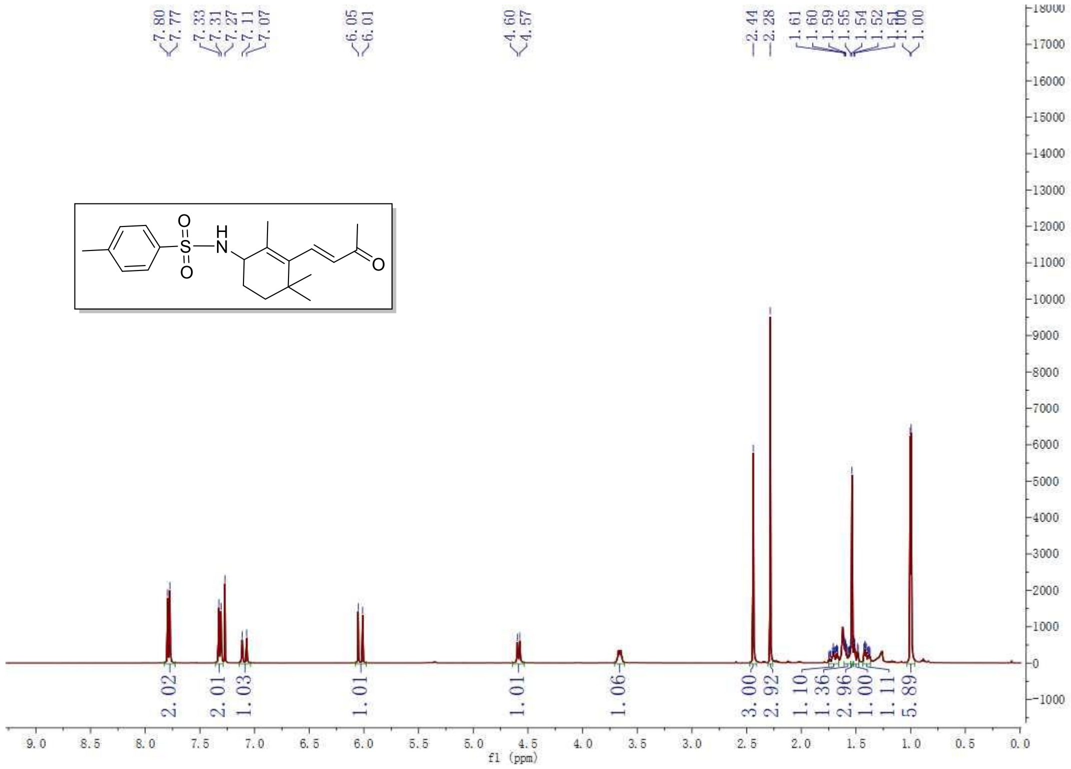

Supplementary Figure 96. ^{1}H NMR spectra for compound 42c

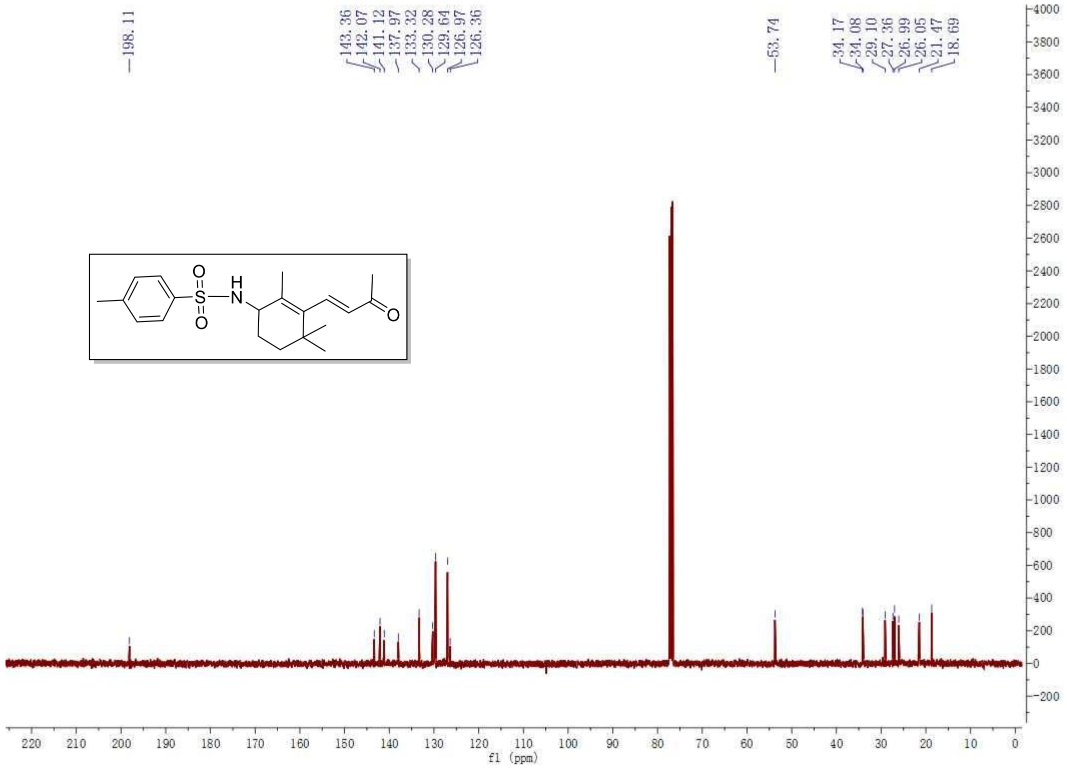

Supplementary Figure 97. ^{13}C NMR spectra for compound 42c

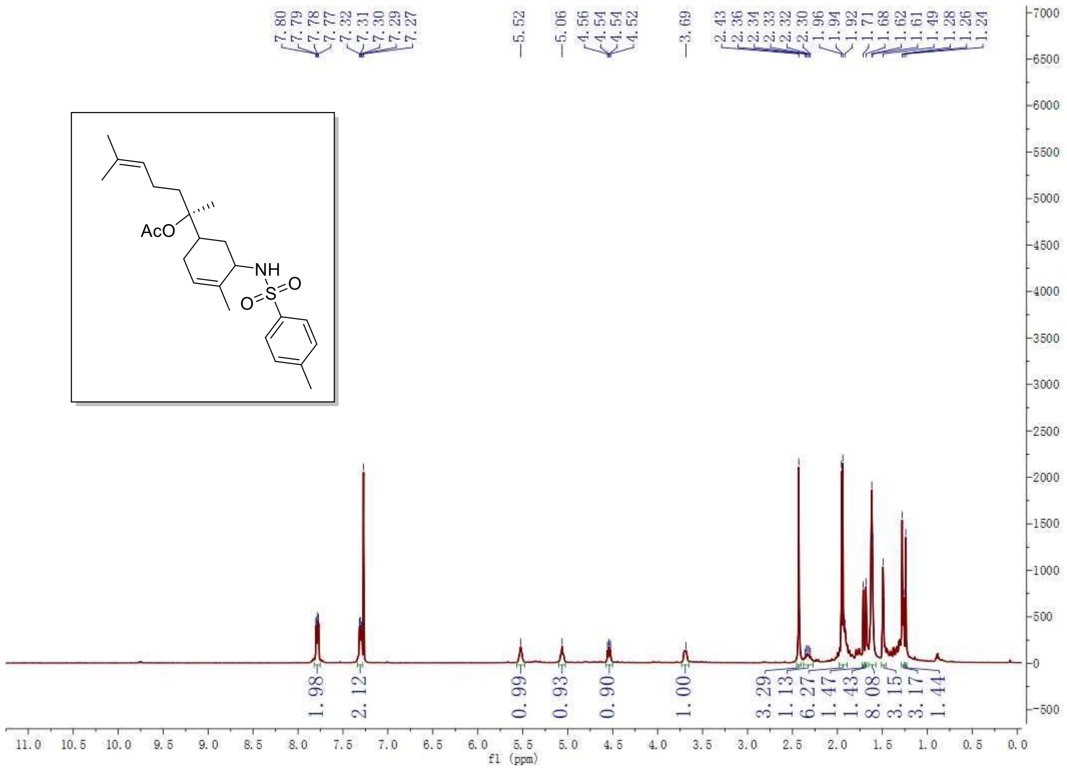

Supplementary Figure 98. ^{1}H NMR spectra for compound 43c

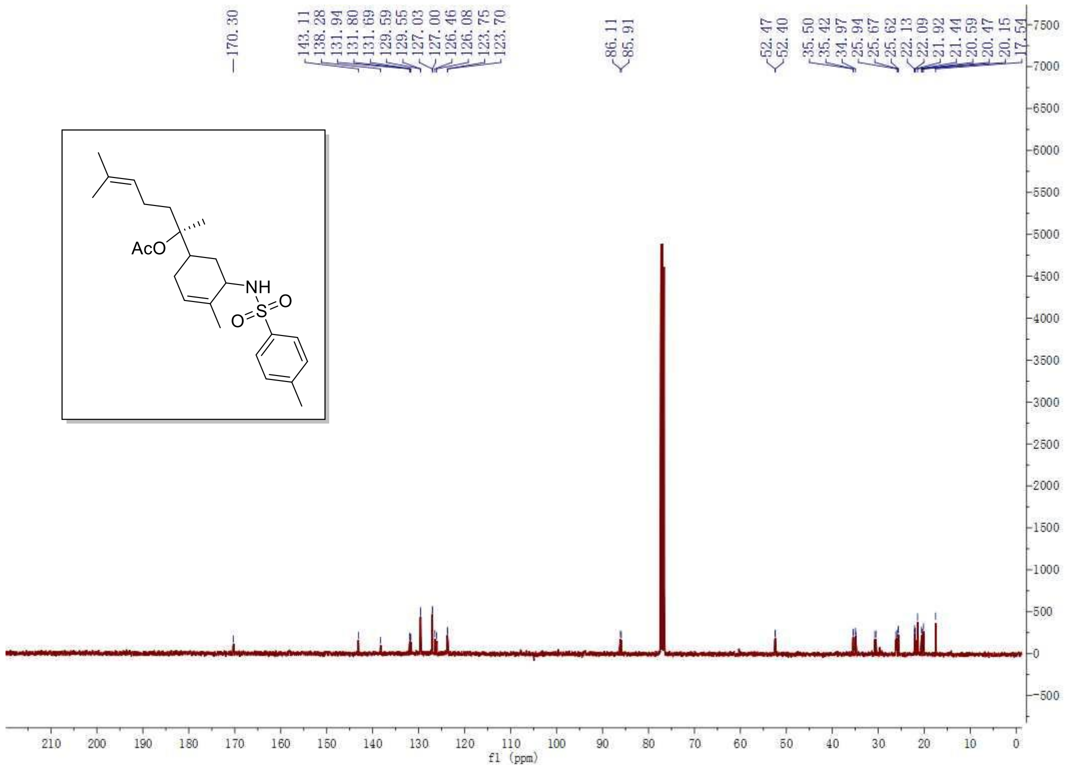

Supplementary Figure 99. ^{13}C NMR spectra for compound 43c

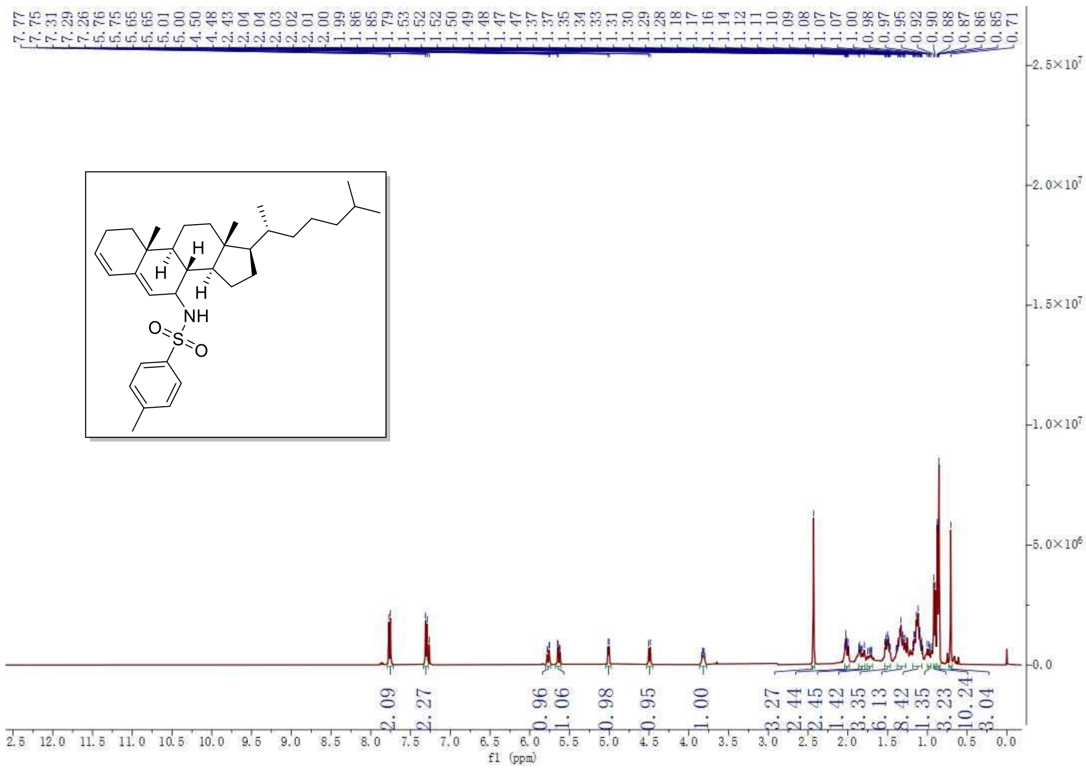

Supplementary Figure 100. ^{1}H NMR spectra for compound 44c

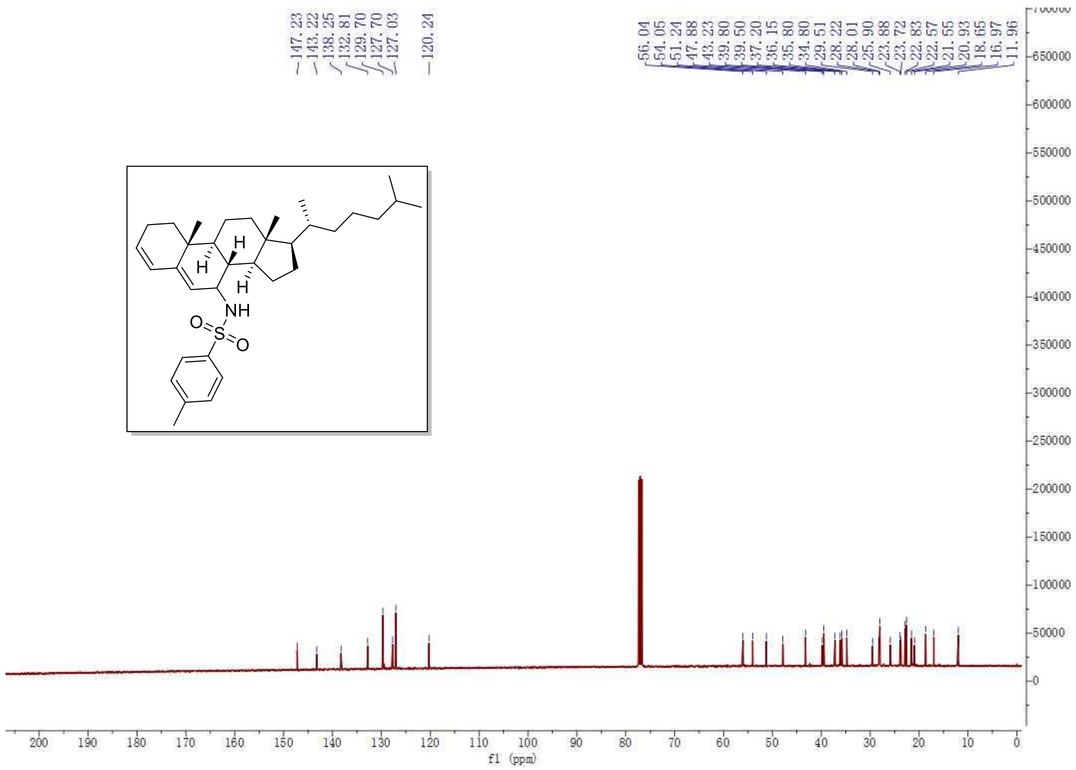

Supplementary Figure 101. ^{13}**C NMR spectra for compound 44c**

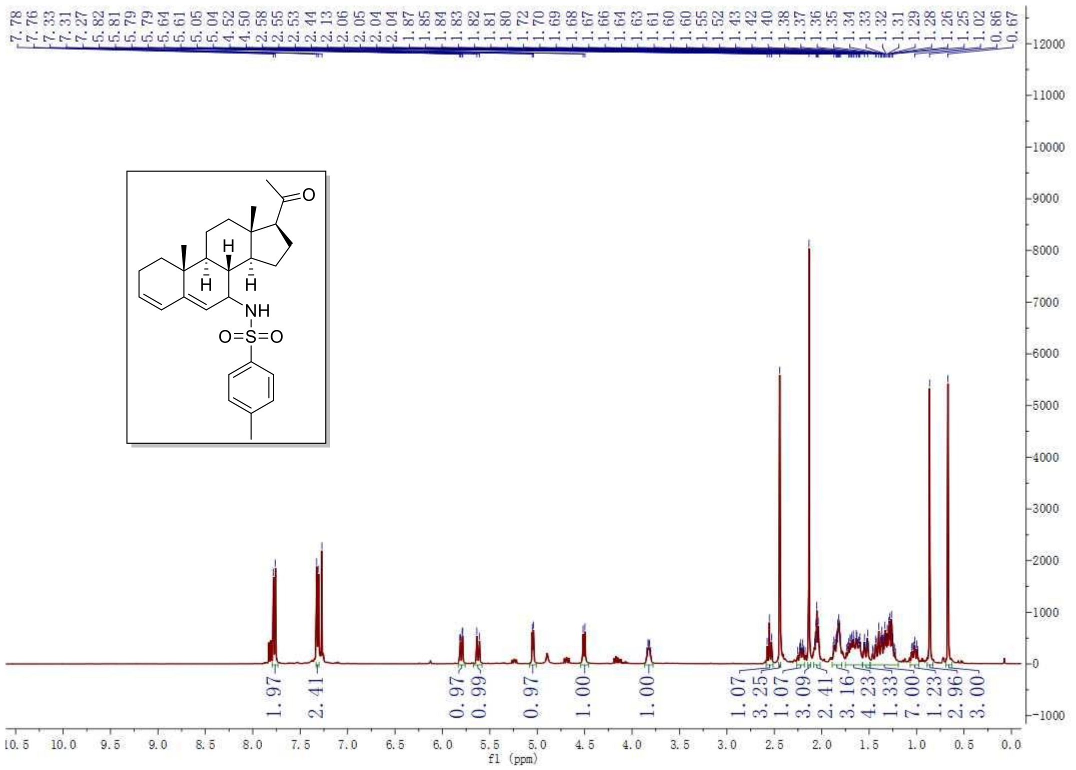

Supplementary Figure 102. ^{1}**H NMR spectra for compound 45c**

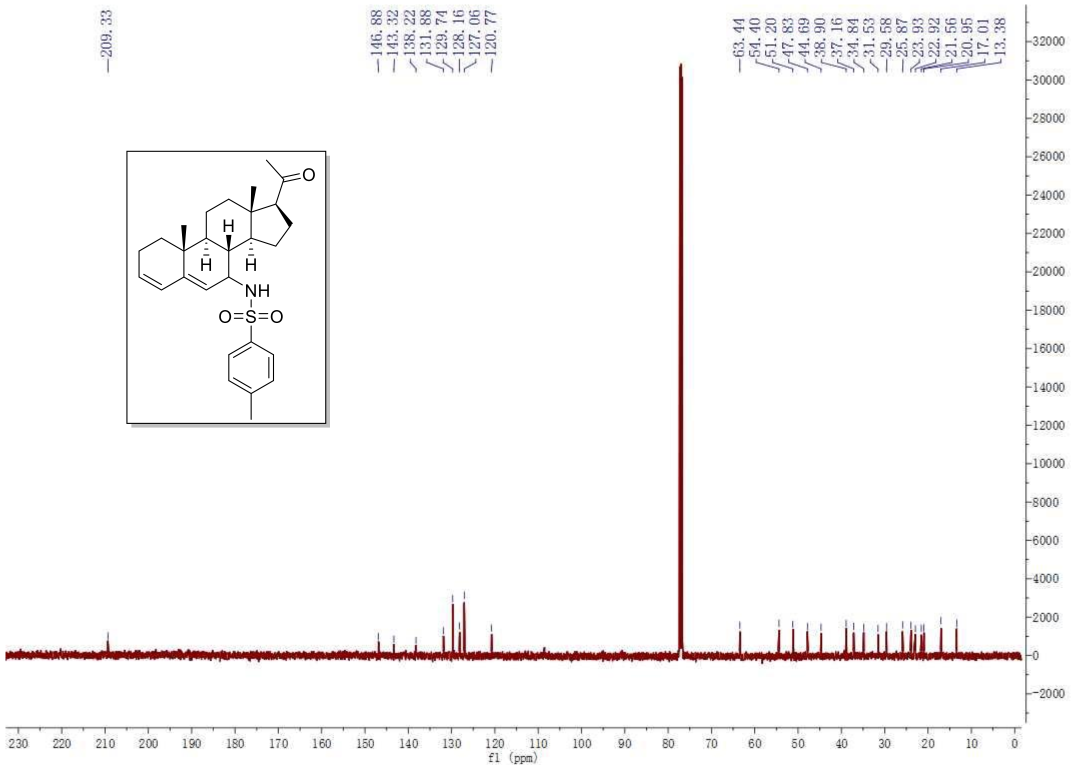

Supplementary Figure 103. ^{13}C NMR spectra for compound 45c

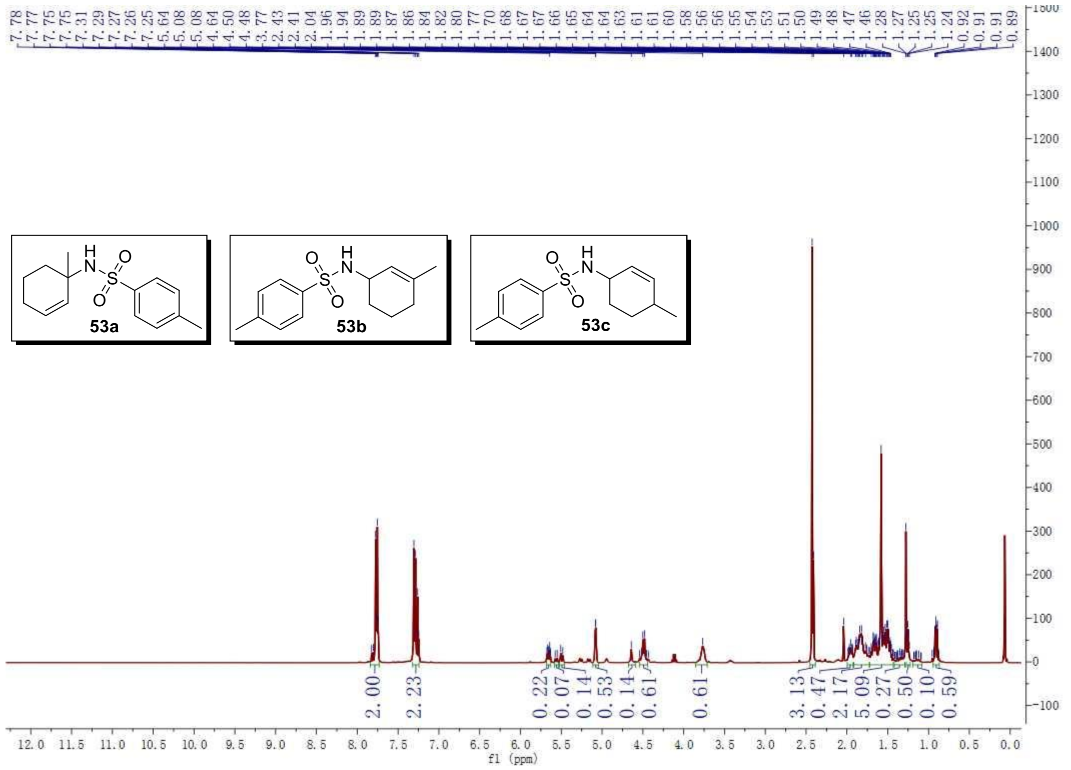

Supplementary Figure 104. ^{1}H NMR spectra for the mixture of 53a, 53b and 53c

The Crystal Structures:

Compound: **(38b)**

CCDC number: 2125791

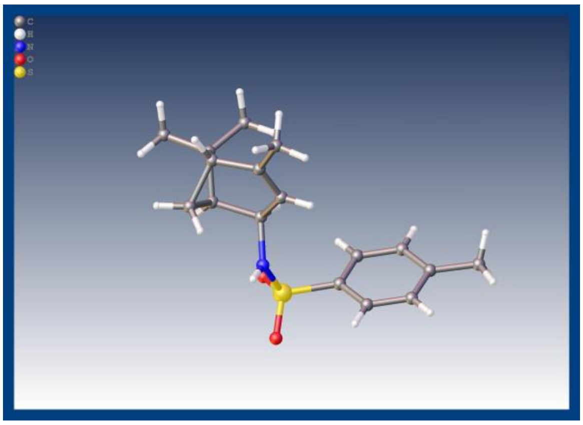

Supplementary Figure 105. The crystal structure of **38b**.

Compound: **(47a)**

CCDC number: 2125792

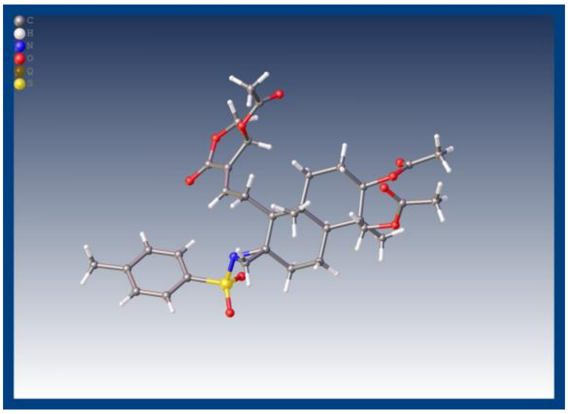

Supplementary Figure 106. The crystal structure of **47a**.

Supplementary References

1. Kobayashi, Y., Masakado, S. & Takemoto, Y. Photoactivated N-Acyliminoiodinanes AppliedtoAmination: an ortho-Methoxymethyl Group Stabilizes ReactivePrecursors. *Angew. Chem. Int. Ed.* **57**, 693 –697 (2018).

2. Mona, C. E., Besserer-Offroy, É., Cabana, J., Leduc, R., Lavigne, P., Heveker, N., Marsault, É. & Escher, E. Design, synthesis, and biological evaluation of CXCR4 ligands. *Org. Biomol. Chem.* **14**, 10298-10311 (2016).

3. Yuan, Q., Yao, K., Liu, D. & Zhang, W. Iridium-catalyzed allyl–allyl cross-coupling of allylic

carbonates with (*E*)-1,3-diarylpropenes. *Chem. Commun.*, **51**, 11834-11836 (2015).
4. Maestre, L., Sameera, W. M. C., Díaz-Requejo, M. M., Maseras, F. & Pérez, P. J. A General Mechanism for the Copper- and Silver-Catalyzed Olefin Aziridination Reactions: Concomitant Involvement of the Singlet and Triplet Pathways. *J. Am. Chem. Soc.* **135**, 1338–1348 (2013).
5. Munnuri, S., Anugu, R. R. & Falck, J. R. Cu(II)-Mediated N–H and N-Alkyl Aryl Amination and Olefin Aziridination. *Org. Lett.* **21**, 1926–1929 (2019).
6. Yamamoto, H., Ho, E., Sasaki, I., Mitsutake, M., Takagi, Y., Imagawa, H. & Nishizawa, M. Intermolecular Amination of Allyl Alcohols with Sulfamates: Effective Utilization of Mercuric Catalyst. *Eur. J. Org. Chem.* **2011**, 2417–2420 (2011).
7. Lee, E. E. & Batey, R. A. Palladium-Catalyzed Allylic Transposition of (Allyloxy) Iminodiazaphospholidines: A Formal [3,3]-Aza-phospha-oxa-Cope Sigmatropic Rearrangement for the Stereoselective Synthesis of Allylic Amines. *J. Am. Chem. Soc.* **127**, 14887–14893 (2005).
8. Silverberg, L. J., Kistler, K. A., Brobst, K., Yennawar, H. P., Lagalante, A., He, G., Ali, K., Blatt, A., Foster, S., Grossman, D., Hegel, S., Minehan, M., Valinsky, D. & Yeasted, J. G. Reactions of the halonium ions of carenes and pinenes: An experimental and theoretical study. *Eur. J. Chem.* **6**, 430-443 (2015).
9. Caballero, G. M. & Gros, E. G. On the Mechanism of Cleavage of Thioacetals Promoted by Copper(II) Sulphate Adsorbed on Silica Gel. *Zeitschrift für Naturforschung B*, **52**, 1147-1151 (1997).
