# Supplementary material for: Combining visible-light induction and copper catalysis for chemo-selective nitrene transfer for late-stage amination of natural products
Source: Commun Chem. 2022 Jul 6;5:79. doi: 10.1038/s42004-022-00692-6 (PMC9814389; doi:10.1038/s42004-022-00692-6)
Supplement: Supplementary file 3 — Description of Additional Supplementary Files [file 42004_2022_692_MOESM3_ESM.pdf]

## Description of Additional Supplementary Files

**File Name:** Supplementary Data 1

**Description:** XRD data
