# Supplementary material for: Combining visible-light induction and copper catalysis for chemo-selective nitrene transfer for late-stage amination of natural products
Source: Commun Chem. 2022 Jul 6;5:79. doi: 10.1038/s42004-022-00692-6 (PMC9814389; doi:10.1038/s42004-022-00692-6)

checkCIF/PLATON report

You have not supplied any structure factors. As a result the full set of tests cannot be run.

THIS REPORT IS FOR GUIDANCE ONLY. IF USED AS PART OF A REVIEW PROCEDURE FOR PUBLICATION, IT SHOULD NOT REPLACE THE EXPERTISE OF AN EXPERIENCED CRYSTALLOGRAPHIC REFEREE.

No syntax errors found. CIF dictionary Interpreting this report

Datablock: 210125031

```
Bond precision:   C-C = 0.0051 A          Wavelength=1.54184

Cell:             a=8.2683(3)     b=17.5477(6)        c=11.8292(4)
                  alpha=90        beta=106.994(3)     gamma=90
Temperature:      180 K

                Calculated                     Reported
Volume          1641.35(10)                    1641.35(10)
Space group     P 21/n                         P 21/n
Hall group      -P 2yn                         -P 2yn
Moiety formula  C17 H23 N O2 S                 ?
Sum formula     C17 H23 N O2 S                 C17 H22 N O2 S
Mr              305.42                         304.41
Dx,g cm-3       1.236                          1.232
Z               4                              4
Mu (mm-1)       1.778                          1.777
F000            656.0                          652.0
F000'           659.00
h,k,lmax        10,22,14                       10,21,14
Nref            3452                           3348
Tmin,Tmax       0.685,0.701                    0.730,1.000
Tmin'           0.559

Correction method= # Reported T Limits: Tmin=0.730 Tmax=1.000
AbsCorr = MULTI-SCAN

Data completeness= 0.970            Theta(max)= 76.474

R(reflections)= 0.0703( 2911)                        wR2(reflections)=
                                                      0.1966( 3348)
S = 1.067                   Npar= 194
```

The following ALERTS were generated. Each ALERT has the format
test-name_ALERT_alert-type_alert-level.
Click on the hyperlinks for more details of the test.

Alert level C

```
ABSTY02_ALERT_1_C  An _exptl_absorpt_correction_type has been given without
            a literature citation. This should be contained in the
            _exptl_absorpt_process_details field.
            Absorption correction given as multi-scan
PLAT041_ALERT_1_C Calc. and Reported SumFormula    Strings  Differ     Please Check
PLAT043_ALERT_1_C Calculated and Reported Mol. Weight Differ by ..       1.01 Check
PLAT068_ALERT_1_C Reported F000 Differs from Calcd (or Missing)...     Please Check
PLAT094_ALERT_2_C Ratio of Maximum / Minimum Residual Density ....       2.09 Report
PLAT230_ALERT_2_C Hirshfeld Test Diff for    C6       --C9       .        5.3 s.u.
PLAT340_ALERT_3_C Low Bond Precision on  C-C Bonds ...............    0.00511 Ang.
PLAT415_ALERT_2_C Short Inter D-H..H-X       H10      ..H16      .       2.13 Ang.
                                                1-x,1-y,1-z  =      3_666 Check
```

Alert level G

```
FORMU01_ALERT_2_G  There is a discrepancy between the atom counts in the
            _chemical_formula_sum and the formula from the _atom_site* data.
            Atom count from _chemical_formula_sum:C17 H22 N1 O2 S1
            Atom count from the _atom_site data:  C17 H23 N1 O2 S1
CELLZ01_ALERT_1_G Difference between formula and atom_site contents detected.
CELLZ01_ALERT_1_G ALERT: Large difference may be due to a
            symmetry error - see SYMMG tests
           From the CIF: _cell_formula_units_Z    4
           From the CIF: _chemical_formula_sum  C17 H22 N O2 S
           TEST: Compare cell contents of formula and atom_site data

           atom    Z*formula  cif sites diff
           C         68.00     68.00    0.00
           H         88.00     92.00   -4.00
           N          4.00      4.00    0.00
           O          8.00      8.00    0.00
           S          4.00      4.00    0.00
PLAT007_ALERT_5_G Number of Unrefined Donor-H Atoms .............          1 Report
PLAT380_ALERT_4_G Incorrectly? Oriented X(sp2)-Methyl Moiety .....        C21 Check
PLAT793_ALERT_4_G Model has Chirality at C1          (Centro SPGR)          R Verify
PLAT793_ALERT_4_G Model has Chirality at C5          (Centro SPGR)          R Verify
PLAT883_ALERT_1_G No Info/Value for _atom_sites_solution_primary .     Please Do !
```

0 **ALERT level A** = Most likely a serious problem - resolve or explain
0 **ALERT level B** = A potentially serious problem, consider carefully
8 **ALERT level C** = Check. Ensure it is not caused by an omission or oversight
8 **ALERT level G** = General information/check it is not something unexpected

7 ALERT type 1 CIF construction/syntax error, inconsistent or missing data
4 ALERT type 2 Indicator that the structure model may be wrong or deficient
1 ALERT type 3 Indicator that the structure quality may be low
3 ALERT type 4 Improvement, methodology, query or suggestion
1 ALERT type 5 Informative message, check

It is advisable to attempt to resolve as many as possible of the alerts in all categories. Often the minor alerts point to easily fixed oversights, errors and omissions in your CIF or refinement strategy, so attention to these fine details can be worthwhile. In order to resolve some of the more serious problems it may be necessary to carry out additional measurements or structure refinements. However, the purpose of your study may justify the reported deviations and the more serious of these should normally be commented upon in the discussion or experimental section of a paper or in the "special_details" fields of the CIF. checkCIF was carefully designed to identify outliers and unusual parameters, but every test has its limitations and alerts that are not important in a particular case may appear. Conversely, the absence of alerts does not guarantee there are no aspects of the results needing attention. It is up to the individual to critically assess their own results and, if necessary, seek expert advice.

Publication of your CIF in IUCr journals

A basic structural check has been run on your CIF. These basic checks will be run on all CIFs submitted for publication in IUCr journals (*Acta Crystallographica*, *Journal of Applied Crystallography*, *Journal of Synchrotron Radiation*); however, if you intend to submit to *Acta Crystallographica Section C* or *E* or *IUCrData*, you should make sure that full publication checks are run on the final version of your CIF prior to submission.

Publication of your CIF in other journals

Please refer to the *Notes for Authors* of the relevant journal for any special instructions relating to CIF submission.

PLATON version of 13/07/2021; check.def file version of 13/07/2021

Datablock 210125031 - ellipsoid plot

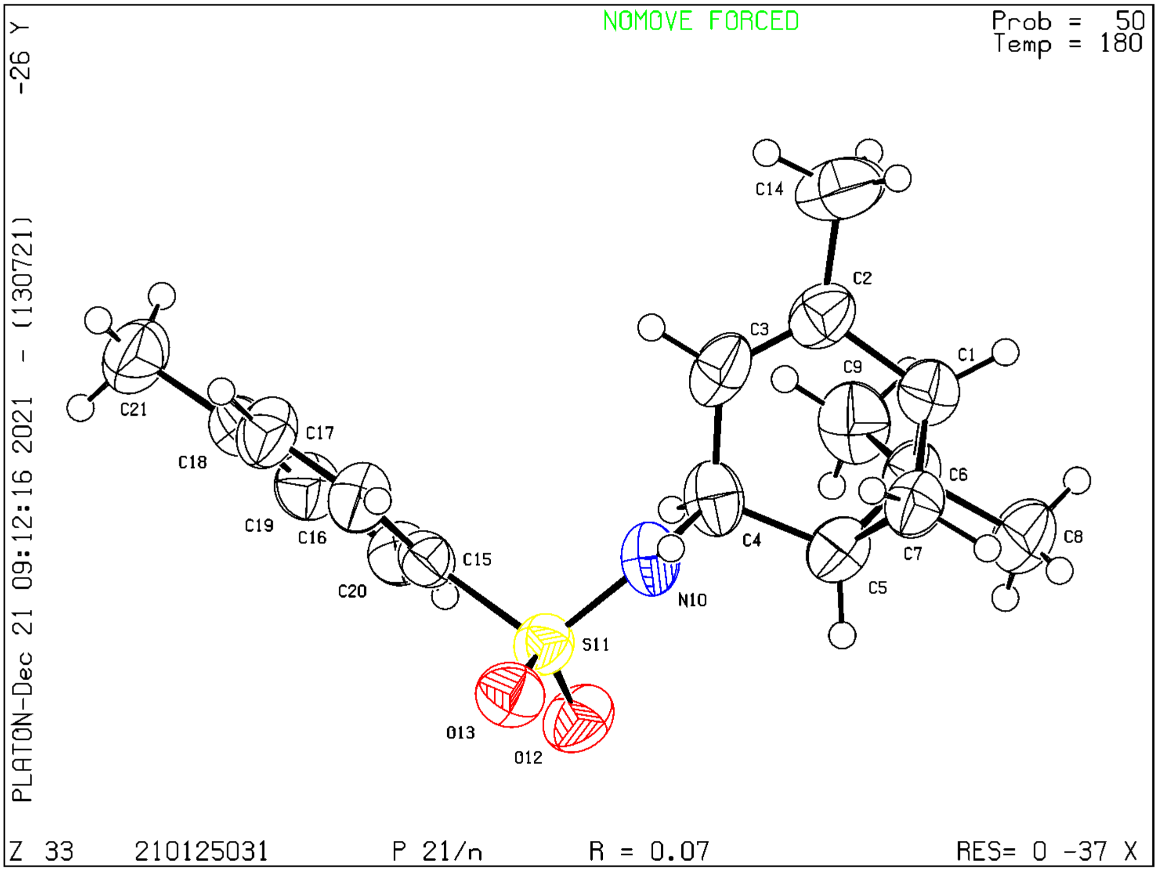

Supplement: Supplementary file 4 — Data Set [file 42004_2022_692_MOESM4_ESM.zip › XRD data for products/checkcif for 38b.pdf]
