# Supplementary material for: Combining visible-light induction and copper catalysis for chemo-selective nitrene transfer for late-stage amination of natural products
Source: Commun Chem. 2022 Jul 6;5:79. doi: 10.1038/s42004-022-00692-6 (PMC9814389; doi:10.1038/s42004-022-00692-6)

checkCIF/PLATON report

You have not supplied any structure factors. As a result the full set of tests cannot be run.

THIS REPORT IS FOR GUIDANCE ONLY. IF USED AS PART OF A REVIEW PROCEDURE FOR PUBLICATION, IT SHOULD NOT REPLACE THE EXPERTISE OF AN EXPERIENCED CRYSTALLOGRAPHIC REFEREE.

No syntax errors found. CIF dictionary Interpreting this report

Datablock: 210126008

```
Bond precision:   C-C = 0.0035 A          Wavelength=1.54184

Cell:             a=13.7437(2)     b=7.6926(1)          c=15.7757(2)
                  alpha=90         beta=102.913(1)      gamma=90
Temperature:      180 K

                 Calculated                    Reported
Volume           1625.70(4)                    1625.70(4)
Space group      P 21                          P 21
Hall group       P 2yb                         P 2yb
Moiety formula   C33 H43 N O10 S               ?
Sum formula      C33 H43 N O10 S               C33 H43 N O10 S
Mr               645.74                        645.74
Dx,g cm-3        1.319                         1.319
Z                2                             2
Mu (mm-1)        1.375                         1.375
F000             688.0                         688.0
F000'            690.83
h,k,lmax         17,9,19                       17,9,19
Nref             6836[ 3682]                   6550
Tmin,Tmax        0.755,0.760                   0.849,1.000
Tmin'            0.630

Correction method= # Reported T Limits: Tmin=0.849 Tmax=1.000
AbsCorr = MULTI-SCAN

Data completeness= 1.78/0.96       Theta(max)= 76.678

R(reflections)= 0.0334( 6324)                          wR2(reflections)=
                                                       0.0937( 6550)
S = 1.066                    Npar= 413
```

The following ALERTS were generated. Each ALERT has the format
test-name_ALERT_alert-type_alert-level.
Click on the hyperlinks for more details of the test.

Alert level C

```
ABSTY02_ALERT_1_C  An _exptl_absorpt_correction_type has been given without
            a literature citation. This should be contained in the
            _exptl_absorpt_process_details field.
            Absorption correction given as multi-scan
```

Alert level G

```
PLAT791_ALERT_4_G Model has Chirality at C1          (Sohnke SpGr)          S Verify
PLAT791_ALERT_4_G Model has Chirality at C2          (Sohnke SpGr)          S Verify
PLAT791_ALERT_4_G Model has Chirality at C3          (Sohnke SpGr)          R Verify
PLAT791_ALERT_4_G Model has Chirality at C6          (Sohnke SpGr)          R Verify
PLAT791_ALERT_4_G Model has Chirality at C7          (Sohnke SpGr)          R Verify
PLAT791_ALERT_4_G Model has Chirality at C8          (Sohnke SpGr)          S Verify
PLAT791_ALERT_4_G Model has Chirality at C19         (Sohnke SpGr)          S Verify
PLAT883_ALERT_1_G No Info/Value for _atom_sites_solution_primary .     Please Do !
```

0 **ALERT level A** = Most likely a serious problem - resolve or explain
0 **ALERT level B** = A potentially serious problem, consider carefully
1 **ALERT level C** = Check. Ensure it is not caused by an omission or oversight
8 **ALERT level G** = General information/check it is not something unexpected

2 ALERT type 1 CIF construction/syntax error, inconsistent or missing data
0 ALERT type 2 Indicator that the structure model may be wrong or deficient
0 ALERT type 3 Indicator that the structure quality may be low
7 ALERT type 4 Improvement, methodology, query or suggestion
0 ALERT type 5 Informative message, check

It is advisable to attempt to resolve as many as possible of the alerts in all categories. Often the minor alerts point to easily fixed oversights, errors and omissions in your CIF or refinement strategy, so attention to these fine details can be worthwhile. In order to resolve some of the more serious problems it may be necessary to carry out additional measurements or structure refinements. However, the purpose of your study may justify the reported deviations and the more serious of these should normally be commented upon in the discussion or experimental section of a paper or in the "special_details" fields of the CIF. checkCIF was carefully designed to identify outliers and unusual parameters, but every test has its limitations and alerts that are not important in a particular case may appear. Conversely, the absence of alerts does not guarantee there are no aspects of the results needing attention. It is up to the individual to critically assess their own results and, if necessary, seek expert advice.

Publication of your CIF in IUCr journals

A basic structural check has been run on your CIF. These basic checks will be run on all CIFs submitted for publication in IUCr journals (*Acta Crystallographica*, *Journal of Applied Crystallography*, *Journal of Synchrotron Radiation*); however, if you intend to submit to *Acta Crystallographica Section C* or *E* or *IUCrData*, you should make sure that full publication checks are run on the final version of your CIF prior to submission.

Publication of your CIF in other journals

Please refer to the *Notes for Authors* of the relevant journal for any special instructions relating to CIF submission.

PLATON version of 13/07/2021; check.def file version of 13/07/2021

Datablock 210126008 - ellipsoid plot

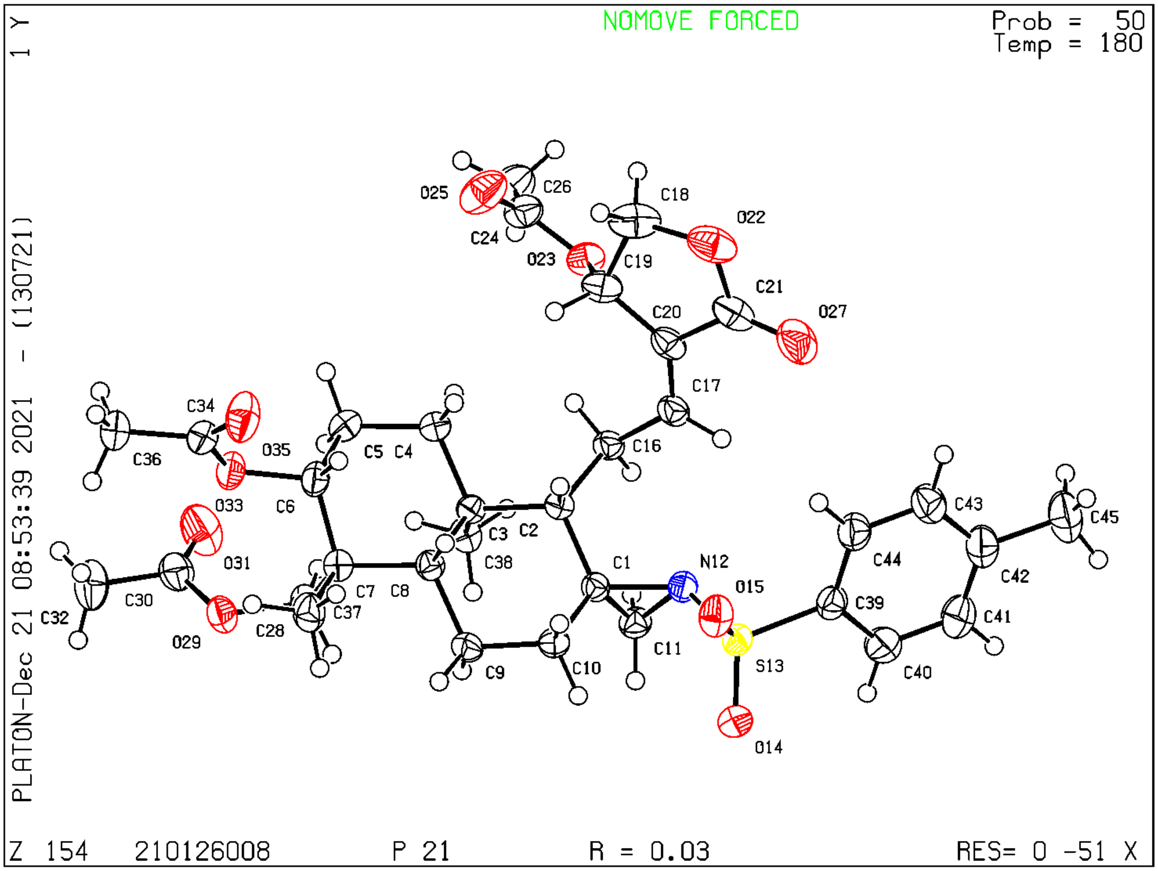

Supplement: Supplementary file 4 — Data Set [file 42004_2022_692_MOESM4_ESM.zip › XRD data for products/checkcif for 47a.pdf]
